# Phosphoglycerate kinase 1 cooperates with the AP2 complex to promote endocytosis

**DOI:** 10.1038/s44319-026-00948-5

**Published:** 2026-10-01

**Authors:** Shao-Ling Chu, Yu-Tzu Chang, Che-Yu Hung, Chia-Jung Yu, Jia-Wei Hsu

**Affiliations:** 1 Institute of Biochemical Sciences, National Taiwan University, Taipei, Taiwan; 2 Department of Cell and Molecular Biology, College of Medicine, Chang Gung University, Taoyuan, Taiwan; 3 Department of Thoracic Medicine, Chang Gung Memorial Hospital, Taoyuan, Taiwan; 4 Institute of Biological Chemistry, Academia Sinica, Taipei, Taiwan

**Keywords:** Cell Adhesion, Polarity & Cytoskeleton, Membranes & Trafficking, Organelles

## Abstract

Adaptor proteins play a critical role in intracellular protein transport by binding to cargoes and facilitating their sorting into vesicles. The adaptor protein 2 (AP2) complex specifically recognizes sorting motifs on cargo proteins such as epidermal growth factor receptor (EGFR) and transferrin receptor (TfR) for proper cargo recognition. However, the coordination of adaptor proteins in cargo recognition remains unclear. Here, we uncover the non-canonical role of glycolytic enzyme phosphoglycerate kinase 1 (PGK1) in protein endocytosis, acting independently of its catalytic activity. PGK1 serves as a co-adaptor to directly engage cargoes and reinforce AP2 complex stability, orchestrating efficient cargo recognition to promote endocytosis. Furthermore, PGK1 also integrates into the AP2-clathrin complex, enhancing cargo recognition and internalization. Collectively, our findings establish a previously unidentified coat protein complex containing PGK1, highlighting its dual role as a cargo adaptor and stabilizer of the AP2 complex.

## Synopsis

PGK1 interacts with and stabilizes the AP2 complex and acts as a cargo adaptor for clathrin-mediated endocytosis.

- PGK1 directly interacts with α subunit of AP2 and stabilizes the AP2 complex.
- PGK1 directly interacts with cargo proteins and recognizes the dileucine and the tyrosine-based sorting motifs in TfR and EGFR, respectively.
- The association of PGK1 and AP2 complex facilitates the endocytic cargo recognition process.
- PGK1 is a part of the clathrin-coated complex to mediate protein endocytosis.

## Abstract (web summary)

PGK1 interacts with and stabilizes the AP2 complex and acts as a cargo adaptor for clathrin-mediated endocytosis.

## Graphical abstract

**Figure:**
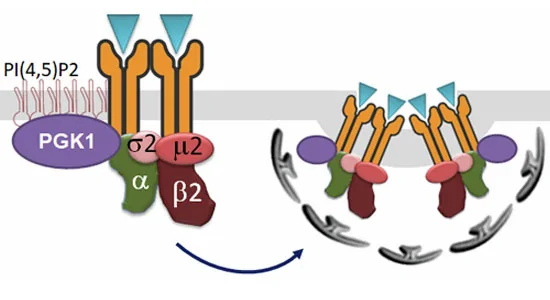

## Introduction

Coat proteins play a central role in intracellular protein transport by binding to cargoes and facilitating their sorting into specific vesicles (Hsu et al, 2012; Sigismund et al, 2021). Cargo proteins interact directly with different coat proteins, establishing their dynamic movement between organelle compartments within the secretory and endocytic pathways (Paczkowski et al, 2015). The best characterized coat protein for controlling endocytosis is the clathrin-adaptor protein 2 (AP2) coat complex (Bonifacino and Lippincott-Schwartz, 2003). The clathrin triskelion couples with the AP2 complex to form the clathrin-AP2 coat complex, which functions in protein endocytosis (Boll et al, 1996; Boucrot et al, 2010; Rapoport et al, 1997). The AP2 adaptor protein complex consists of two large subunits (α and β2), a medium subunit (µ2), and a small subunit (σ2). Two of the best characterized sorting motifs on cargo proteins, the acidic dileucine motif and tyrosine-based motif on epidermal growth factor receptor (EGFR) and the tyrosine-based motif on transferrin receptor (TfR), can be specifically recognized by the AP2 complex (Traub, 2003). The acidic dileucine motif binds to σ2, whereas the tyrosine-based motif interacts with µ2 (Doray et al, 2007; Motley et al, 2006). Upon being recruited to the plasma membrane, the AP2 complex recognizes sorting signals on cargo proteins and subsequently recruits clathrin to facilitate vesicle formation. During this process, multiple accessory proteins interact with the AP2 complex directly or indirectly to form the clathrin-AP2 coat complex (Traub, 2003). These proteins, with the AP2 complex as the central component, form an interconnected system to regulate the dynamics of protein endocytosis. However, how these adaptor proteins coordinate to specifically recognize cargo proteins remains unclear.

Phosphoglycerate kinase 1 (PGK1) is a metabolic enzyme that catalyses adenosine triphosphate (ATP) production during glycolysis. In addition to its canonical role in glycolysis, PGK1 functions as a protein kinase in tumorigenesis and autophagy (Li et al, 2016; Qian et al, 2017). By further investigating endocytic protein transport, we previously identified that PGK1 directly interacts with the dileucine sorting motif on EGFR to promote its lysosomal protein transport (Chu et al, 2024). We also found that the ability of PGK1 to promote EGFR lysosomal transport is independent of its kinase activity, indicating that its moonlighting function does not require its metabolic activity (Chu et al, 2024). Here, we demonstrate that PGK1 unexpectedly has a moonlighting function in regulating protein endocytosis. We show that the PGK1 and AP2 complex specifically recognize sorting motifs on cargo proteins to increase binding avidity via multivalent interactions and further recruit clathrin to account for endocytosis. These mechanisms provide functional parallels of PGK1 to the lysosomal protein transport pathway. Overall, our study revealed a clathrin protein complex containing PGK1 as the cargo adaptor in cooperation with AP2 complex for proper cargo recognition during protein endocytosis.

## Results

### PGK1 promotes AP2-dependent endocytosis

**Figure 1:**
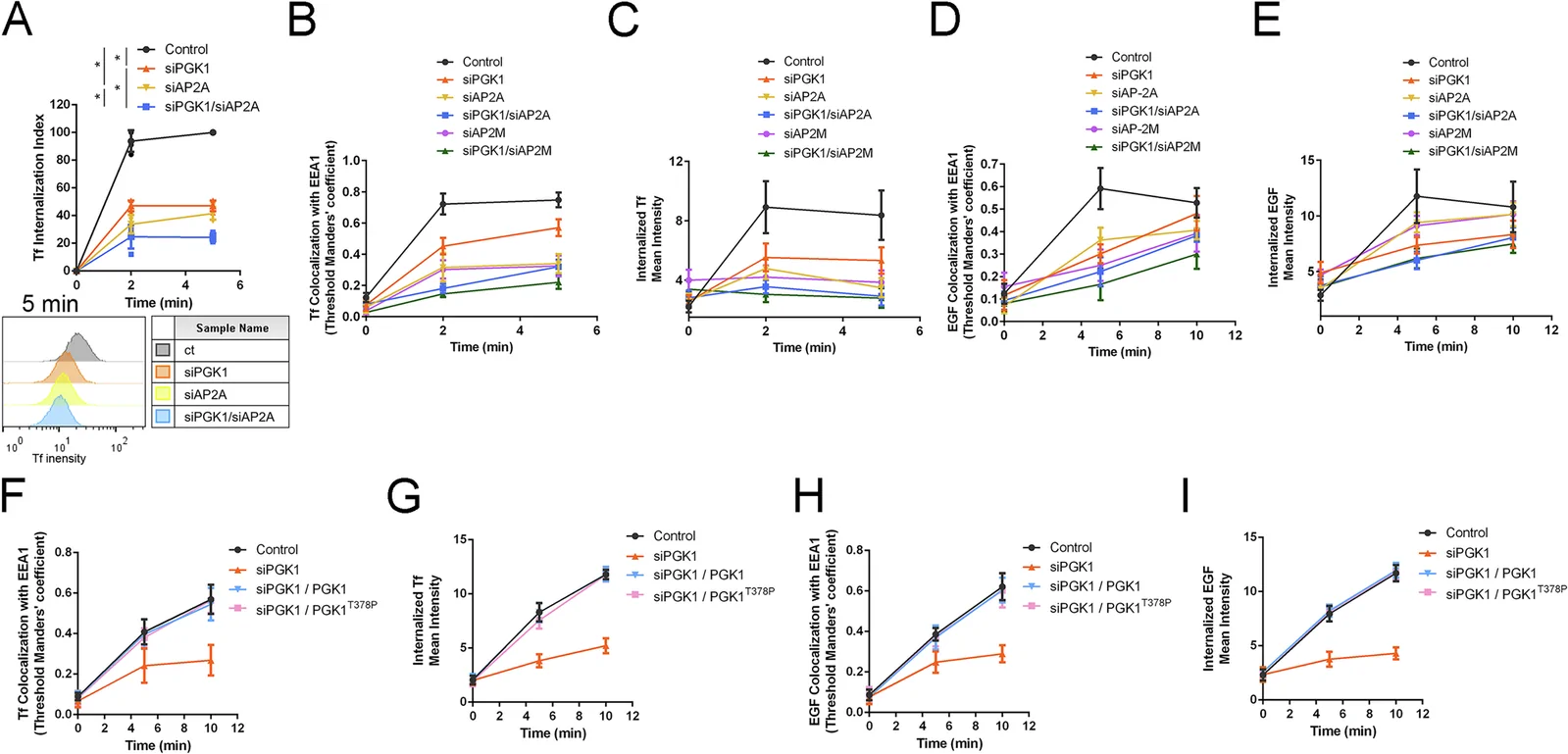
PGK1 regulates AP2-mediated endocytosis independently of its kinase activity. Quantitative analyses of cells treated with the indicated siRNAs are presented as means ± standard deviations (SDs). *P < 0.01; (**A**) Internalization of Tf was quantified by flow cytometry using trypan blue quenching at 0, 2, and 5 min (n > 10,000 cells, four independent biological replicates). One-way ANOVA with Tukey’s test at the 5-min time point: control vs. siPGK1 (*P* = 0.0003); control vs. siAP2A (*P* = 0.0004); siAP2A vs. siPGK1/siAP2A (*P* = 0.0253); siPGK1 vs. siPGK1/siAP2A (*P* = 0.0081). (**B**) Colocalization of Tf with EEA1, expressed as the thresholded Manders’ coefficients, was quantified at 0, 2, and 5 min (*n* = 20 cells of one representative experiment from independent two biological replicates). (**C**) Mean intensity of internalized Tf was assessed at 0, 2, and 5 min (*n* = 20 cells of one representative experiment from independent two biological replicates). (**D**) Colocalization of EGF with EEA1, expressed as thresholded Manders’ coefficients, was quantified at 0, 5, and 10 min (*n* = 20 cells of one representative experiment from independent two biological replicates). (**E**) Mean intensity of internalized EGF was assessed at 0, 5, and 10 min (*n* = 25 cells of one representative experiment from independent two biological replicates). (**F**) Colocalization of Tf with EEA1, expressed as thresholded Manders’ coefficients, was performed to compare the effects of PGK1 and PGK1^T378P^ at 0, 5, and 10 min (*n* = 15 cells of one representative experiment from independent two biological replicates). (**G**) Mean intensity of internalized Tf was measured to compare the effects of PGK1 and PGK1^T378P^ at 0, 5, and 10 min (*n* = 15 cells of one representative experiment from independent two biological replicates). (**H**) Colocalization of EGF with EEA1, expressed as thresholded Manders’ coefficients, was performed to compare the effects of PGK1 and PGK1^T378P^ at 0, 5, and 10 min (*n* = 15 cells of one representative experiment from independent two biological replicates). (**I**) Mean intensity of internalized EGF was performed to compare the effects of PGK1 and PGK1^T378P^ at 0, 5, and 10 min (*n* = 15 cells of one representative experiment from independent two biological replicates). Source data are available online for this figure.

We previously reported that PGK1 acts as a cargo adaptor to promote the lysosomal transport of EGFR upon EGF stimulation (Chu et al, 2024). To understand the additional role of PGK1 in endocytic transport, we first performed coimmunoprecipitation-coupled mass spectrometry to identify PGK1-interacting proteins, and identified one potential target, AP2 complex, from the top hit of our proteomic analysis (Dataset EV1). The AP2 complex regulates major clathrin-dependent endocytosis events, including the endocytosis of many cellular receptors, such as transferrin receptor (TfR) and epidermal growth factor receptor (EGFR). Interference with the AP2 component significantly impairs the endocytosis of TfR and EGFR (Goh et al, 2010; Motley et al, 2006). Notably however, a previous report demonstrated that some endocytosis events still occur in siAP2-treated cells (Motley et al, 2003), leading us to consider additional or alternative factor(s) that can cooperate with the AP2 complex to control endocytosis. We hypothesized that PGK1 may cooperate with the AP2 complex to participate in AP2-dependent endocytosis. We first examined TfR endocytosis in cells by using flow cytometry to detect internalized fluorescein-labelled Tf signals combined with the use of Trypan blue to quench plasma membrane-bound forms (Gulbranson et al, 2019; Patino et al, 2015). We then found that single knockdown of AP2A or PGK1 significantly impaired TfR endocytosis, and co-knockdown of AP2A and PGK1 showed synergistic effect on the inhibition of endocytosis (Fig. 1A). We next addressed the kinetic analysis of endocytosis by tracking the cargo internalization of Tf-labelled TfRs into early endosomes combined with acid wash to remove pre-bound ligands on the cell surface, and also revealed a defect in TfR endocytosis in AP2A and PGK1 co-knockdown cells (Fig. 1B,C; Appendix Fig. S1A,B). The exacerbation of EGFR endocytosis defects was also detected in AP2A and PGK1 co-knockdown cells compared with single knockdown cells (Fig. 1D,E; Appendix Fig. S1C). In addition, we also confirmed the involvement of the μ2 subunit of the AP2 complex to cause the severe defect in AP2-dependent endocytosis by tracking the internalization of TfR and EGFR in cells in which both AP2M and PGK1 were largely depleted (Fig. 1B–E; Appendix Fig. S1B,C). As a control, we also showed that the internalization of the cholera toxin B subunit (CTxB) was not affected in AP2A and PGK1 co-knockdown cells (Appendix Fig. S1D). To avoid the effects of low temperature during ligand prebinding, we simply treated the ligand at 37 °C to track receptor endocytosis (Gulbranson et al, 2019). Consistently, we also observed that the uptake of TfR was significantly abolished in AP2A and PGK1 co-knockdown cells compared with that in siPGK1 or siAP2A-treated cells (Appendix Fig. S1E). These results demonstrated that PGK1 and α subunit of AP2 complex synergistically participate in protein endocytosis. We further determined that the glycolytic activity of PGK1 was not required for AP2-dependent endocytosis because both the wild-type PGK1 and the catalytic mutant PGK1-T378P successfully restored TfR endocytosis in siPGK1-treated cells (Fig. 1F,G; Appendix Fig. S1F,G), and for the restoration of EGFR endocytosis in cells subjected to PGK1 knockdown was also observed (Fig. 1H,I; Appendix Fig. S1H). Together, these data suggest that PGK1 can be the essential protein factor to regulate protein endocytosis independent of its catalytic activity, especially when the function of the AP2 complex is impaired.

### PGK1 interacts with α subunit and stabilizes the AP2 complex

**Figure 2:**
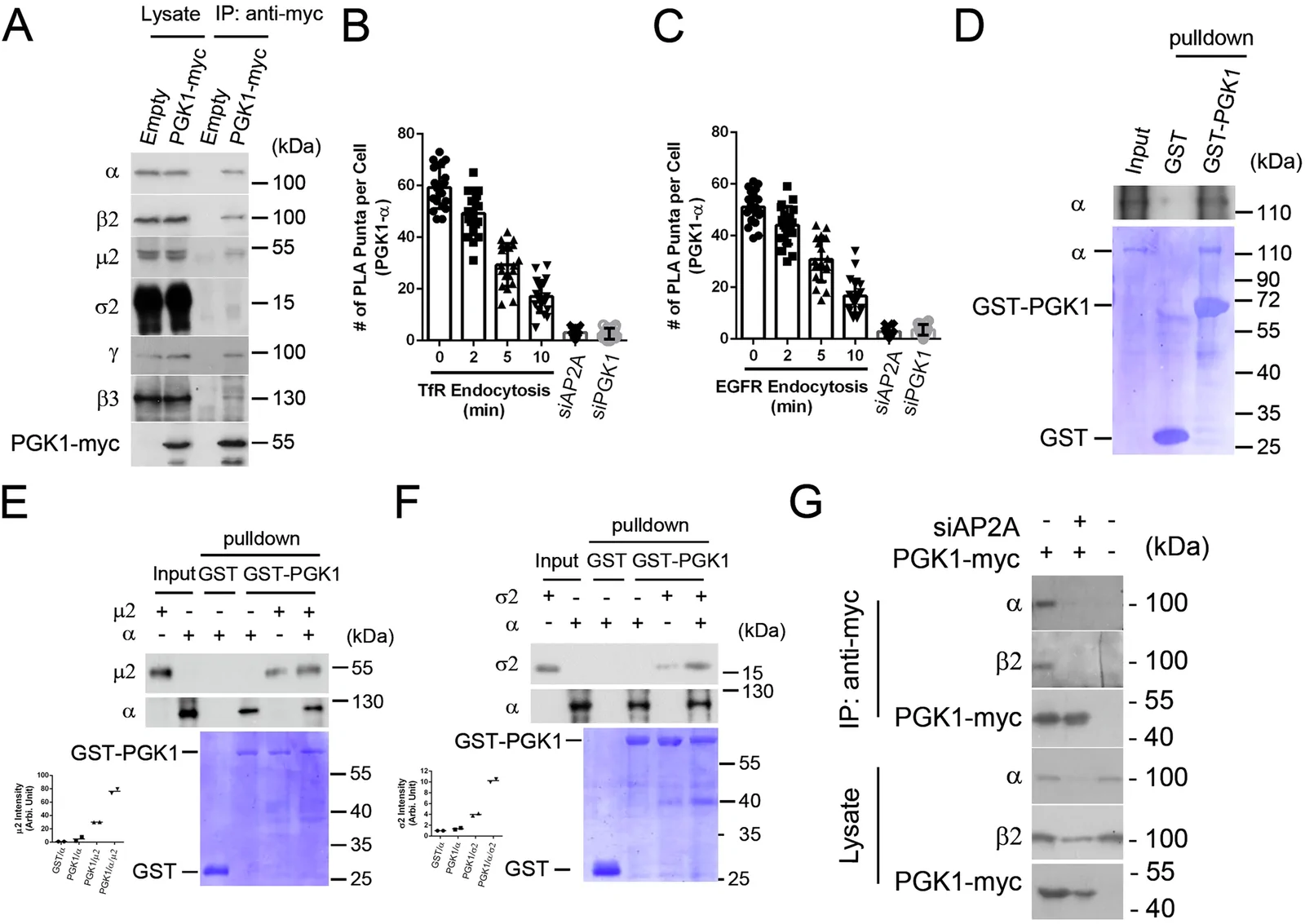
PGK1 interacts with the AP2 complex. Data are shown as the means ± SDs. (**A**) Coimmunoprecipitation analysis examining the interaction PGK1-myc with endogenous AP2 complex (α, β2, μ2, σ2), AP1 complex (γ), and AP3 complex (β3) (two independent biological replicates). (**B**) PLA analysis examining the interaction between PGK1 and α during TfR endocytosis (*n* = 20 cells of one representative experiment from independent two biological replicates). (**C**) PLA analysis examining the interaction between PGK1 and α during EGFR endocytosis (*n* = 20 cells of one representative experiment from independent two biological replicates). (**D**) Pull-down assay examining the interaction of GST-PGK1 with purified α (three independent biological replicates). (**E**) Pull-down assay examining the interaction of GST-PGK1 with purified α and μ2. The quantification shows relative μ2 intensity (two independent biological replicates). (**F**) Pull-down assay examining the interaction of GST-PGK1 with purified α and σ2. The quantification shows relative σ2 intensity (two independent biological replicates). (**G**) Coimmunoprecipitation analysis examining the effect of siRNA against AP2A on the association of PGK1-myc with endogenous β2 in HeLa cells (three independent biological replicates). Source data are available online for this figure.

Our initial proteomic analysis revealed that PGK1 associated with all four subunits of the AP2 complex, supporting its interaction with the intact heterotetramer complex rather than with an individual subunit. We next investigated the association of PGK1 with the AP2 complex and, consistently, found that PGK1 associated with α, β2, μ2, and σ2 of AP2 complex (Fig. 2A). We also noted that PGK1 interacted with γ subunit of AP1 and β3 of AP3 complex, indicating the possibility that PGK1 might also contribute to additional adaptor-mediated transport pathways. A proximity ligation assay (PLA) also demonstrated the interaction of PGK1 and α in intact cells (Fig. 2B,C). Interestingly, the PGK1 and α interaction was largely detected during TfR and EGFR endocytosis and peaked at the initial stage after the induction of endocytosis (Fig. 2B,C; Appendix Fig. S2A,B). These data suggest that PGK1 associates with the AP2 complex upon the initiation of endocytosis. We next investigated the possibility of a direct interaction between PGK1 and the AP2 complex and found that recombinant PGK1 and α subunit directly interact with one another (Fig. 2D). We next postulated that α can serve as a bridge to link PGK1 with other subunits of the AP2 complex. Although PGK1 weakly interacted with the μ2 and σ2, in the presence of α, the binding of μ2 and σ2 to PGK1 was noticeably stronger (Fig. 2E,F). This enhancement was not observed in the presence of β2 subunit (Appendix Fig. S2C,D), suggesting that α subunit serves as a linker in the binding of PGK1 and the AP2 complex. This interaction was further confirmed in cells by showing the reduction in PGK1 interactions with the β subunit in siAP2A-treated cells (Fig. 2G). However, depletion of PGK1 did not disrupt the overall assembly of the AP2 complex (Appendix Fig. S2E), while knockdown of AP2M led to loss of the association between PGK1 and the α subunit, which is likely due to destabilization of the AP2 complex upon μ2 depletion (Appendix Fig. S2F). Collectively, these data suggest that PGK1 associates with the intact AP2 complex during the early stage of endocytosis.

**Figure 3:**
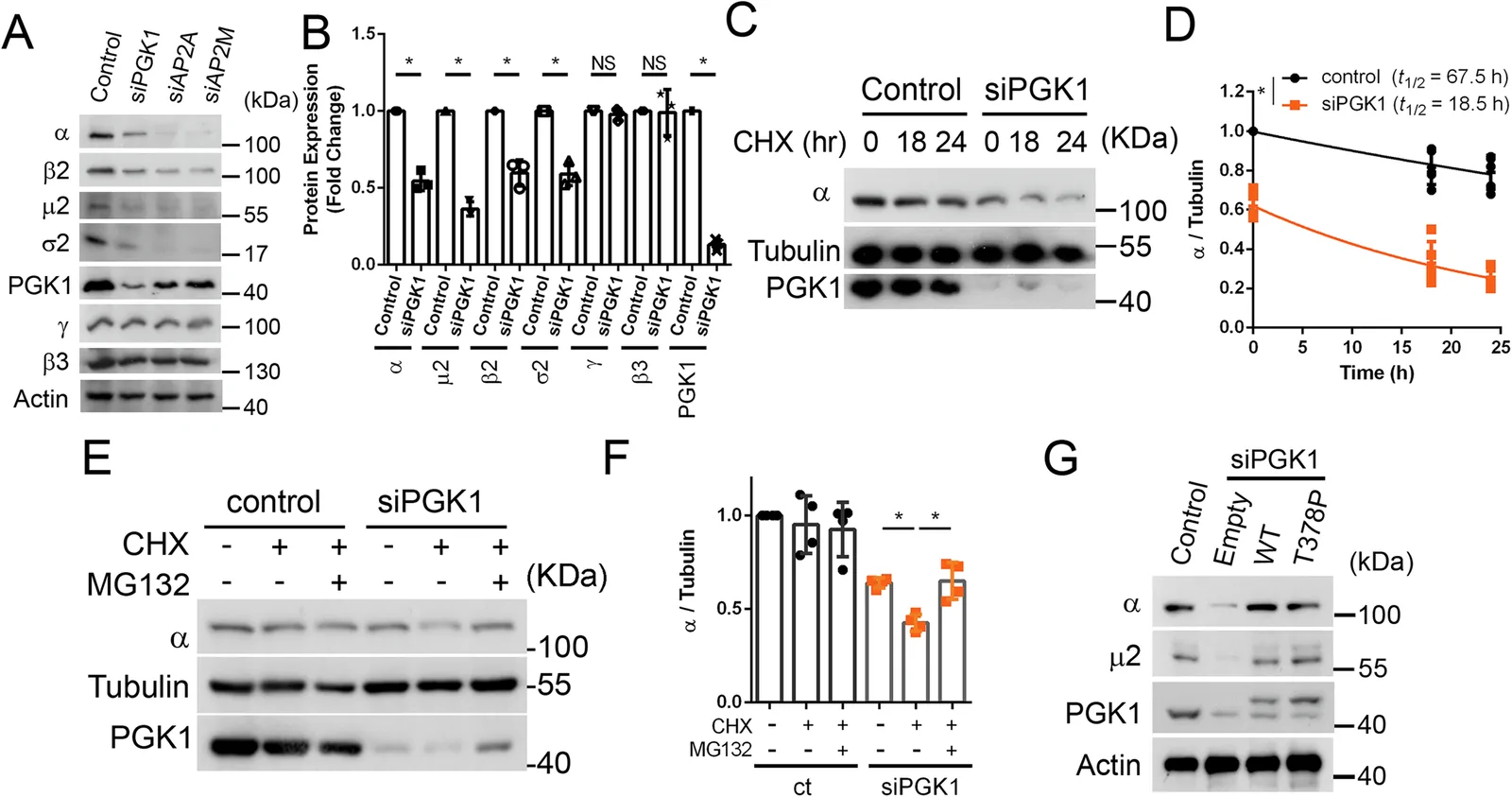
PGK1 stabilizes the AP2 complex. Data are presented as the means ± SDs. *P < 0.05 NS *P* > 0.05. (**A**) Immunoblot analysis of HeLa cell lysates to examine the effects of treatment with siRNAs targeting PGK1, AP2A, or AP2M. (**B**) Quantitation of the indicated protein expression levels relative to the control, normalized to Actin levels (three independent biological replicates). Unpaired two-sided Student’s *t* test is shown for α (*P* = 0.0002), β2 (*P* = 1.8 × 10^−5^), μ2 (*P* = 0.0012), σ2 (*P* = 0.0007), γ (*P* = 0.3548), β3 (*P* = 0.9173), and PGK1 (*P* = 2.3 × 10^−6^). (**C**) Time course analysis of α degradation upon CHX treatment for the indicated durations. (**D**) Protein levels of α were normalized to Tubulin and plotted against time (five independent biological replicates). Curves were fitted using a one-phase decay non-linear regression model. The calculated half-life (*t*_1/2_) is 67.5 h for the control and 18.5 h for siPGK1. Curves were compared using an extra sum-of-squares *F* test (*P* = 6.3 × 10^−6^). (**E**) α degradation upon CHX treatment combined with MG132 treatment in control and siPGK1-treated cells. (**F**) Quantification of the α protein level normalized to the Tubulin level (five independent biological replicates). Two-way ANOVA with Tukey’s test for siPGK1 cells: vehicle control versus CHX (*P* = 0.0036); CHX versus MG132 (*P* = 0.0028). (**G**) Immunoblot analysis of HeLa cell lysates to examine the effects of siPGK1, PGK1 and PGK1^T378P^ (three independent biological replicates). Source data are available online for this figure.

Having demonstrated that PGK1 directly interacts with the AP2 complex, we next analysed the consequences of altering PGK1 gene expression on the protein levels and functions of the AP2 complex. We first found that the expression of the α, β2, μ2, and σ2 subunits, but not γ of AP1 complex and β3 of AP3 complex, was largely diminished in siPGK1-treated cells (Fig. 3A,B). We also confirmed that the gene expression levels of AP2Α and AP2M were not significantly affected in siPGK1-treated cells (Appendix Fig. S3A). Our findings suggested that PGK1 is specifically required to maintain the expression of the AP2 complex components. We next used a cycloheximide (CHX) chase assay to determine whether PGK1 knockdown influences the degradation of the endogenous AP2 complex. Compared with that in control cells, the half-life (*t*_1/2_) of α subunit in PGK1-knockdown cells was significantly shortened in the CHX chase assay (Fig. 3C,D). In addition, the degradation of α subunit was blocked by treatment with the proteasome inhibitor MG132 (Fig. 3E,F). The ubiquitination level of α was further enhanced in siPGK1-treated cell (Appendix Fig. S3B). These data suggested that the lower levels of the AP2 complex in PGK1 knockdown cells are due to proteasomal degradation. We further found that the expression of the kinase-dead mutant PGK1-T378P, like wild-type PGK1, can restore the degradation of α in siPGK1-treated cells (Fig. 3G; Appendix Fig. S3C,D). Collectively, these results demonstrate that PGK1 regulates the stabilization of the AP2 complex independent of its catalytic activity.

### PGK1 acts as a cargo adaptor to promote AP2-dependent endocytosis

**Figure 4:**
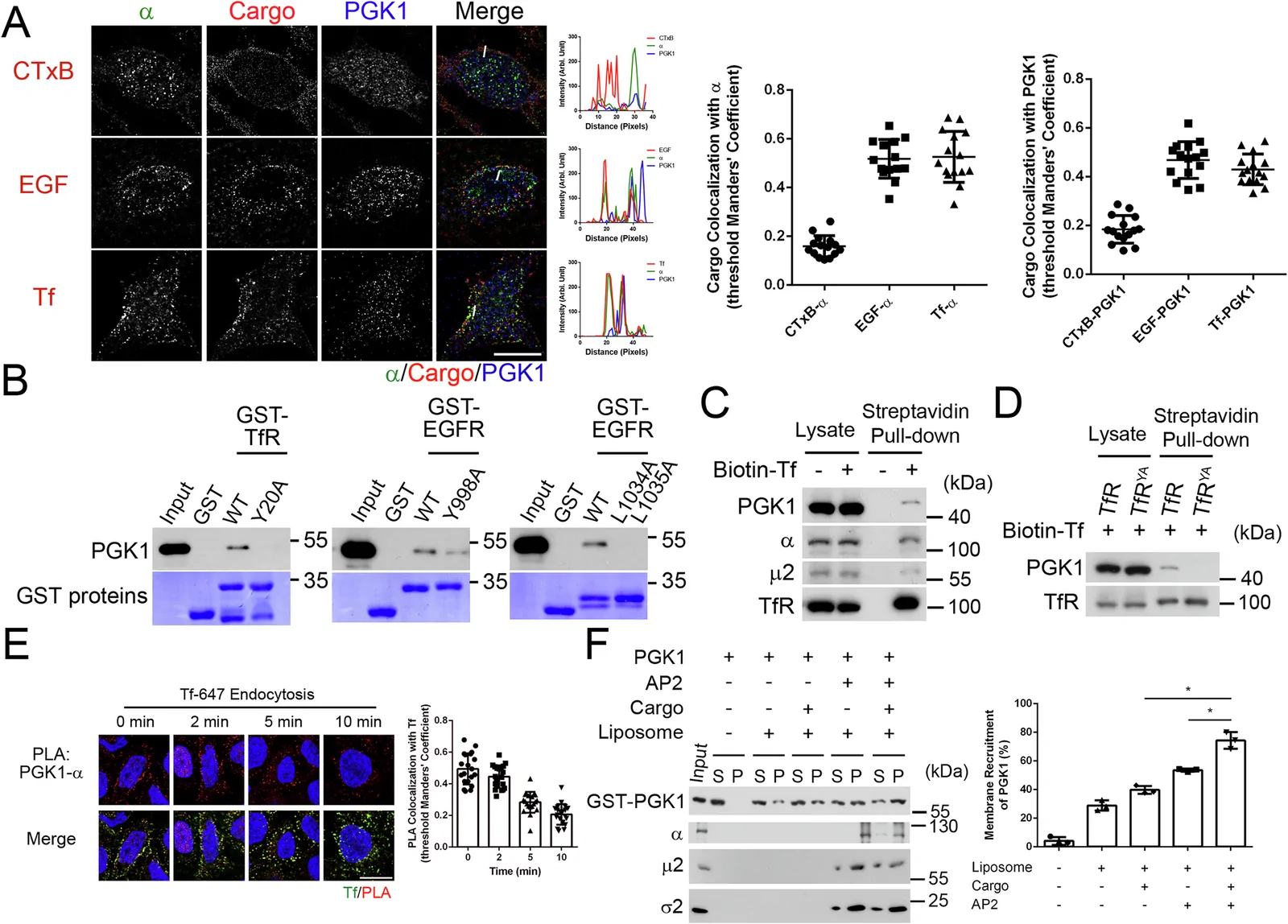
PGK1 associates with cargoes to regulate protein endocytosis. Data are shown as the means ± SDs. *P < 0.05. (**A**) Immunostaining after pre-lysis extraction (left; scale bar, 10 μm) of α (green), cargoes (red: CTxB, EGF, or Tf), and PGK1 (blue) at the 2-min time point internalization. Intensity profiles show the relative fluorescence intensities along the white lines in the merge panels. Quantification (right) of colocalization between cargoes and α or between cargoes and PGK1 expressed as thresholded Manders’ coefficients (*n* = 15 cells of one representative experiment from independent two biological replicates). (**B**) In vitro binding analysis of His-PGK1 and different GST-cargo fragments via GST pull-down to analyse the binding effect with different cargo fragments (three independent biological replicates). (**C**) Endogenous surface TfRs associated with endogenous PGK1 and the AP2 complex in vivo. Surface TfRs were bound by biotin-labeled Tf, isolated with streptavidin beads, and immunoblotted for the indicated proteins (three independent biological replicates). (**D**) Myc-tagged TfR, but not TfR-YA, associated with endogenous PGK1 (two independent biological replicates). (**E**) PLA analysis showing colocalization of PGK1-α PLA puncta with Tf during TfR endocytosis (*n* = 20 cells of one representative experiment from independent two biological replicates); scale bar, 10  μm. (**F**) Reconstitution of PGK1 recruitment to liposomes in the presence of cargo and the AP2 complex. Representative immunoblot (left) and quantification (right) PGK1 membrane recruitment (three independent biological replicates). One-way ANOVA with Dunnett’s test relative to the presence of both the cargo and the AP2 complex: cargo only (*P* = 0.0286) and AP2 complex only (*P* = 0.0295). Source data are available online for this figure.

To provide more direct evidence that PGK1 plays a role in protein endocytosis together with the AP2 complex, we used multiple approaches. First, we assessed whether PGK1 and the AP2 complex colocalized with protein cargoes during protein endocytosis. To avoid potential interference from the cytosolic free portion of PGK1 during colocalization analysis, we used a pre-lysis method to eliminate glycolytic PGK1, which is abundant in the cytosol (Hinckelmann et al, 2016). The cells were permeabilized before fixation to sustain only the proteins associated with membranous organelles. We found that both endogenous α subunit and PGK1 colocalized with the cargo proteins TfR and EGFR but not with CTxB (Fig. 4A), which is consistent with our data showing that CTxB uptake does not require PGK1 or the AP2 complex during endocytosis. We then examined whether the localization of one was affected upon silencing the other. We found that the colocalization of endogenous PGK1 and Tf was increased in cells treated with siRNA against AP2A (Appendix Fig. S4A). In cells treated with siPGK1, we also detected an increase in the colocalization of α subunit with Tf (Appendix Fig. S4B). These data suggest that increased PGK1 or α colocalization with cargo proteins upon AP2Α or PGK1 silencing may be the mechanism by which cells maintain protein endocytosis.

Second, since cargo recognition by adaptor proteins requires their direct interaction with cargo proteins, we investigated whether PGK1, like the AP2 complex, also interacts with the cytoplasmic tails of TfR and EGFR. Previous data have shown that the dileucine motif is recognized by the σ2 subunit, whereas the tyrosine-based sorting signal interacts with the μ2 subunit (Collawn et al, 1990; Sorkin et al, 1996; Wang et al, 2007). Interestingly, in our study, PGK1 recognized both dileucine- and tyrosine-based sorting motifs, and those interactions could be specifically inhibited by mutating these sorting motifs using yeast two-hybrid assay (Appendix Fig. S4C) and in vitro binding assay (Fig. 4B). Mutations of the dileucine or tyrosine-based motifs to alanine on the cytoplasmic tail of EGFR and TfR inhibited the binding by PGK1, indicating the specificity of the interaction (Fig. 4B). Significantly, co-incubation of PGK1 and AP2 complex enhanced their direct interactions with cargo peptides (Appendix Fig. S4D). We next determined whether the binding between the PGK1/AP2 complex and surface TfRs can be detected in vivo. We utilized biotin-conjugated Tf bound to the cell surface and observed that the surface TfR bound to the AP2 complex and PGK1 upon endocytosis (Fig. 4C). In addition, the transfected wild-type and kinase-dead mutant forms of PGK1 similarly associated with the surface TfR and α (Appendix Fig. S4E), which supported our previous observation that PGK1 regulates endocytosis in a manner that is independent of its glycolytic activity. Consistently, the association between PGK1, surface EGFR and the AP2 complex was also detected, regardless of PGK1 catalytic activity (Appendix Fig. S4F,G). Moreover, mutations in the tyrosine-based sorting signal of TfR significantly weakened the interaction with PGK1 (Fig. 4D). These observations demonstrate that PGK1, like the AP2 complex, acts as an adaptor protein to recognize sorting signals on cargo proteins to control their endocytosis.

Third, we examined the association of PGK1 and α subunit in situ via PLA and detected the concurrent presence of Tf cargo protein with the location where PGK1 and α association was detected (Fig. 4E). Our data also revealed that the cooccurrence between Tf cargo protein and PGK1-α association peaked at the initial stage after the induction of endocytosis (Fig. 4E). A similar phenomenon was observed when we employed an EGF cargo protein as an alternative approach for EGFR endocytosis (Appendix Fig. S4H). To further investigate whether cargo binding promotes the recruitment of adaptor proteins to the membrane (Kelly et al, 2014), we prepared synthetic PI(4,5)P_2_-containing liposomes alone or liposomes with a lipid-linked tyrosine-based motif. These liposomes were then incubated with recombinant PGK1 and AP2 components. Strikingly, PGK1 exhibited a stronger binding affinity to liposomes containing cargo peptides, and the interaction was further augmented with cargo-containing liposomes preincubated with AP2 components (Fig. 4F), indicating that PGK1 bound to both the AP2 complex and cargo proteins with greater affinity than PGK1 bound solely to PI(4,5)P_2_-containing liposomes. Together, our data suggest that the interaction of PGK1 with the AP2 complex enhances cargo recognition and promotes endocytosis.

### The interaction between PGK1 and α subunit is required to support protein endocytosis

**Figure 5:**
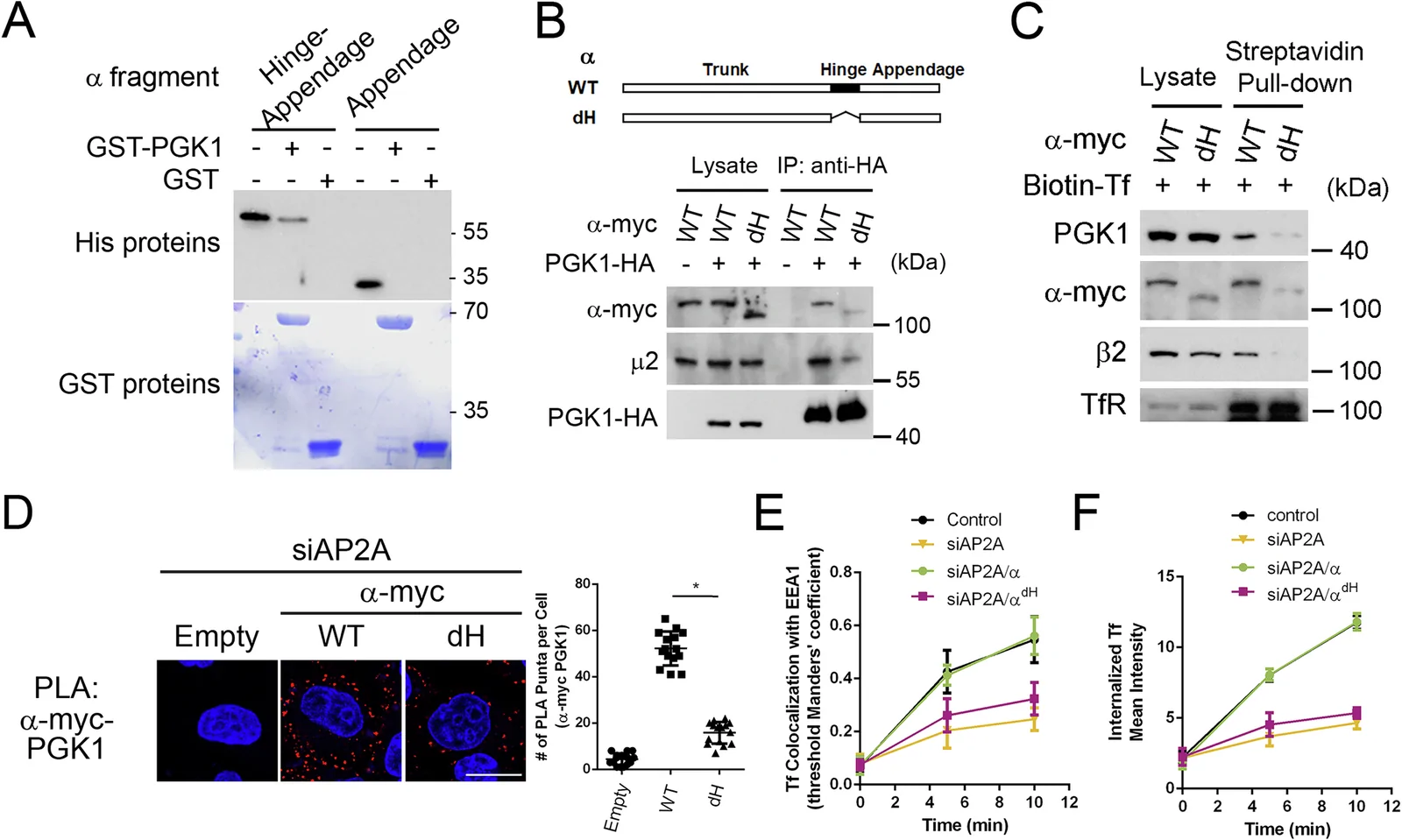
The hinge region of α subunit is required for its interaction with PGK1 and the promotion of protein endocytosis. Data are shown as the means ± SDs. *P < 0.05. (**A**) In vitro binding analysis of purified GST-PGK1 and purified different α fragments via GST pull-down to analyse the binding effect with different α fragments (three independent biological replicates). (**B**) Immunoprecipitation of HA-tagged PGK1 for association with α or α-dH (three independent biological replicates). (**C**) Surface TfRs associated with endogenous PGK1 and myc-tagged α or α-dH in siAP2A-treated cells (three independent biological replicates). (**D**) PLA analysis comparing the interaction of PGK1 with myc-tagged α or α-dH in siAP2A-treated cells (*n* = 15 cells of one representative experiment from independent two biological replicates); scale bar, 10 μm. (**E**) Colocalization of Tf with EEA1, expressed as thresholded Manders’ coefficients, comparing the effects of α with α-dH in siAP2A cells at 0, 5, and 10 min (*n* = 15 cells of one representative experiment from independent two biological replicates). (**F**) Mean intensity of internalized Tf comparing the effects of α with α-dH in siAP2A cells at 0, 5, and 10 min (*n* = 15 cells of one representative experiment from independent two biological replicates). Source data are available online for this figure.

To gain insight into whether the interaction between PGK1 and α subunit is required for protein endocytosis, we analysed the domain of α subunit that directly interacts with PGK1. We first found that the C-terminal hinge-appendage domain was copurified with GST-PGK1 and directly interacted with PGK1, whereas the appendage domain alone significantly prevented the interaction with PGK1 (Fig. 5A; Appendix Fig. S5A), suggesting that the hinge region of α subunit is required for the interaction with PGK1. We then generated α without the hinge region (α-dH) and observed a significant reduction in PGK1 binding in vivo (Fig. 5B; Appendix Fig. S5B). We also confirmed that, like full-length α, α-dH is associated with other AP2 components (Appendix Fig. S5C), indicating that the hinge region is specifically involved in the interaction with PGK1. Moreover, CHX chase assays revealed that, compared with α, a faster decrease in α-dH was observed (Appendix Fig. S5D), indicating that the PGK1 binding stabilizes α subunit. We next determined the association of surface TfR with the AP2 complex and PGK1 upon endocytosis and found that the expression of α-dH reduced the association of both the AP2 complex and PGK1 with TfR (Fig. 5C). PLA further confirmed these effects of α-dH, in which α-dH decreased the interaction with PGK1 in vivo (Fig. 5D). The colocalization of α-dH and PGK1 was also decreased upon TfR and EGFR endocytosis (Appendix Fig. S5E,F). Consistently, α-dH significantly inhibited both TfR and EGFR endocytosis, as indicated by a significant decrease in their internalization and colocalization with early endosomes (Fig. 5E,F; Appendix Fig. S5G–J). Thus, these collective results reveal that the interaction of PGK1 with α subunit is required for cargo recognition to promote protein endocytosis.

**Figure 6:**
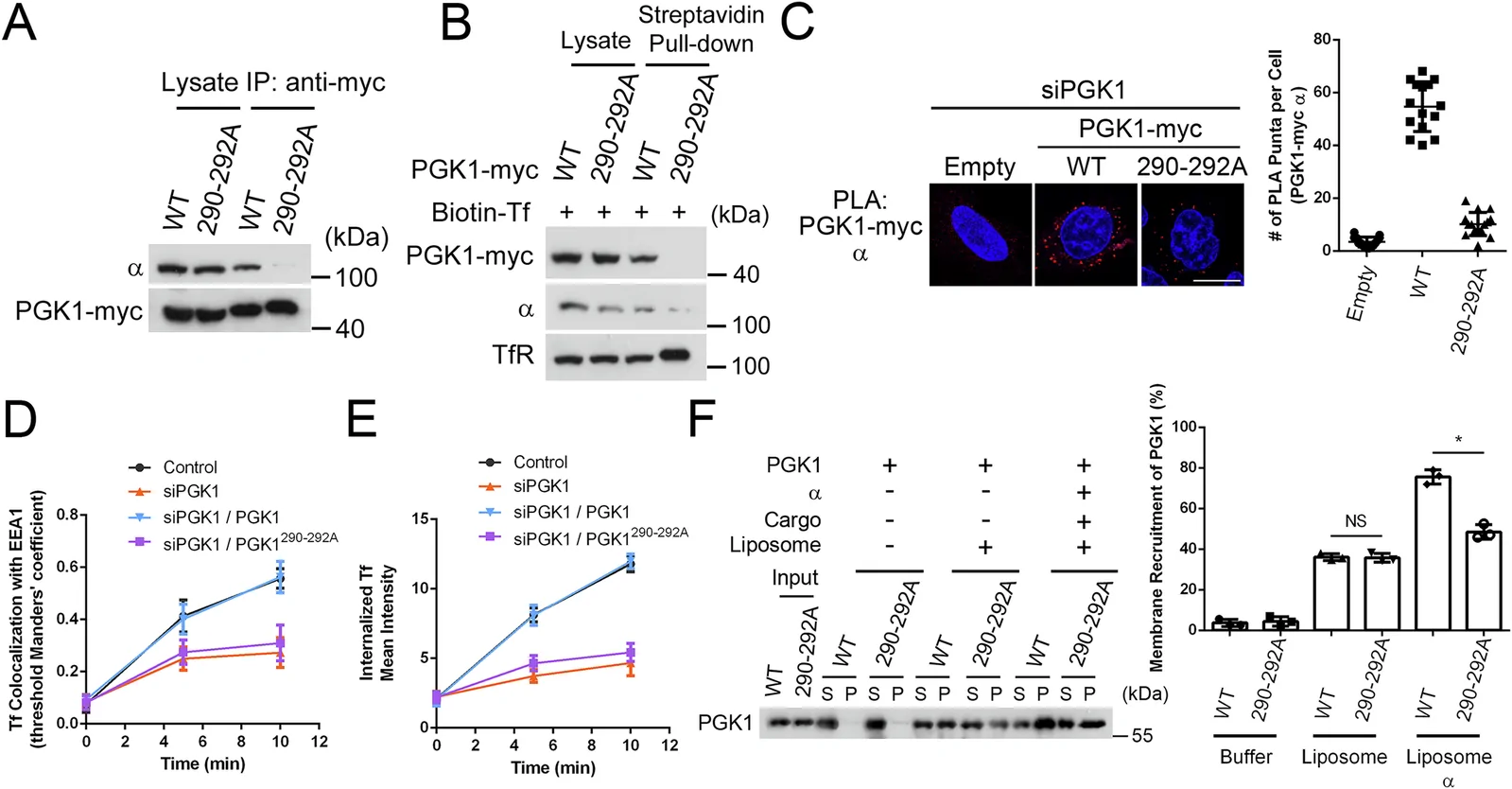
The ^290^DKF^292^ motif of PGK1 is required for its interaction with α subunit and the promotion of protein endocytosis. Data are shown as the means ± SDs. *P < 0.05; NS *P* > 0.05. (**A**) Immunoprecipitation of myc-tagged PGK1 comparing the associations of α with PGK1 and PGK1^290-292A^ (three independent biological replicates). (**B**) Endogenous surface TfRs associated with endogenous α and myc-tagged PGK1 or PGK1^290-292A^ in siPGK1-treated cells (three independent biological replicates). (**C**) PLA analysis comparing the interaction of α with myc-tagged PGK1 and PGK1^290-292A^ in siPGK1-treated cells (*n* = 15 cells of one representative experiment from independent two biological replicates); scale bar, 10 μm. (**D**) Colocalization of Tf with EEA1, expressed as thresholded Manders’ coefficients, comparing the effects of PGK1 and PGK1^290-292A^ in siPGK1 cells at 0, 5, and 10 min (*n* = 15 cells of one representative experiment from independent two biological replicates). (**E**) Mean intensity of internalized Tf comparing PGK1 and PGK1^290-292A^ in siPGK1 cells at 0, 5, and 10 min (*n* = 15 cells of one representative experiment from independent two biological replicates). (**F**) Reconstitution of PGK1 recruitment to liposomes comparing the effects of PGK1 or PGK1^290-292A^. Representative immunoblott (left) and quantification (right) PGK1 membrane recruitment (three independent biological replicates). Unpaired Student’s *t* test for WT vs. 290-292 A: liposome group (*P* = 0.8534); liposome in the presence of cargo and α groups (*P* = 0.0007). Source data are available online for this figure.

We next sought to identify the specific region on PGK1 involved in the α subunit interaction. Previous data suggested that several endocytic accessory proteins are recruited by interactions with α subunit via the DxF motif (Fiuza et al, 2017). Notably, PGK1 contains a ^290^DKF^292^ motif that conforms to these consensus sequences. We then found that mutations in the DKF motif of PGK1 (PGK1^290-292A^) prevented its interaction with α in cells (Fig. 6A; Appendix Fig. S6A). We further found that the expression of PGK1^290-292A^ failed to reverse the degradation of α subunit in siPGK1-treated cells (Appendix Fig. S6B,C). However, we further found that, compared with wild-type PGK1, PGK1^290-292A^ had a weaker association with cargo proteins in vivo (Fig. 6B; Appendix Fig. S6D), suggesting that the interaction of PGK1 with α subunit also promotes its association with cargo proteins. In addition, PLA further confirmed that PGK1^290-292A^ decreased the interaction with α subunit in vivo (Fig. 6C). Furthermore, the expression of PGK1^290-292A^ failed to rescue the endocytosis defect of TfR and EGFR internalization in siPGK1-treated cells (Fig. 6D,E; Appendix Fig. S6E–H). Notably, compared with wild-type PGK1, PGK^290-292A^ had a lower binding affinity for cargo-containing liposomes preincubated with AP2 components (Fig. 6F). Collectively, our data suggest that the interaction between PGK1 and α subunit is required to facilitate efficient protein endocytosis.

### PGK1 is part of the AP2-clathrin coat complex

**Figure 7:**
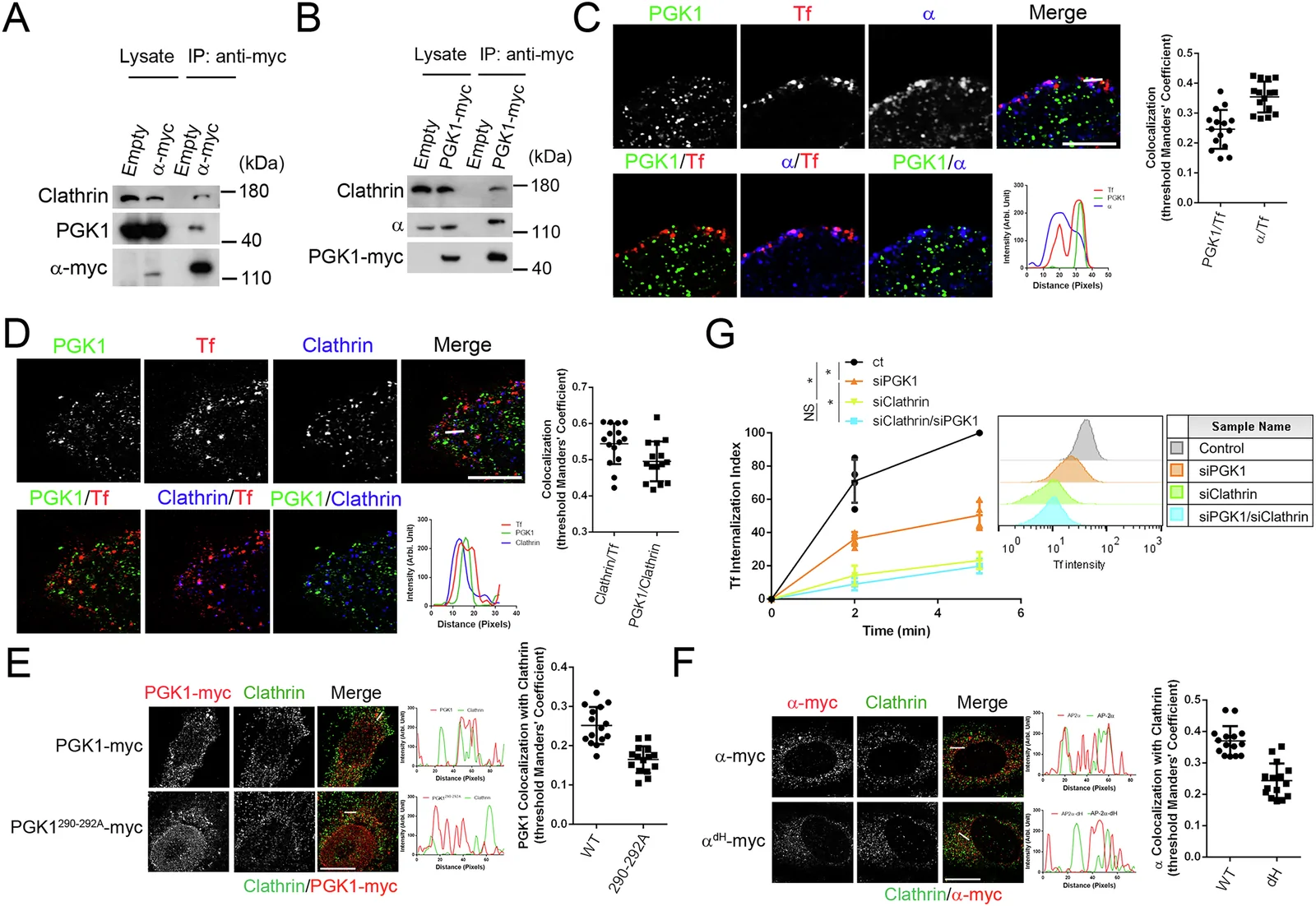
PGK1 is a part of the clathrin-coated complex during protein endocytosis. Data are shown as the means ± SDs. *P < 0.05; NS *P* > 0.05. (**A**) Coimmunoprecipitation analysis examining the association of α-myc with endogenous PGK1 and clathrin (three independent biological replicates). (**B**) Coimmunoprecipitation analysis examining the association of PGK1-myc with endogenous α and clathrin (three independent biological replicates). (**C**) Super-resolution confocal image after pre-lysis extraction showing colocalization of PGK1, α, and Tf after 2 min of internalization; scale bar, 10 μm. Intensity profiles show the relative fluorescence intensities along the white lines in the merge panels. Quantification (right) of PGK1 with Tf and of α with Tf colocalization, expressed as thresholded Manders’ coefficients (*n* = 15 cells of one representative experiment from independent two biological replicates). (**D**) Super-resolution confocal image after pre-lysis extraction showing colocalization of PGK1, clathrin, and Tf after 2 min of internalization; scale bar, 10 μm. Intensity profiles show the relative fluorescence intensities along the white lines in the merge panels. Quantification (right) of PGK1 with clathrin and clathrin with Tf colocalization, expressed as thresholded Manders’ coefficients (*n* = 15 cells of one representative experiment from independent two biological replicates). (**E**) Colocalization analysis after pre-lysis extraction demonstrating the effect of clathrin and PGK1 or PGK1^290-292A^ colocalization upon 2 min of internalization. Image (left; scale bar, 10 μm), intensity profiles (middle) show the relative fluorescence intensities along the white lines in the merge panels, and quantification (right) show the PGK1-myc colocalization with clathrin, expressed as thresholded Manders’ coefficients (*n *= 15 cells of one representative experiment from independent two biological replicates). (**F**) Colocalization analysis after pre-lysis extraction demonstrating the effect of clathrin and α or α^dH^ colocalization upon 2 min of internalization. Image (left; scale bar, 10  μm), intensity profiles (middle) show the relative fluorescence intensities along the white lines in the merge panels, and quantification (right) show the α-myc colocalization with clathrin, expressed as thresholded Manders’ coefficients (*n* = 15 cells of one representative experiment from independent two biological replicates). (**G**) Internalization of Tf quantified by flow cytometry at 0, 2, and 5 min (*n* > 10,000 cells, four independent biological replicates). One-way ANOVA with Tukey’s test at the 5-min time point: control vs. siPGK1 (*P* = 0.0034); control vs. siClathrin (*P* = 0.0002); siPGK1 vs. siClathrin/siPGK1 (*P* = 0.0277); siClathrin vs. siClathrin/siPGK1 (*P* = 0.5985). Source data are available online for this figure.

As further evidence in favour of the role of PGK1 working together with AP2 complex to promote endocytosis, we examined whether the clathrin coat complex contains PGK1 as a coat component. We initially confirmed that α subunit associated with PGK1 and clathrin in cells (Fig. 7A), and the interaction of PGK1 with α and clathrin was also detected via a coprecipitation experiment (Fig. 7B). We next used super-resolution confocal microscopy to observe the intracellular vesicular structure and found that a significant portion of PGK1 and α subunit colocalized with the cargo proteins Tf and EGF (Fig. 7C; Appendix Fig. S7A). In addition, we observed the structure of clathrin with PGK1 and the cargo proteins Tf and EGF (Fig. 7D; Appendix Fig. S7B). We further found that wild-type PGK1 colocalized with clathrin, whereas the α-interacting defect mutant PGK1^290-292A^ significantly decreased the degree of colocalization (Fig. 7E). Additionally, we observed the colocalization of α subunit with clathrin, whereas the binding of the PGK1-interacting defect mutant α-dH to clathrin was noticeably reduced (Fig. 7F). By combining super-resolution confocal microscopy with z-stack acquisition, we detected robust colocalization of the cargo proteins Tf and EGF with the PGK1-α complex in cells (Appendix Fig. S7C,D). We further observed the concurrent presence of clathrin with the location where PGK1 and α association was detected by PLA signals (Appendix Fig. S7E). Moreover, compared with the decrease in TfR endocytosis in siClathrin-treated cells, TfR endocytosis was not further decreased in clathrin and PGK1 co-knockdown cells (Fig. 7G), suggesting that although the PGK1 and AP2 complex form co-adaptor for cargo recognition, clathrin triskelia act as the common outer coat for PGK1-mediated endocytosis. Together, these data suggest the presence of a PGK1-containing AP2-clathrin coat complex for protein endocytosis.

**Figure 8:**
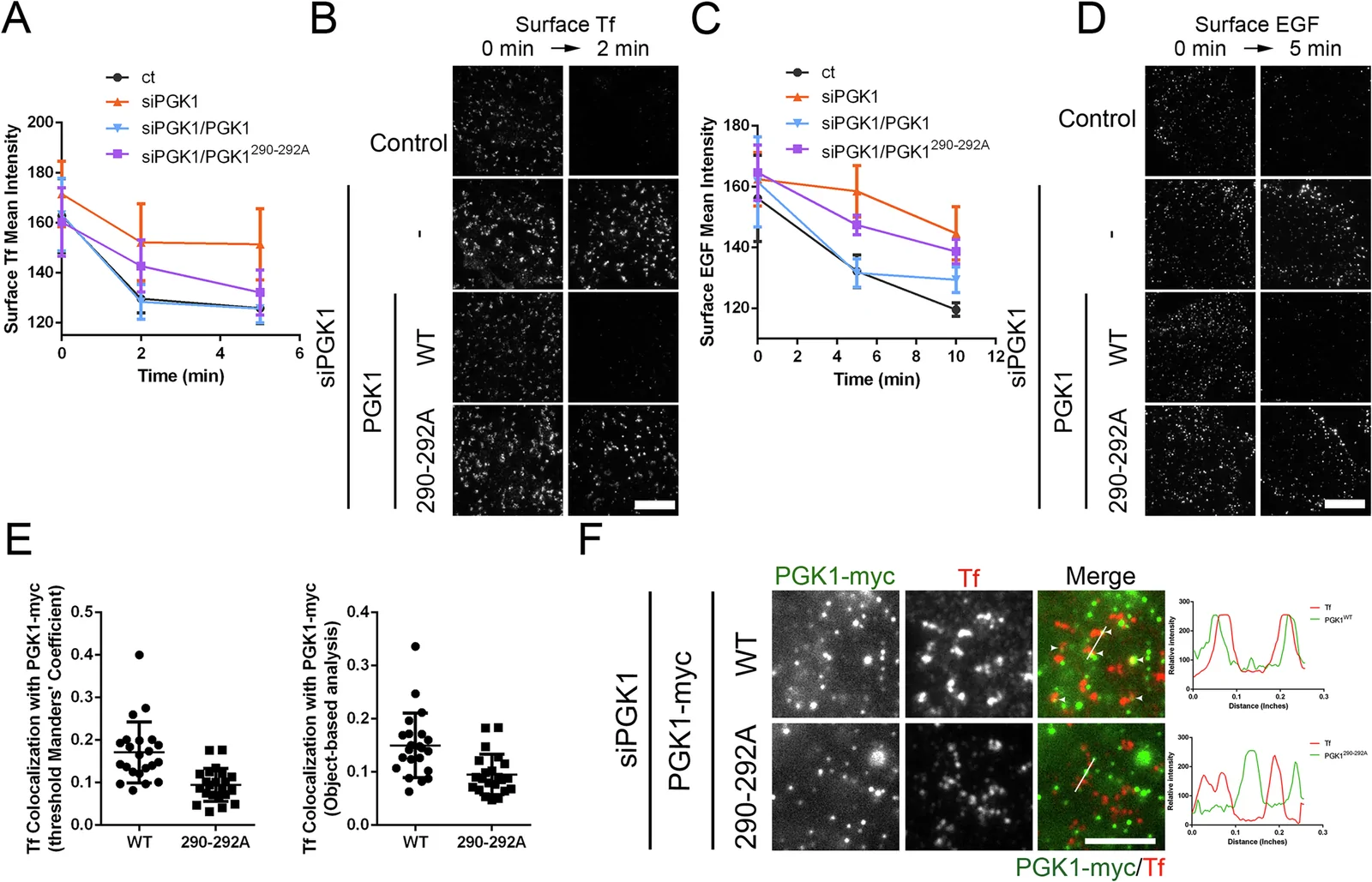
TIRF imaging demonstrated that PGK1 acts as a co-adaptor to recognize cargoes at the plasma membrane. Data are shown as the means ± SDs. (**A**) Mean intensity of surface Tf comparing the effects of PGK1 and PGK1^290-292A^ in siPGK1-cells at 0, 2, and 5 min (*n* = 20 cells of one representative experiment from independent two biological replicates). (**B**) TIRF imaging representing the surface Tf; scale bar, 5 μm. (**C**) Mean intensity of surface EGF comparing the effects of PGK1 expression with PGK1^290-292A^ expression in siPGK1-cells at 0, 5, and 10 min (*n* = 20 cells of one representative experiment from independent two biological replicates). (**D**) TIRF imaging representing the surface EGF; scale bar, 5 μm. (**E**) Quantitative results of Tf colocalization with PGK1-myc, expressed as thresholded Manders’ coefficient (left) and as object-based analysis (right) (*n* = 22 cells of one representative experiment from independent two biological replicates). (**F**) TIRF imaging representing the colocalization of Tf and PGK1-myc; scale bar, 5 μm. Arrows indicate the colocalization. Intensity profiles show the relative fluorescence intensities along the white lines in the merge panels. Source data are available online for this figure.

To directly visualize endocytic events at the plasma membrane, we employed total internal reflection fluorescence (TIRF) microscopy which selectively detects fluorescence signals in close proximity to the plasma membrane. We first observed elevated Tf and EGF signals on the plasma membrane in cells treated with siRNA against PGK1 (Fig. 8A–D). In siPGK1-treated cells, expression of the PGK1^290-292A^ mutant, but not wild-type PGK1, significantly failed to restore Tf and EGF internalization (Fig. 8A–D). Consistently, we found a significant abundance of PGK1, comparable to α, in Tf-positive structures using TIRF (Appendix Fig. S8A,B), whereas this enrichment was absent in cells expressing PGK1^290-292A^ mutant (Fig. 8E,F). Together, these results indicate that PGK1 functions as a co-adaptor that cooperates with AP2 complex to ensure proper cargo recognition and facilitate protein endocytosis.

## Discussion

We discovered that PGK1 functions together with AP2 complex to drive efficient protein endocytosis. Elucidating this role, we initially reported that simultaneous knockdown of PGK1 and the AP2 complex synergistically blocks protein endocytosis. We then revealed that the catalytic activity of PGK1 is not required for its regulation of endocytosis. We also noted that PGK1 directly interacts with α subunit, stabilizing the AP2 complex. Remarkably, unlike the AP2 complex, in which σ2 and µ2 recognize the dileucine motif and the tyrosine-based motif, respectively, PGK1 itself can directly bind to both to promote endocytosis. The association of PGK1 and the AP2 complex also facilitates the cargo recognition process. These observations suggest that PGK1 acts simultaneously as an interacting partner with α subunit for the stabilization of AP2 complex and as a cargo adaptor for cargo recognition to support AP2-dependent endocytosis. Notably, PGK1 is a key auxiliary coat component and part of the clathrin-coated complex that mediates protein endocytosis.

Our findings advance the understanding of AP2-dependent endocytosis in multiple ways. First, the canonical tyrosine-based sorting motif and dileucine sorting signal are essential for internalization (Motley et al, 2006). The AP2 complex is considered an endocytic cargo adaptor required for the internalization of certain cargo receptors, such as TfR and EGFR. Although these interactions are specific, the binding affinity needs to be increased to guarantee the proper cargo sorting step. In our case, we identified PGK1 as a cargo adaptor that works together with the AP2 complex to recognize both sorting signals, which tends to increase the molecular avidity. Second, in addition to being an alternative endocytic accessory adaptor (Conner and Schmid, 2003; Motley et al, 2003; Traub, 2003), PGK1 not only binds to lipids and clathrin but also associates with α subunit to stabilize the AP2 complex from being degraded. The dual functions, interactions with lipids and coat proteins and protective effects of PGK1 demonstrate the reciprocal role involved in protein endocytosis. Taken together, our findings suggest that PGK1 promotes TfR and EGFR endocytosis through the optimization of cargo recognition by the AP2 complex.

Our results support a model in which PGK1 associates with the AP2 complex primarily through interaction with the α subunit. Although PGK1 interacts with μ2 and σ2 subunits in vitro, subsequent validation experiments identify α subunit as the dominant interaction partner, suggesting that PGK1 engages the assembled AP2 complex through α subunit as the bridge scaffold. Domain-mapping analyses indicate that the hinge region of α subunit is required for PGK1 association. Interestingly, PGK1 contains a DxF motif that is typically known to interact with the α appendage domain (Praefcke et al, 2004). However, DKF motif of PGK1 is not a canonical DxF motif for the interaction with ear domain. One possible explanation is that structural coupling between the hinge and appendage regions within the intact AP2 complex influences the accessibility or positioning of the appendage domain. The α hinge region is a proline-rich and highly flexible region that connects the AP2 core to the appendage domain and is known to facilitate the recruitment of endocytic accessory proteins by increasing the conformational switch of the appendage domain (Owen et al, 1999). Therefore, the requirement of the hinge region identified in our mapping experiments may not necessarily reflect direct recognition of the binding site within the hinge region itself. Instead, the hinge region may contribute indirectly by positioning the appendage domain for the efficient engagement with PGK1 or by providing the conformational flexibility required for their interaction. In this context, deletion of the hinge region could impair PGK1 association by restricting accessibility of the appendage domain or disrupting their dynamic architecture. Given that AP2 complex undergoes substantial structural rearrangements during membrane recruitment and cargo engagement for the conformational flexibility, accessibility, and spatial coordination of the adjacent appendage domain (Collins et al, 2002; Kovtun et al, 2020), future structural studies under membranous conditions will be required to define the precise interaction interface and to determine how PGK1 association with the AP2 complex may position PGK1 relative to AP2 subunits.

Previous studies have shown that binding of PI(4,5)P_2_ promotes the open conformation of the AP2 complex, which is required for productive clathrin recruitment and cargo engagement (Kelly et al, 2014). In this context, it is notable that deletion of the hinge region of α subunit still reduced the interaction between AP2 and clathrin despite retention of the appendage domain. One possible explanation is that the hinge contributes to the structural coupling and conformational flexibility between the core and appendage domains of α subunit. Loss of this region may alter the spatial orientation of the appendage domain or impair stabilization of the open conformation required for efficient clathrin binding. In addition, our liposome-based assays indicate that PI(4,5)P₂ enhances the interaction between AP2 and PGK1, suggesting that membrane-associated activation of AP2 may facilitate PGK1 recruitment. These observations raise the possibility that PGK1 preferentially associates with the membrane-bound, open conformation of AP2, in which lipid binding, cargo engagement, and adaptor assembly cooperate together to promote efficient endocytic complex formation.

The AP2 complex facilitates endocytosis through two different stages. The initial step is to recognize PI(4,5)P_2_-containing lipids at the plasma membrane. The latter step involves a conformational change in AP2 adaptors so that the cargo binding sites can be exposed for cargo recognition (Jackson et al, 2010; Partlow et al, 2022). The data collected in this study suggest that PGK1 can be recruited to PI(4,5)P_2_-containing liposomes and, notably, that both cargo peptides and the AP2 complex further increase membrane recruitment. As such, the coincident detection of PI(4,5)P_2_ and cargo sorting motifs allows simultaneous recognition by the PGK1-AP2 complex for the proper recognition step during endocytosis.

The endocytic pathway is a dynamic system responsible for lysosomal transport and membrane recycling after being internalized. Once a cargo protein is internalized from the plasma membrane, it is sorted in the early endosome. PGK1 can participate in endocytic transport through two distinct mechanisms: one is for endocytosis via the interaction with AP2 complex revealed from this study, and the other is to determine lysosomal transport of EGFR from our previous findings (Chu et al, 2024). Previous data suggested that AP2 complex is not strictly required for EGFR degradation but plays an important role in recycling (Pascolutti et al, 2019). Our model indicates that PGK1 may coordinate distinct stages of the endocytic pathway, participating in both cargo internalization and lysosomal transport. Thus, determining how cargo adaptors are tightly regulated and coordinated when one cargo protein is internalized and sorted to form different populations of transport vesicles can be more intriguing than appreciated at present.

## Methods

**Table:** Reagents and tools table

Reagent/resource	Reference or source	Identifier or catalog number
**Experimental models**		
HeLa cells	ATCC	CCL-2
HEK293T cells	ATCC	CRL-3216
ExpiSf9™ insect cells	Gibco	A35243
Yeast strain YEM1α (*MATα*, *trp1*, *his3*, *leu2*, *6ops*-*LEU2*, *2ops*-*LacZ*)	Laboratory stock	N/A
*Escherichia coli* BL21	Laboratory stock	
**Recombinant DNA**		
pcDNA3.1-myc-His(-)-PGK1 (WT/T378P)	Chu et al, 2024	Backbone: Invitrogen
pcDNA3.1-myc-His(-)-PGK1 (290-292A)	This study	Backbone: Invitrogen
pEGFP-C3-PGK1 (WT)	Chu et al, 2024	Backbone: Clontech
pcDNA3.1-myc-His(-)-PGK1-HA	This study	Backbone: Invitrogen
pcDNA3.1-myc-His(-)-mCherry-PGK1	This study	Backbone: Invitrogen
pCMV6-AN-mRFP-PGK1	This study	Backbone: OriGene
pGEX-4T-3-PGK1 (WT)	Chu et al, 2024	Backbone: Amersham
pGEX-4T-3-PGK1 (290-292 A)	This study	Backbone: Amersham
pJG4-5-PGK1 (WT)	This study	N/A
pcDNA3.1-myc-His(-)-AP2A (WT/dH)	This study	Backbone: Invitrogen
pIEx-6-AP2A	This study	Backbone: Novagen
pEG202-AP2A	This study	Backbone: NovoPro
pcDNA3.1-myc-His(-)-AP2A-mCherry	This study	Backbone: Invitrogen
GFPspark-CTLB	Sino Biological	Cat# HG20112-ACG
pET32a-AP2M	This study	Backbone: Invitrogen
pET32a-AP2S	This study	Backbone: Invitrogen
pET32a-AP2A (625-941/702-941)	This study	Backbone: Invitrogen
pET30a-AP2A (1-624/625-941/702-941)	This study	Backbone: Invitrogen
pGEX-4T-3-TfR1-61 (WT/Y20A)	This study	N/A
pGEX-4T-3-EGFR 980-1017/1013-1054)	This study	N/A
pEG202-EGFR (992-1005 or 1018-1045)	This study	N/A
pcDNA3.1-myc-His(-)-PGK1 (WT/T378P/290-292 A)	This study	Backbone: Invitrogen
pEGFP-C3-PGK1 (WT/T378P/290-292 A)	This study	Backbone: Clontech
**Antibodies**		
Anti-EEA1	Cell Signaling Technology	2411
Anti-Myc	Cell Signaling Technology	2276
Anti-6×His	Santa Cruz Biotechnology	sc-803
Anti-HA	Santa Cruz Biotechnology	sc-7392
Anti-PGK1	Santa Cruz Biotechnology	sc-130335
Anti-PGK1	GeneTex	GTX107614
Anti-AP2A	BD Transduction Laboratories	610502
Anti-AP2A	Cell Signaling Technology	55521
Anti-AP2M	BD Transduction Laboratories	611351
Anti-AP2B	Santa Cruz Biotechnology	sc-74423
Anti-AP2S	OriGene	TA373563
Anti-AP2S	Abcam	ab128950
Anti-AP1G	Cell Signaling Technology	44676
Anti-AP3B	Cell Signaling Technology	95765
Anti-Clathrin heavy chain	Cell Signaling Technology	2410
Anti-a-Tubulin	Cell Signaling Technology	3873
Anti-Actin	Cell Signaling Technology	4970
Anti-EGFR	Cell Signaling Technology	4267
Anti-TfR	Santa Cruz Biotechnology	sc-65882
HRP goat anti-mouse IgG	Merck Millipore	AP124P
HRP goat anti-rabbit IgG	Merck Millipore	AP132P
Alexa Fluor® 488 goat anti-mouse	Jackson ImmunoResearch	115-545-062
Alexa Fluor® 594 goat anti-mouse	Jackson ImmunoResearch	115-585-062
Alexa Fluor® 647 goat anti-mouse	Jackson ImmunoResearch	115-605-062
Alexa Fluor® 488 donkey anti-rabbit	Jackson ImmunoResearch	711-545-152
Alexa Fluor 594 donkey anti-rabbit	Jackson ImmunoResearch	711-585-152
Alexa Fluor 647 donkey anti-rabbit	Jackson ImmunoResearch	711-605-152
Anti-c-Myc agarose conjugate	Sigma	A7470
Anti-HA agarose conjugate	Sigma	A2095
Duolink® in situ PLA® probe anti-rabbit PLUS	Merck Millipore	Duolink® DUO92002
Duolink® in situ PLA® probe anti-mouse MINUS	Merck Millipore	DUO92004
**Oligonucleotides and other sequence-based reagents**		
siPGK1 (5’-GCUUCUGGGAACAAGGUUA-3’)	Dharmacon	N/A
siAP2A (5’-AGAGCAUGUGCACGCUGGC-3’)	Dharmacon	N/A
siAP2M (5’-AGUGGAUGCCUUUCGGGUC-3’)	Dharmacon	N/A
siClathrin (5’- AAUUGGUCUUCGAAUUGGA-3’)	Dharmacon	N/A
ACTIN qPCR primers (forwards: 5’-TGATCTTCATTGTGCTGGGTG-3’; reverse: 5’-ACATCCGCAAAGACCTGTAC-3’)	This study	N/A
AP2A qPCR primers (forwards: 5’- CGGAACTGTAAGAGCAAAGAGG’; reverse: 5’-CCAAGCAGGAAGATGAAAAGC-3’)	This study	N/A
AP2M qPCR primers (forwards: 5’- CAAGCAAGAGCGGGAAGC’; reverse: 5’- CCATCTGGCGGGATAAAGC -3’)	This study	N/A
**Chemicals, enzymes and other reagents**		
DMEM with high glucose	Cytiva	SH30022.02
FBS	Corning™	35010CV
penicillin‒streptomycin solution (PS)	Cytiva	SV30010
ExpiSf™ CD medium	Gibco™	A3767801
Polyethylenimine, linear, MW 25000 (PEI 25 K™)	Polysciences	23966
Alexa Fluor™ 488/555/647-conjugated transferrin	Invitrogen	T13342/T35352/T23366
Alexa Fluor™ 488/555/647-conjugated EGF	Invitrogen	E13345/E35350/E35351
Alexa Fluor™ 555-conjugated CTxB	Invitrogen	C34776
Dynasore	Sigma	D7693
Cycloheximide (CHX)	Sigma	C1988
MG132	Cell Signaling Technology	2194
Glutathione Sepharose 4B	GE Healthcare	17075605
Nickel affinity resin	Thermo Fisher	88222
Spectra/Por 4 Dialysis Tubing, 12–14 kDa MWCO	Repligen	132700
Fluoromount-G™ Mounting Medium with DAPI	Invitrogen™	00-4959-52
Streptavidin agarose	Merck Millipore	16126
DOPC	Avanti® Polar Lipids	850375 C
DOPE	Avanti® Polar Lipids	850725 C
PI(4,5)P2	Avanti® Polar Lipids	850155 P
DGS-NTA(Ni)	Avanti® Polar Lipids	790404 C
Trichloroacetic Acid	Sigma	T6399
**Software**		
Fiji-ImageJ2	NIH	RRID:SCR_003070
FlowJo version 10	FlowJo LLC	Version 10
GraphPad Prism	GraphPad	Version 6
R	R Foundation	Version 4.4.1
Proteome Discoverer software	Thermo Fisher Scientific	version 1.4
Mascot	Matrix Science	
**Other**		
PepMute^™^ siRNA transfection reagent	SignalGen^®^	SL100566
FuGENE^®^ 6 Transfection Reagent	Promega	E2691
Frozen-EZ yeast transformation II kit	Zymo Research	T2001
Reverse transcription kit	PROtech	PT-RT-KIT
Fast SYBR™ Green Master Mix	Thermo	4385612
Falcon^®^ Round Bottom Polystyrene Tubes	Corning	Cat. No. 352052
Orbitrap Elite linear ion trap mass spectrometer	Thermo Fisher Scientific	Orbitrap Elite
CFX Connect Real-Time PCR system	Bio-Rad	CFX Connect
Eclipse Ti2 TIRF microscope	Nikon	Ti2

### Plasmids

Wild-type PGK1, an inactive PGK1 mutant (T378P), and DKF290-292AAA (290-292 A) were cloned and inserted into the BamHI and XhoI sites of pcDNA3.1-myc-His(-) (Invitrogen) and pEGFP-C3 (Clontech). HA-tagged PGK1 was also cloned and inserted into the BamHI and XhoI sites of pcDNA3.1-myc-His(-) with a stop codon after the HA tag, and mCherry was cloned and inserted into the XbaI site of pcDNA3.1-myc-His(-), after which PGK1 was cloned and inserted into the EcoRI and BamHI sites with a stop codon. For mRFP-PGK1, PGK1 was cloned and inserted into the HindIII sites of pCMV6-AN-mRFP (OriGene) with a stop codon. PGK1 wild-type and 290-292A was cloned and inserted into the BamHI and XhoI sites of the wild-type pGEX4T-3 (Amersham) vector; PGK1 wild-type were cloned and inserted into the BamHI and XhoI sites of pJG4-5. AP2A was cloned and inserted into the XhoI and HindIII sites of pcDNA3.1-myc-His(-) and pIEx-6 (Novagen); AP2A was cloned and inserted into the EcoRI and XhoI sites of pEG202 (NovoPro). For α-mCherry, mCherry was cloned and inserted into the HindIII site of pcDNA3.1-myc-His(-), after which AP2A was cloned and inserted into the EcoRI and BamHI sites. AP2Α dH was cloned and inserted into the BamHI and XhoI sites for AP2A 1-624 and the XhoI and HindIII sites for AP2A 702-941 of pcDNA3.1-myc-His(-). AP2M was cloned and inserted into the XhoI and EcoRV sites of pET32a (Invitrogen). AP2S was cloned and inserted into the XhoI and BamHI sites of pET32a (Invitrogen). For truncated α: 1-624, 625-941, and 702-941 were cloned and inserted into the XhoI and BamHI sites of pET32a and pET30a (Invitrogen). Cargoes peptides used in GST-pulldown assay: For the TfR cytosolic tail, TfR1-61 (TfR cyto) wild-type and Y20A mutant strains were cloned and inserted into pGEX4T-3. For the EGFR cytoplasmic tail containing tyrosine motif, EGFR 980-1017 wild-type and Y998A mutant strains were cloned and inserted into the BamHI sites of pGEX4T-3. For the EGFR cytoplasmic tail containing di-leucin motif, EGFR 1013-1054 wild-type and L1034AL1035A mutant strains were cloned and inserted into the BamHI sites of pGEX4T-3.

Cargoes peptides used in Yeast-two hybrid: For the TfR cytosolic tail, TfR1-61 (TfR cyto) wild-type and Y20A mutant strains were cloned and inserted into the EcoRI and XhoI sites of pEG202. For the EGFR cytoplasmic tail containing tyrosine motif, EGFR 992-1005 wild-type, and Y998A mutant strains were cloned and inserted into the BamHI sites of pEG202. For the EGFR cytosolic tail containing di-leucin motif, EGFR 1018-1045 wild-type, and L1034AL1035A mutant strains were cloned and inserted into the BamHI sites of pEG202.

### Antibodies

The following antibodies were used: anti-EEA1 (Cell Signalling 2411, immunofluorescence, 1:100/western blotting, 1:1000), anti-Myc (Cell Signaling 2276, immunofluorescence, 1:100/western blotting, 1:1000), anti-6×His (Santa Cruz sc-803, western blotting, 1:500), anti-HA (Santa Cruz sc-7392, western blotting, 1:1000), anti-PGK1 (Santa Cruz sc-130335, immunofluorescence, 1:100/western blotting, 1:2000) (GeneTex GTX107614, immunofluorescence, 1:100), anti-α (BD Transduction Laboratories 610502, immunofluorescence, 1:100/western blotting, 1:1000), anti-μ2 (BD Transduction Laboratories 611351, western blot, 1:500), anti-β2 (Santa Cruz sc-74423, western blotting, 1:1000), anti-σ2 (ORIGENE TA373563, western blotting, 1:500), anti-clathrin heavy chain (Cell Signaling 2410, western blotting, 1:1000), anti-α-tubulin (Cell Signaling 3873, western blotting, 1:5000), anti-EGFR (Cell Signaling 4267, immunofluorescence, 1:100/western blotting, 1:1000), anti-TfR (Santa Cruz sc-65882, western blotting, 1:500).

### Cell culture

All mammalian cells were cultured at 37 °C with 5% CO_2_. HeLa and 293T cells were cultured in Dulbecco’s modified Eagle’s medium (DMEM) with high glucose supplemented with 10% fetal bovine serum (FBS) and 1% penicillin‒streptomycin solution (PS). Unless otherwise indicated, all experiments were performed using HeLa cells. For siRNA transfection, PepMute^™^ siRNA transfection reagent was used to silence target genes. For plasmid transfection, FuGENE^®^ 6 Transfection Reagent was used for transient expression.

Insect ExpiSf9™ cells were cultured at 25 °C in ExpiSf™ CD medium. For plasmid transfection, PEI 25 K™ was used for transient expression.

The yeast YEM1α (*MATα*, *trp1*, *his3*, *leu2*, *6ops*-*LEU2*, *2ops*-*LacZ*) strain was used to conduct a yeast two-hybrid assay and was maintained in yeast extract peptone dextrose (YPD) medium containing 1% yeast extract, 2% peptone, 2% glucose or synthetic dextrose (SD) medium containing 0.67% yeast nitrogen base (YNB), 2% glucose, and NaOH (pH = 8) with an appropriate amino acid mixture. For plasmid transformation, a Frozen-EZ yeast transformation II kit was used. To express the desired proteins, 2% galactose was used to replace glucose and induce protein expression under the gal promoter.

### Endocytosis assay

To examine cargo transport from the plasma membrane to the early endosome, the cells were cultured in serum-free medium for 1 h and then allowed to bind to the cargo (5 μg/mL for Tf, 0.1 μg/mL for EGF, or 5 μg/mL for CTxB) for 1 h at 4 °C. The cells were then incubated at 37 °C and examined at the indicated time points. For those needed to measure the internalized Tf or EGF intensity, cells were first washed by the acid buffer, 0.2 M acetic acid and 0.5 M NaCl, and then fixed. Fixed cells were stained for EEA1 and observed via confocal microscopy to assess the colocalization of the cargoes and EEA1. Images were analysed with Fiji-ImageJ2.

### Internalization index measured by flow cytometry

To examine Tf internalization, serum-starved cells were incubated with Alexa Fluor 488-conjugated Tf at 4 °C for 1 h. To measure Tf levels, the cells were fixed and collected in polystyrene tubes. To measure internalized Tf levels, cells were then incubated at 37 °C.

To quantify Tf internalization at constant 37 °C, serum-starved cells were incubated with Alexa Fluor 488-conjugated Tf and maintained at 37 °C for 0, 2, and 5 min, following by acidic buffer quenching. As a negative control, cells were pre-treated with 80 µM dynasore for 10 min to inhibit endocytosis (Gulbranson et al, 2019).

For trypan blue quenching, fixed cells were treated with 2% trypan blue in phosphate-buffered saline (PBS) to quench surface Tf (Patiño et al, 2015). For acidic buffer quenching, cells were washed with acidic buffer (0.2 M acetic acid and 0.5 M NaCl) before fixation. The fluorescence intensity of approximately 10,000 cells at 495 nm was analyzed using a BD FACSCalibur flow cytometer. Data were analysed with FlowJo version 10.

For trypan blue-treated groups, the internalization index was calculated as: *Internalization Index* = *(I*_*TB*_*(t)- I*_*TB*_*(0))/I(0)*. where *I* represents relative fluorescence intensity at 495 nm, *TB* indicates trypan blue treatment, and *t* represents incubation time (minutes).

For acidic wash groups, the internalization index was calculated as: *Internalization Index* = *(I*_*A*_*(t)- I(0))/I(t)*, where A indicates cells washed with acidic buffer.

### Recombinant protein purification

*Escherichia coli* (*E. coli*) strain BL21 was transformed with plasmids, pGEX-4T-3 for the GST-tag, and pET30a or pET32a for the His-tag. The transformed E. *coli* were harvested at the exponential growth phase and induced to express recombinant proteins with 500 μM isopropyl-β-thiogalactoside (IPTG) at 37 °C for 3 h. For the GST tag, GST-tagged PGK1 or GST-tagged cargoes, the lysates were purified in PBS with glutathione-Sepharose 4B, and the coexpression of GST-PGK1 and truncated α also followed the protocol of GST protein purification. For His-tagged truncated α, lysates were lysed and pulled down with nickel affinity resin in 50 mM NaH_2_PO_4_-NaOH (pH = 8.0), 300 mM NaCl, and 10 mM imidazole, followed by elution with 250 mM imidazole. For protein storage, the buffer was exchanged for PBS by dialysis (12–14 kDa). For His-tagged μ2 and σ2, lysates were lysed and pulled down with nickel affinity resin in 20 mM Tris-HCl (pH = 8.0), 500 mM NaCl, 5 mM imidazole, and 6 M urea, after which the proteins were eluted with 250 mM imidazole without urea.

For His-tagged full-length α, pIEx-6 was transiently expressed in ExpiSf9™ cells. Lysates were lysed and pulled down with nickel affinity resin in 50 mM NaH_2_PO_4_-NaOH (pH=8.0) with 300 mM NaCl and 10 mM imidazole, followed by elution with 250 mM imidazole. For protein storage, the buffer was exchanged with PBS by dialysis. Recombinant proteins μ2 subunit (#EP001872HUa0) were purchased from Cusabio. Recombinant proteins σ2 subunit (#MBS954417) were purchased from MyBioSource. Recombinant proteins β2 subunit (#TP301129) were purchased from OriGene Technologies.

### Pull-down assay

To assess the binding of PGK1 to the AP2 components, GST-PGK1 with glutathione beads was incubated with the AP2 components in 50 mM KCl, 25 mM HEPES-KOH (pH = 7.2) with 2.5 mM Mg(OAc)_2,_ and a protease inhibitor for 1 h at 4 °C. The beads were washed following analysis via gel electrophoresis, followed by either western blotting with an anti-His antibody or Coomassie blue staining.

To assess the binding of PGK1 to truncated α, GST-PGK1 with glutathione beads was incubated with truncated α in 50 mM HEPES-KOH (pH = 7.2) with 300 mM NaCl, 1 mM EDTA, 0.5% NP40 and protease inhibitor for 1 h at 4 °C. The beads were washed following analysis via gel electrophoresis, followed by either western blotting with an anti-His antibody or Coomassie blue staining.

### Immunoprecipitation

Mammalian cells were lysed in 50 mM Tris-HCl (pH = 7.4), 150 mM NaCl, 0.5% Triton X-100, 20 mM NaF, protease inhibitors, and phosphatase inhibitors. Following a 10-min incubation on ice, the lysates were clarified by centrifugation at 15,000×*g* for 10 min at 4 °C. The resulting supernatants were incubated with anti-c-Myc agarose conjugate or anti-HA agarose conjugate at 4 °C for 2 h. The beads were washed three times and subjected to western blot analysis or mass spectrometry.

### Mass spectrometry analysis

Proteins isolated from the immunoprecipitation assay were separated by 12.5% SDS-PAGE and visualized using Coomassie Brilliant Blue G-250 staining. The gel bands were excised and in-gel tryptic digestion was performed according to previously established protocols (Liu et al, 2015). The resulting peptide mixtures were extracted with acetonitrile to achieve a final concentration of 50% and subsequently dried using a SpeedVac. For the liquid chromatography tandem-mass spectrometry analysis, each peptide mixture was reconstituted in HPLC buffer A (0.1% formic acid, Sigma) and introduced into a trap column (Zorbax 300 SB-C18, 0.3 × 5 mm), where they were washed with buffer A. The desalted peptides were then separated on a 10 cm analytical C18 column with an inner diameter of 75 μm. The liquid chromatography system was linked to a linear ion trap mass spectrometer (Orbitrap Elite, Thermo Fisher Scientific). Full-scan mass spectrometry was conducted with the Orbitrap over a mass range of 400–2000 Da, achieving the detection of intact peptides at a resolution of 30,000. To identify the proteins, the raw data from the mass spectrometry were processed using Proteome Discoverer software (version 1.4, Thermo Fisher Scientific). The obtained mass spectra were searched against the SwissProt database (Taxonomy: Homo sapiens) through the Mascot search engine (Matrix Science). The search parameters included trypsin as the digestion enzyme, allowing up to two missed cleavages. Carbamidomethylation (C) was set as a static modification, while dynamic modifications included oxidation (M), N-acetylation (protein), and Gln to pyro-Glu (N-term Q). The tolerances were set at 10 ppm for MS and 0.7 Da for MS/MS.

### Immunofluorescence staining and PLA

Cells seeded on coverslips were fixed with 2% paraformaldehyde in PBS for 10 min and permeabilized in PBS with 3% BSA and 0.1% saponin for 20 min at room temperature. Primary and secondary antibodies were diluted in PBS with 10% FBS, 0.02% NaN_3_, and 0.2% saponin and incubated with coverslips for 1 h at room temperature. After immunostaining, the coverslips were mounted on slides with Fluoromount-G™ Mounting Medium with DAPI. To pre-lyse the cell membrane, release cytosolic PGK1, and increase the endosomal PGK1 signal, coverslips were pre-treated with 0.0625% saponin in PBS for 2 min at room temperature before fixation (Hinckelmann et al, 2016).

For the PLA, following the protocol of the immunostaining and PLA kit (Duolink® In Situ), cells seeded on coverslips were fixed with 2% paraformaldehyde in PBS for 10 min and permeabilized in PBS with 3% BSA and 0.1% saponin for 20 min at room temperature. Primary antibodies were diluted in PBS with 10% FBS, 0.02% NaN_3_, and 0.2% saponin for 1 h at room temperature and washed twice with wash buffer A. The cells were incubated with a mixture of PLA® probe anti-rabbit PLUS and PLA® probe anti-mouse MINUS for 1 h at 37 °C and washed twice with wash buffer A. To ligate the two probes, the cells were incubated with ligation buffer and ligase for 30 min at 37 °C and washed twice with wash buffer A. To amplify the PLA signal, the cells were incubated with amplification buffer and polymerase for 100 min at 37 °C and washed twice with wash buffer B. The cells were then mounted on slides with Fluoromount-G™ Mounting Medium with DAPI.

### Yeast two-hybrid assay

Yeast cells transformed with pJG4-5 and pEG202 were plated on a synthetic galactose (SG) plate (2% galactose) with His, Trp dropout (-His-Trp), and His, Trp, and Leu dropout (-His-Trp-Leu) plates were incubated at 30 °C for the appropriate time. Protein‒protein interactions enabled the growth of yeast cells on His, Trp, and Leu dropout plates.

### Cycloheximide chase assay

The cells were treated with 10 μg/mL CHX for the indicated time and then were lysed in 50 mM Tris-HCl (pH = 7.4), 150 mM NaCl, 5 mM EDTA, 1% Triton X-100, protease inhibitors, and phosphatase inhibitors, followed by western blot analysis. To determine whether degradation occurs through the proteasome, the cells were co-incubated with 30 mM MG132 and 10 μg/mL CHX for 18 h, lysed, and then subjected to analysis via western blotting.

### Quantitative PCR (qPCR)

Total RNA was isolated and transformed into cDNA with a reverse transcription kit and Oligo (dT)_18_ primers. Gene expression was quantified via Bio-Rad CFX Connect qPCR using Fast SYBR™ Green Master Mix with the indicated gene primers. The gene expression of ACTIN was used as an internal control.

### Streptavidin pull-down assay

Serum-starved cells were incubated with 5 μg/mL biotin-labelled transferrin and 100 ng/mL biotin-labelled EGF at 4 °C for 1 h and then washed with unbound. The cells were lysed in 50 mM Tris-HCl (pH = 7.4), 150 mM NaCl, 0.5% Triton X-100, 20 mM NaF, protease inhibitors, and phosphatase inhibitors. The supernatants were incubated with streptavidin-agarose at 4 °C for 1 h. The beads were washed three times and subjected to Western blot analysis.

### Liposome recruitment assay

The liposomes contained 50% phosphatidylcholine (DOPC), 40% phosphoethanolamine (DOPE), and 10% 18:1 phosphatidylinositol-4,5-bisphosphate (PI(4,5)P_2_). To capture the peptides, the liposomes contained 40% DOPC, 40% DOPE, 10% PI(4,5)P_2_, and 10% 1,2-dioleoyl-sn-glycero-3-[(N-(5-amino-1-carboxypentyl) iminodiacetic acid) succinyl] (nickel salt) (DGS-NTA(Ni)) (Kelly et al, 2014). Liposomes were rehydrated in 25 mM HEPES-KOH (pH 7.2), 50 mM KCl, 2.5 mM Mg(OAc)_2_, and 200 mM sucrose overnight and passed through a 400 nm filter with a miniextruder. His-diLeu peptides (HHHHHHVEPLTPSGEAPNQALL) and His-Tyr peptides (HHHHHHSAFSNLFGGEPLSYTRFSL) were synthesized by our synthesis core facility (Synthesis Core Facility, Institute of Biological Chemistry, Academia Sinica), and 20 μg liposomes were preincubated with 500 nM cargo peptides at 37 °C for 1 h if needed. After preincubation, 20 μg of liposomes were incubated with 300 nM GST-PGK1, 300 nM His-α, 300 nM μ2, and 300 nM σ2, as indicated, in 25 mM HEPES-KOH (pH 7.2), 50 mM KCl, and 2.5 mM Mg(OAc)_2_ at 37 °C for 1 h. After centrifugation, the liposomes were pelleted, and the supernatants were collected and precipitated with 10% trichloroacetic acid. Both the supernatants and pellets were subjected to western blot analysis.

### TIRF microscopy and image analysis

All the confocal images were obtained with a Nikon ECLIPSE Ti2 TIRF microscope with a Plan Apochromat TIRF ×100/1.49 NA objective. To examine the Tf level on the plasma membrane, serum-starved cells were incubated with Alexa Fluor 555-conjugated Tf at 4 °C for 1 h. To measure the initial surface Tf level, cells were fixed at 4 °C. To measure the remaining level of surface Tf after internalization, the cells were shifted to 37 °C and then fixed.

Cells were observed in PBS via TIRF microscopy. For colocalization quantification, images were analysed via *Fiji-ImageJ2*. *Apply_DOG_Filtering.py* was used to subtract the background fluorescence, followed by threshold adjustment using *threshold_adjuster*. Colocalization for thresholded Manders’ split coefficient and object-based analysis were performed using *JACoP v2.0* (Bolte and Cordelières, 2006).

### Confocal microscopy and image analysis

All the confocal images were obtained with a ZEISS LSM 900 confocal microscope with a Plan Apochromat ×63/1.4 NA objective, and super-resolution confocal images were obtained with a ZEISS LSM 900 with Airyscan 2. Images were analysed via Fiji-ImageJ2. Colocalization was measured via Coloc2 (imagej.net/plugins/coloc-2), expressed as the thresholded Manders’ split coefficient (tM1 or tM2). These coefficients quantify the fraction of fluorescence intensity in one channel that is present in pixels where the second channel also contains signal above the applied threshold. Automatic thresholding parameters were applied identically across experimental groups to minimize potential bias in colocalization measurements. Pearson’s correlation coefficient (PCC; Pearson’s *R* value without thresholding) was additionally calculated as an independent metric to assess the linear correlation between fluorescence intensities of the two channels. PCC analysis yielded results consistent with those obtained using the thresholded Manders’ split coefficients.

Intensity profiles were generated using the RGB Profiler plugin (imagej.net/plugins/rgb-profiler) in Fiji-ImageJ2.

### Statistical analysis

The quantitative data are shown as the means +/− SDs. Statistical significance was determined using Excel, Prism software, and R-4.4.1. The experiments were not randomized, and the investigators were not blinded to allocation during experiments and data assessment.

## Supplementary information

Appendix

Peer Review File

Dataset EV1

Source data Fig. 1

Source data Fig. 2

Source data Fig. 3

Source data Fig. 4

Source data Fig. 5

Source data Fig. 6

Source data Fig. 7

Source data Fig. 8

Appendix Figure S1-S4 Source Data

Appendix S5-S8 Figure Source Data

## Data Availability

This study includes no data deposited in external repositories. The source data of this paper are collected in the following database record: biostudies:S-SCDT-10_1038-S44319-026-00948-5.
