# Supplementary material for: Phosphoglycerate kinase 1 cooperates with the AP2 complex to promote endocytosis
Source: EMBO Rep. 2026 Oct 1;27(21):6447–63. doi: 10.1038/s44319-026-00948-5 (PMC13743783; doi:10.1038/s44319-026-00948-5)
Supplement: Supplementary file 1 [file 44319_2026_948_MOESM1_ESM.pdf]

Appendix

Phosphoglycerate kinase 1 cooperates with the AP2 complex to promote endocytosis

Table of Contents

Appendix Figure S1 .... **2-3**

Appendix Figure S2 .... **4**

Appendix Figure S3 .... **5**

Appendix Figure S4 .... **6-7**

Appendix Figure S5 .... **8-9**

Appendix Figure S6 .... **10-11**

Appendix Figure S7 .... **12-13**

Appendix Figure S8 .... **14**

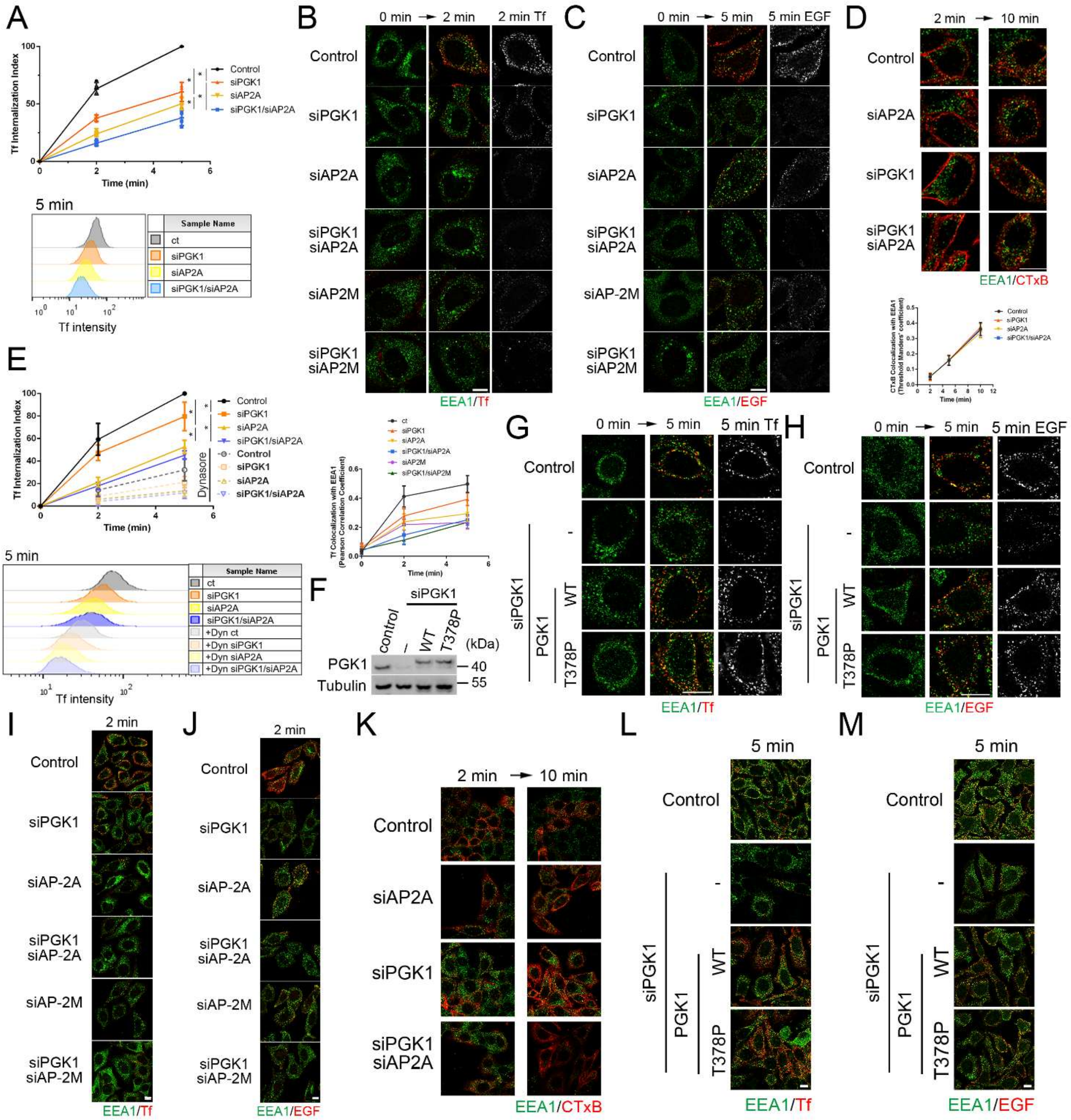

Appendix Figure S1. PGK1 regulates AP2-mediated endocytosis. Quantitative analyses of cells treated with the indicated siRNAs are shown as the means ± SDs. *P<0.05

(A) Internalization of Tf quantified by flow cytometry at 0, 2, and 5 minutes (n > 10,000 cells, four independent biological replicates). Representative flow cytometry histograms showing internalized Tf fluorescence intensity at the 5-min time point. One-way ANOVA with Tukey's test at the 5-minute time point: control vs. siPGK1

(P=8.7x10^{-7}); control vs. siAP2A (P=7.0x10^{-9}); siAP2A vs. siPGK1/ siAP2A (P=0.025); siPGK1 vs. siPGK1/ siAP2A (P=4.8x10^{-6}).

(B) Image (upper; scale bar, 10 μm) and quantification (lower) showing internalized Tf (red) colocalized with EEA1 (green), expressed as Pearson correlation coefficient (n=20 cells of one representative experiment from two independent biological replicates).

(C) Representative image of internalized EGF (red) colocalized with EEA1 (green); scale bar, 10 μm.

(D) Image (upper; scale bar, 10 μm) and quantification (lower) showing CTxB (red) colocalized with EEA1 (green), expressed as thresholded Manders' coefficient (n=15 cells of one representative experiment from two independent biological replicates).

(E) Tf Internalization at constant 37˚C quantified by flow cytometry (n>10,000 cells, six independent biological replicates); dynasore treatment as negative control. Representative flow cytometry histograms showing internalized Tf fluorescence intensity at the 5-min time point. One-way ANOVA with Tukey's test at the 5-minute time point: control vs. siPGK1 (P=0.022); control vs. siAP2A (P=1.7x10^{-9}); siAP2A vs. siPGK1/ siAP2A (P=0.0043); siPGK1 vs. siPGK1/ siAP2A (P=0.0106).

(F) Immunoblot analysis of HEK293T cell lysates to examine the rescue effects of PGK1 and PGK1^{T378P} in siPGK1 cells (n=3 independent biological replicates).

(G) Representative image of internalized Tf (red) colocalized with EEA1 (green); scale bar, 10 μm.

(H) Representative image of internalized EGF (red) colocalized with EEA1 (green); scale bar, 10 μm.

(I) Representative image with a larger field of view corresponding to Appendix Fig. S1B.

(J) Representative image with a larger field of view corresponding to Appendix Fig. S1C.

(K) Representative image with a larger field of view corresponding to Appendix Fig. S1D.

(L) Representative image with a larger field of view corresponding to Appendix Fig. S1G.

(M) Representative image with a larger field of view corresponding to Appendix Fig. S1H.

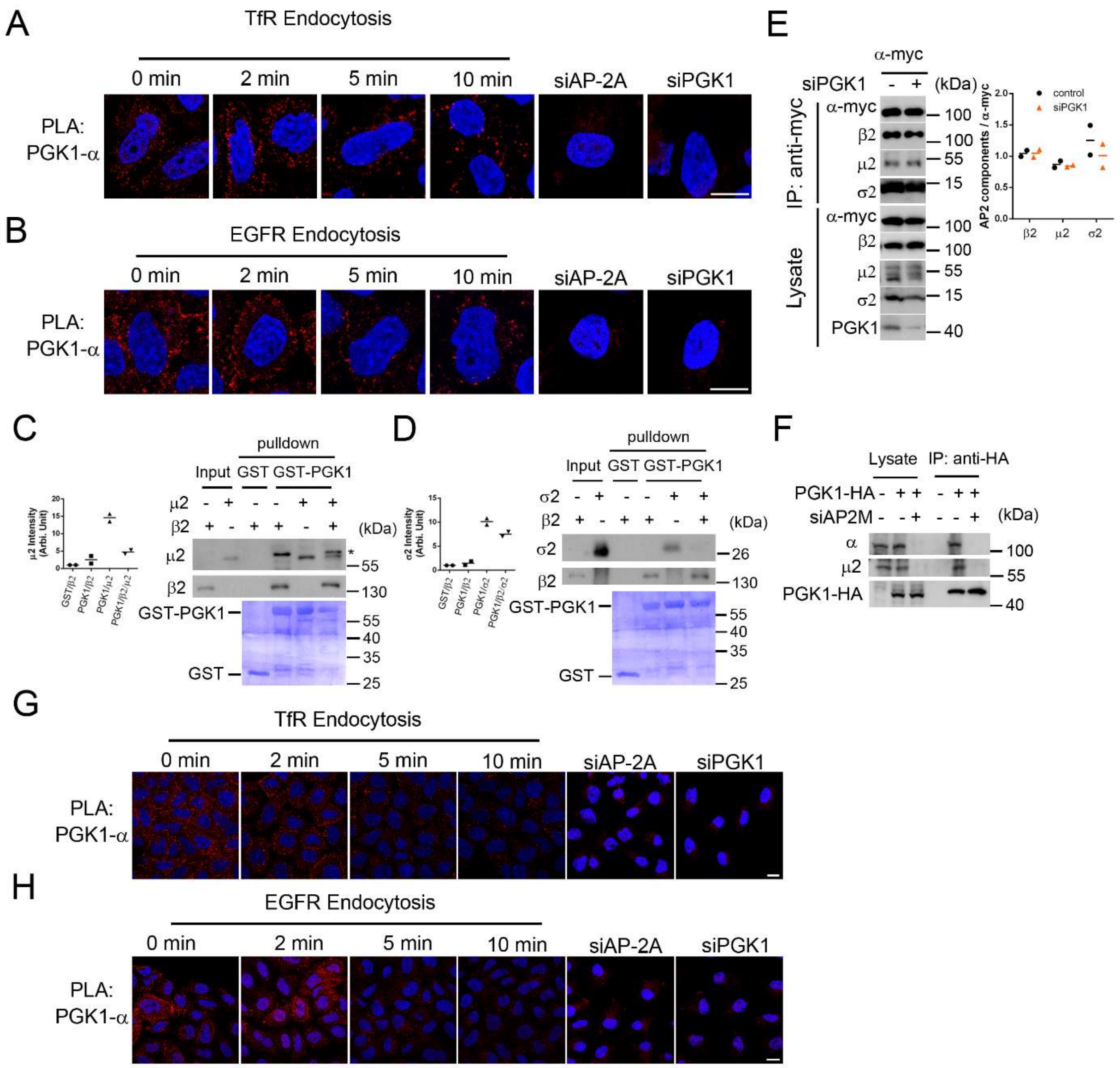

Appendix Figure S2. PGK1 interacts with α subunit

(A) PLA analysis showing the interaction between PGK1 and α during TfR endocytosis. Red indicates PLA puncta and the blue indicates nuclear staining; scale bar, 10 μm.

(B) PLA analysis showing the interaction between PGK1 and α during EGFR endocytosis. Red indicates PLA puncta and the blue indicates nuclear staining; scale bar, 10 μm.

(C) Pull-down assay examining the interaction of GST-PGK1 with purified β2 and μ2. The quantification shows relative μ2 intensity (two independent biological replicates).

(D) Pull-down assay examining the interaction of GST-PGK1 with purified β2 and σ2. The quantification shows relative σ2 intensity (two independent biological replicates).

(E) Co-immunoprecipitation analysis examining the effect of PGK1 siRNA on the association of myc-tagged α with endogenous β2, μ2, and σ2 in HEK293T cells (two independent biological replicates).

(F) Co-immunoprecipitation analysis examining the effect of siAP2M on the association of HA-tagged PGK1 with α and μ2 (two independent biological replicates).

(G) Representative image with a larger field of view corresponding to Appendix Fig. S2A.

(H) Representative image with a larger field of view corresponding to Appendix Fig. S2B.

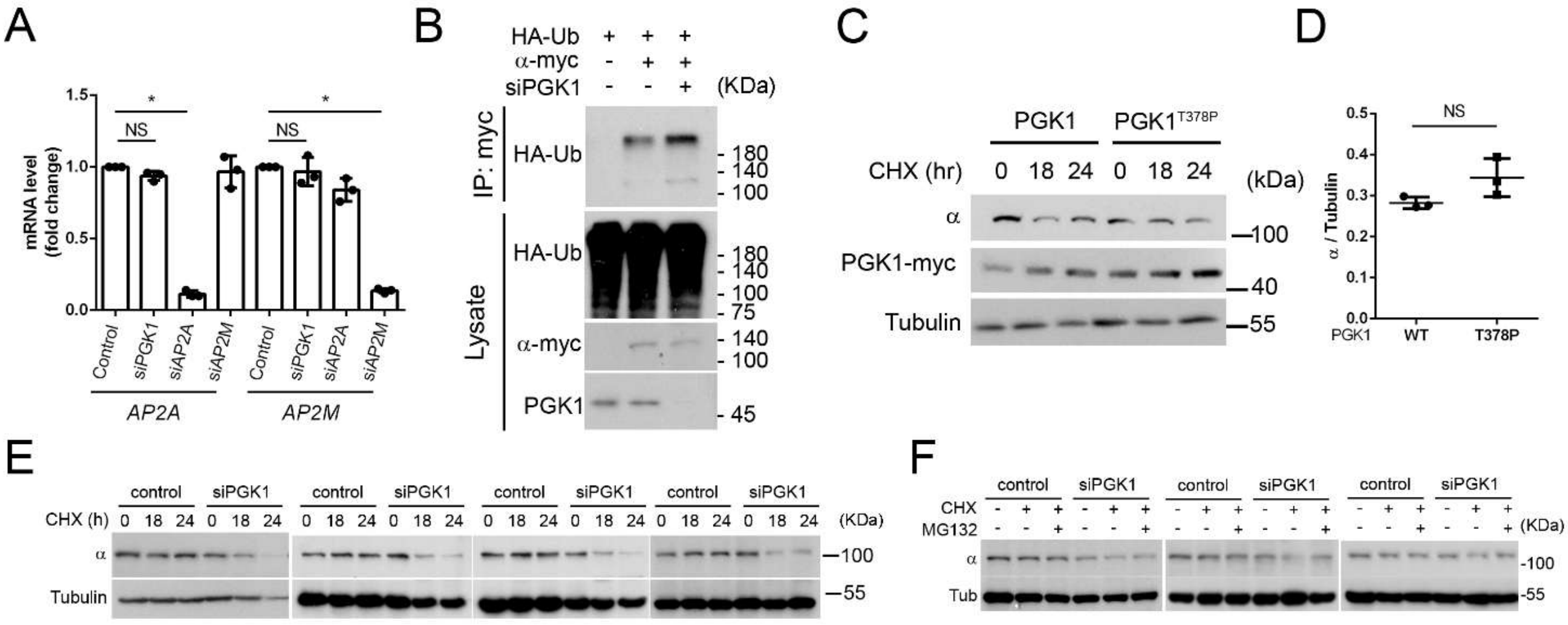

Appendix Figure S3. PGK1 affects the stability of AP2 components. Data are shown as the means ± SDs. *P<0.05 NS P>0.05

(A) Total mRNA levels of *AP2A* and *AP2M* (n=3 independent biological replicates). One-way ANOVA with Tukey's test: *AP2A* –control vs. siPGK1 (P=0.4535), control vs. siAP2A (P=2.1x10^{-13}); *AP2M* – control vs. siPGK1 (P=0.8624), control vs. siAP2M (P=9.4x10^{-11}).

(B) Immunoprecipitation of myc-tagged α subunit from cells expressing HA-Ub, with or without PGK1 knockdown (siPGK1), examining the increase of ubiquitinated α in siPGK1 cells (two independent biological replicates).

(C) Time course analysis of α degradation upon CHX treatment to examine the effects of PGK1 and PGK1^{T378P}.

(D) Quantification of the α protein level normalized to the Tubulin level at 24 hours (three independent biological replicates). Paired Student's t test, P=0.182.

(E) Additional representative immunoblots corresponding to Fig. 3C.

(F) Additional representative immunoblots corresponding to Fig. 3E.

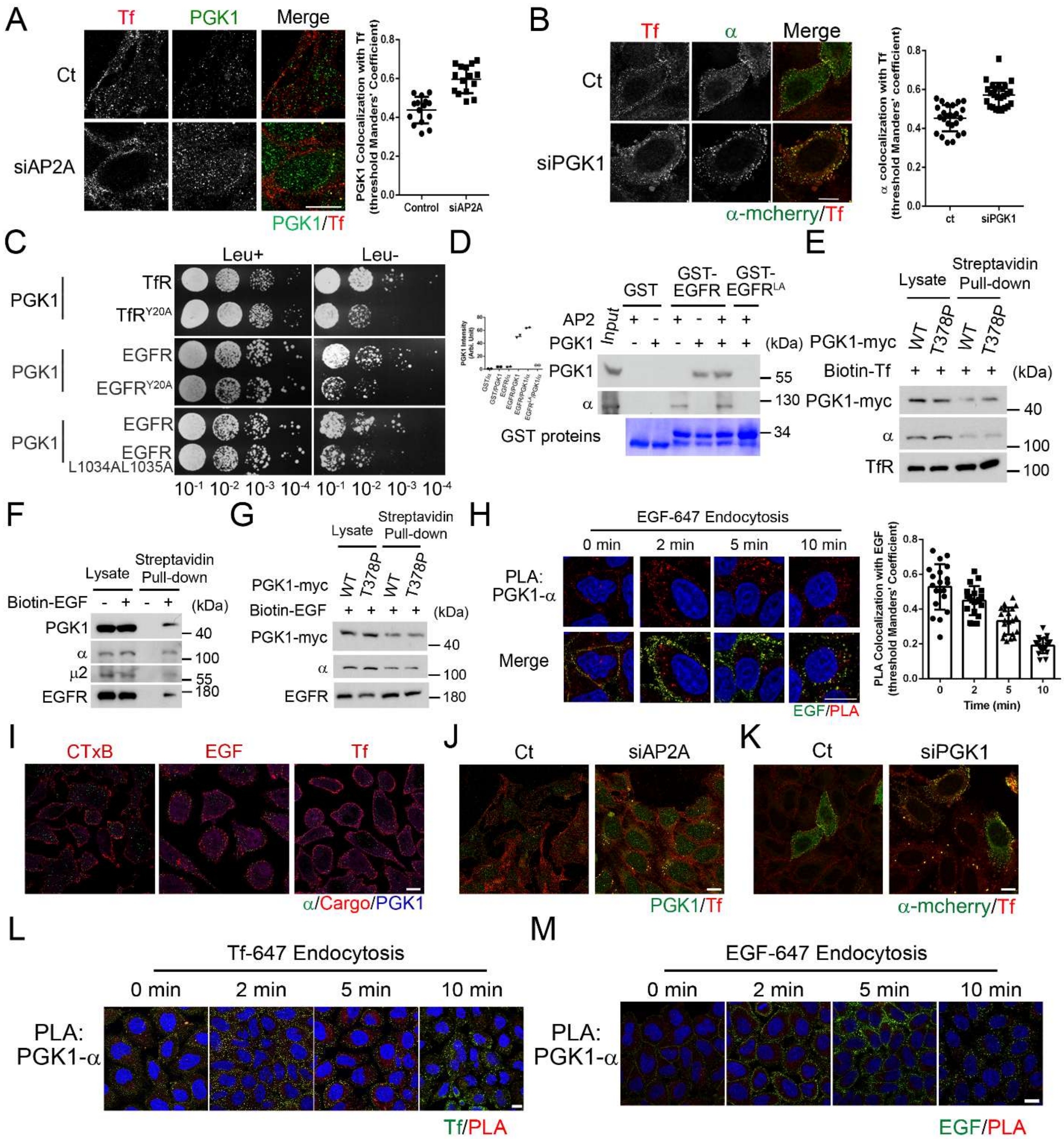

Appendix Figure S4. PGK1 recognizes cargo proteins to regulate endocytosis. Data are shown as the means ± SDs.

(A) Immunostaining after pre-lysis extraction (left; scale bar, 10 μm) and quantification (right) showing PGK1 (green) colocalized with Tf (red) in siAP2A cells after 2 minutes of internalization, expressed as the thresholded Manders' coefficient (n=15 cells of one representative experiment from two independent biological replicates).

(B) Image (left; scale bar, 10 μm) and quantification (right) showing AP-2α-mCherry (green) colocalized with Tf (red) in siPGK1 cells after 1 minute of internalization, expressed as the thresholded Manders' coefficient (n=25 cells of one representative experiment from two independent biological replicates).

(C) Yeast two-hybrid assay examining the interaction of PGK1 with sorting motifs on the cytoplasmic tail of TfR and EGFR and their mutations (three independent biological replicates).

(D) *In vitro* binding analysis of His-PGK1 and GST-EGFR fragments via GST pull-down to analyse the binding effect in the presence of α. The quantification shows relative PGK1 intensity (two independent biological replicates).

(E) Myc-tagged PGK1 and PGK1^{T378P} associated with surface TfR and α (three independent biological replicates).

(F) Surface EGFR associated with endogenous PGK1 and AP2 complexes (three independent biological replicates).

(G) Myc-tagged PGK1 and PGK1^{T378P} associated with surface EGFR and α (three independent biological replicates).

(H) PLA analysis showing colocalization of PGK1—α PLA puncta with EGF during EGFR endocytosis (n=20 cells of one representative experiment from independent two biological replicates); scale bar, 10 μm.

(I) Representative image with a larger field of view corresponding to Fig. 4A.

(J) Representative image with a larger field of view corresponding to Appendix Fig. S4A.

(K) Representative image with a larger field of view corresponding to Appendix Fig. S4B.

(L) Representative image with a larger field of view corresponding to Fig. 4E.

(M) Representative image with a larger field of view corresponding to Appendix Fig. S4H.

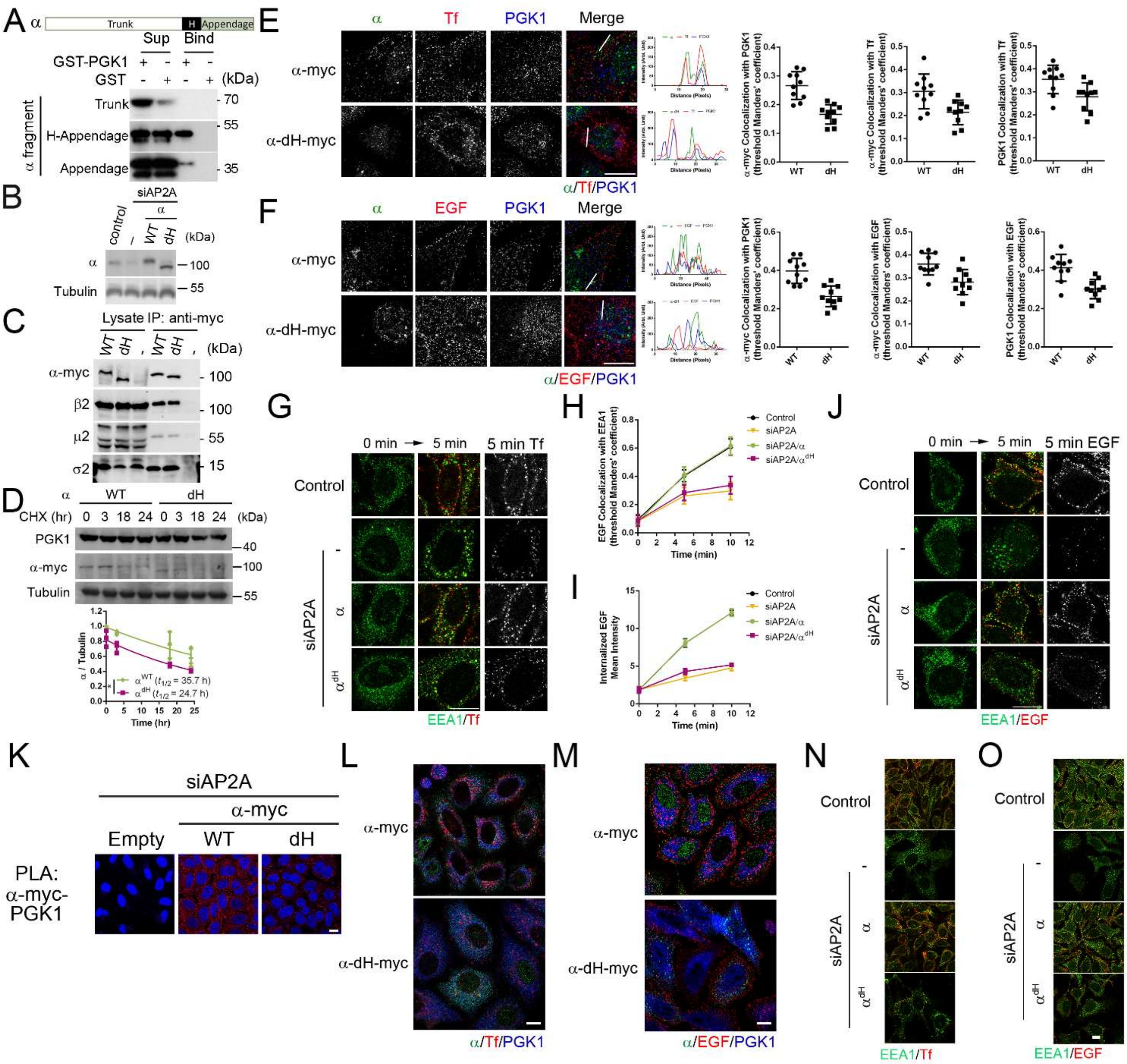

Appendix Figure S5. The hinge region of α subunit is required for its interaction with PGK1. Data are shown as the means ± SDs. *P<0.05

(A) GST-PGK1 pull-down showing association with different His-tagged α fragments (three independent biological replicates). Top, schematic representation of α trunk, hinge (H), and appendage domains.

(B) Immunoblot analysis of HEK293T cell lysates to examine the rescue effects of α and α^{dH} in siAP2A cells, (n=3 independent biological replicates).

(C) Immunoprecipitation of myc-tagged α and α^{dH} with AP-2 components in HEK293T cells (three independent biological replicates).

(D) Time course analysis of α degradation upon CHX treatment, examining α and α^{dH}. Protein levels of α were normalized to Tubulin and plotted against time (three independent biological replicates). Curves were fitted using a one-phase decay non-linear regression model. The calculated half-life ($t_{1/2}$) is 35.7 h for the α and 24.7 h for α^{dH}. Curves were compared using an extra sum-of-squares F-test (P=0.0075).

(E) Confocal images after pre-lysis extraction (left; scale bar, 10 μm), intensity profiles (middle) show the relative fluorescence intensities along the white lines in the merge panels, and quantification (right), expressed as thresholded Manders' coefficient, showing the effects of myc-tagged α and α^{dH} on colocalization of α-myc, Tf, and PGK1 during Tf internalization at 2 minutes (n=10 cells of one representative experiment from two independent biological replicates).

(F) Confocal images after pre-lysis extraction (left; scale bar, 10 μm), intensity profiles (middle) show the relative fluorescence intensities along the white lines in the merge panels, and quantification (right), expressed as thresholded Manders' coefficient, showing the effects of myc-tagged α and α^{dH} on colocalization of α-myc, EGF, and PGK1 during EGF internalization at 2 minutes (n=10 cells of one representative experiment from two independent biological replicates).

(G) Representative image of Tf (red) colocalized with EEA1 (green); scale bar, 10 μm.

(H) Colocalization of EGF with EEA1, expressed as thresholded Manders' coefficient, comparing the effects of α with α^{dH} in siAP2A cells at 0, 5, and 10 minutes (n=15 cells of one representative experiment from independent two biological replicates).

(I) Mean intensity of internalized Tf comparing the effects of α with α^{dH} in siAP2A cells at 0, 5, and 10 minutes (n=15 cells of one representative experiment from two independent biological replicates).

(J) Representative image of EGF (red) colocalized with EEA1 (green); scale bar, 10 μm.

(K) Representative image with a larger field of view corresponding to Fig. 5D.

(L) Representative image with a larger field of view corresponding to Appendix Fig. S5E.

(M) Representative image with a larger field of view corresponding to Appendix Fig. S5F.

(N) Representative image with a larger field of view corresponding to Appendix Fig. S5G.

(O) Representative image with a larger field of view corresponding to Appendix Fig. S5J.

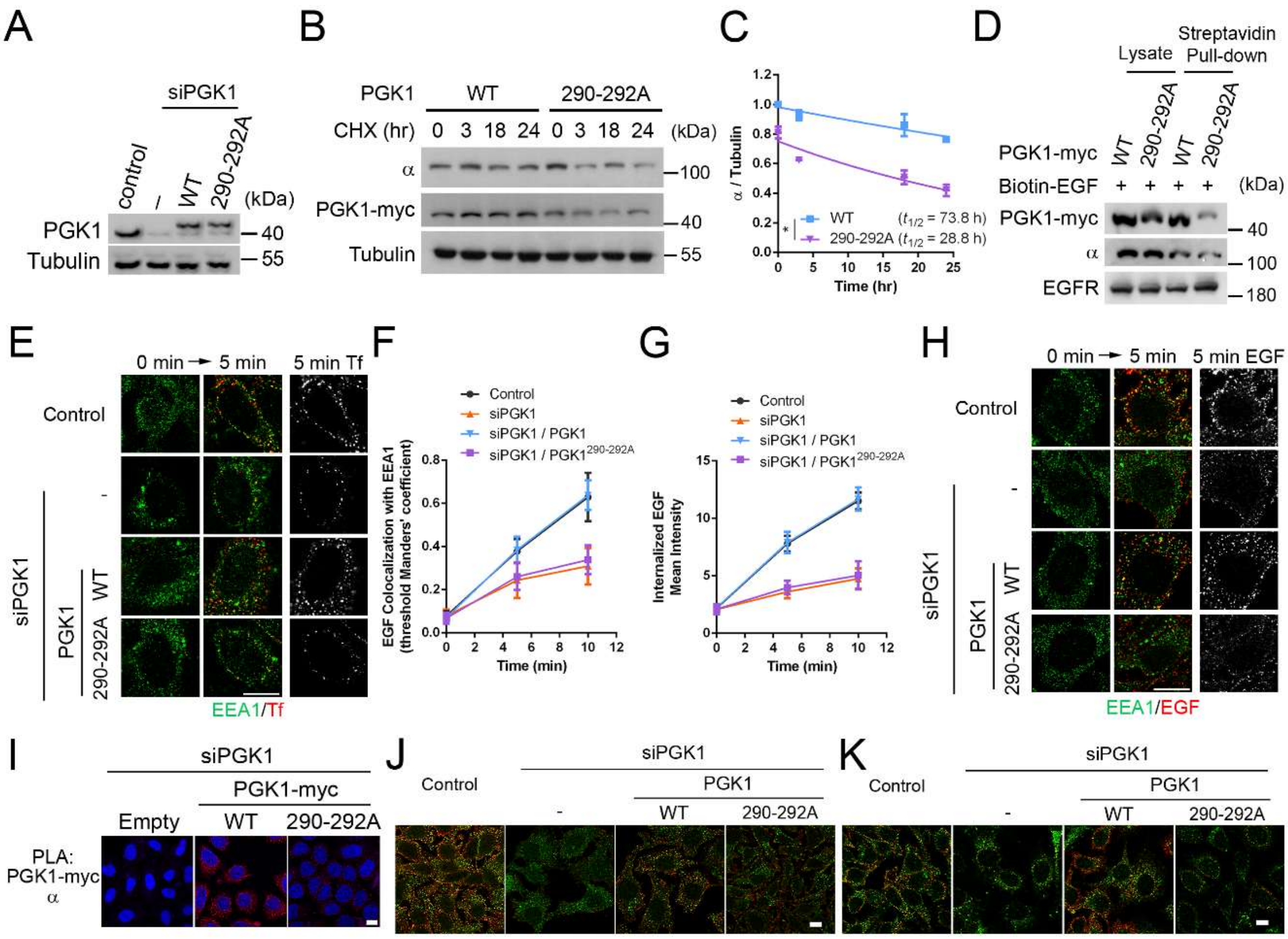

Appendix Figure S6. α subunit interacts with PGK1 via the DKF motif. Data are shown as the means ± SDs. *P<0.05

(A) Immunoblot analysis of HEK293T cell lysates to examine the rescue effects of PGK1 and PGK1^{T378P} in siPGK1 cells (three independent biological replicates).

(B) Time course analysis of α degradation upon CHX treatment, examining the effect of PGK1 and PGK1$^{290\text{-}292A}$.

(C) Protein levels of α were normalized to Tubulin and plotted against time (three independent biological replicates). Curves were fitted using a one-phase decay non-linear regression model. The calculated half-life ($t_{1/2}$) is 73.8 h for the PGK1 and 28.8 h for PGK1$^{290\text{-}292A}$. Curves were compared using an extra sum-of-squares F-test (P=0.0036).

(D) Endogenous surface EGFR associated with endogenous α and myc-tagged PGK1 or PGK1$^{290\text{-}292A}$ in siPGK1-treated cells (three independent biological replicates).

(E) Representative image of Tf (red) colocalized with EEA1 (green); scale bar, 10 µm.

(F) Colocalization of EGF with EEA1, expressed as thresholded Manders' coefficient, showing the effects of PGK1 and PGK1$^{290\text{-}292A}$ in siPGK1 cells at 0, 5, and 10 minutes

(n=15 cells of one representative experiment from two independent biological replicates).

(G) Mean intensity of internalized Tf showing the effects of PGK1 and PGK1$^{290\text{-}292A}$ in siPGK1 cells at 0, 5, and 10 minutes (n=15 cells of one representative experiment from two independent biological replicates).

(H) Representative image of EGF (red) colocalized with EEA1 (green); scale bar, 10 μm.

(I) Representative image with a larger field of view corresponding to Fig. 6C.

(J) Representative image with a larger field of view corresponding to Appendix Fig. S6E.

(K) Representative image with a larger field of view corresponding to Appendix Fig. S6H.

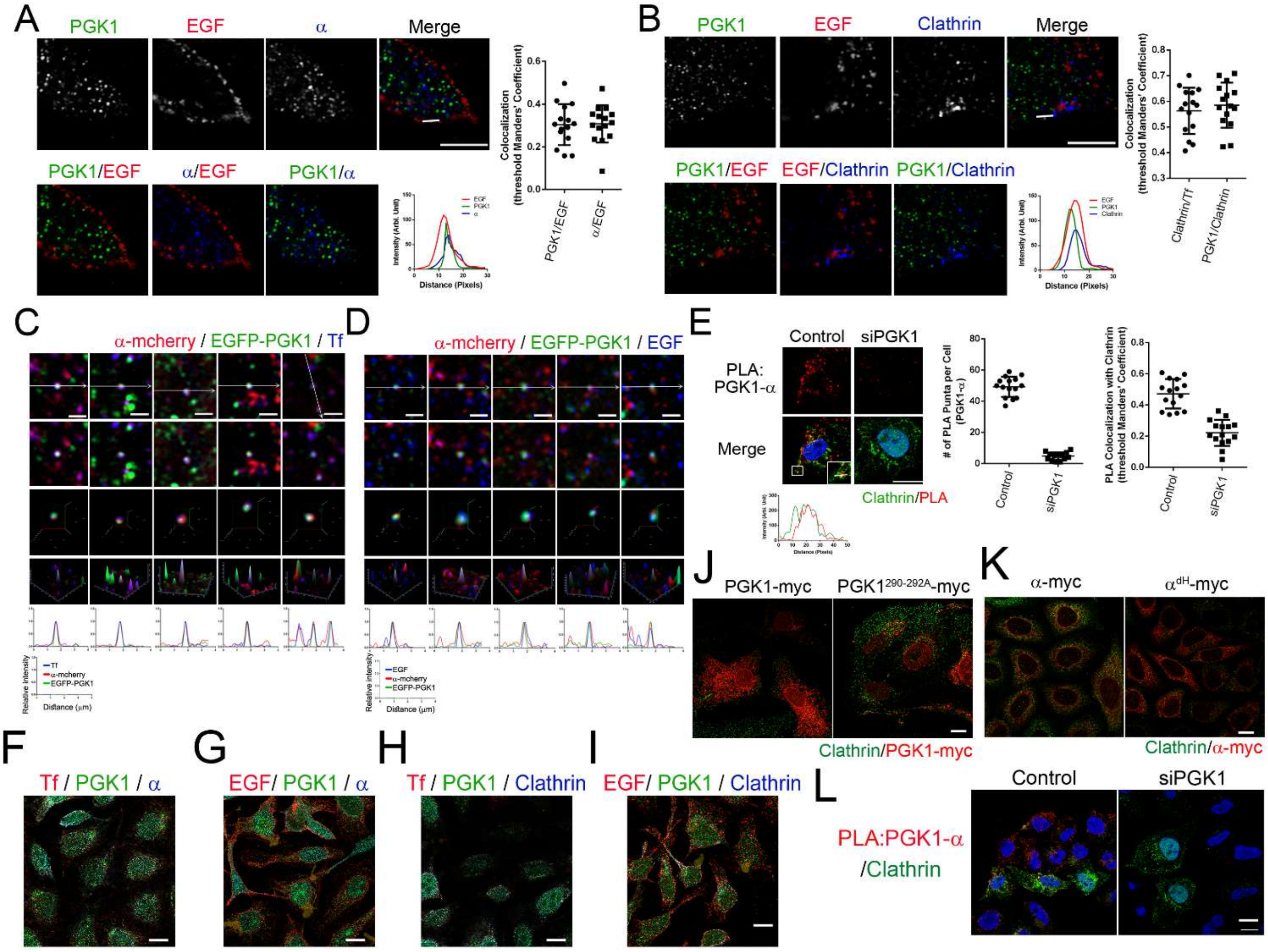

Appendix Figure S7. PGK1 colocalizes with clathrin and the AP2 complex during protein endocytosis. Data are shown as the means ± SDs.

(A) Super-resolution confocal image after pre-lysis extraction showing colocalization of PGK1, EGF, and α after 2 minutes of internalization; scale bar, 10 μm. Intensity profiles show the relative fluorescence intensities along the white lines in the merge panels. Quantification (right) of PGK1 with EGF and of α with EGF colocalization, expressed as thresholded Manders' coefficient (n=15 cells of one representative experiment from two independent biological replicates).

(B) Super-resolution confocal image after pre-lysis extraction showing colocalization of PGK1, EGF, and clathrin after 2 minutes of internalization; scale bar, 10 μm. Intensity profiles show the relative fluorescence intensities along the white lines in the merge panels. Quantification (right) of PGK1 with clathrin and of clathrin with EGF colocalization, expressed as thresholded Manders' coefficient; (n=15 cells of one representative experiment from two independent biological replicates).

(C) Colocalization analysis optimized by the prelysis method demonstrating that α-mCherry, EGFP-PGK1, and Tf colocalize in cells; scale bar, 1 μm. Intensity profiles show the relative fluorescence intensities along the white lines in the first panels.

(D) Colocalization analysis optimized by the prelysis method demonstrating that α-mCherry, EGFP-PGK1, and EGF colocalize in cells; scale bar, 1 μm. Intensity profiles show the relative fluorescence intensities along the white lines in the first panels.

(E) PLA analysis showing PGK1—α PLA puncta and thresholded Manders' coefficient colocalization with clathrin (GFPspark-CTLB) (n=15 cells of one representative experiment from two independent biological replicates); scale bar, 10 μm. Insets show the enlarged regions of colocalization events, with line profiles indicating the relative fluorescence intensities along the white line.

(F) Representative image with a larger field of view corresponding to Fig. 7C.

(G) Representative image with a larger field of view corresponding to Appendix Fig. S7A.

(H) Representative image with a larger field of view corresponding to Fig. 7D.

(I) Representative image with a larger field of view corresponding to Appendix Fig. S7B.

(J) Representative image with a larger field of view corresponding to Fig. 7E.

(K) Representative image with a larger field of view corresponding to Fig. 7F.

(L) Representative image with a larger field of view corresponding to Appendix Fig. S7E.

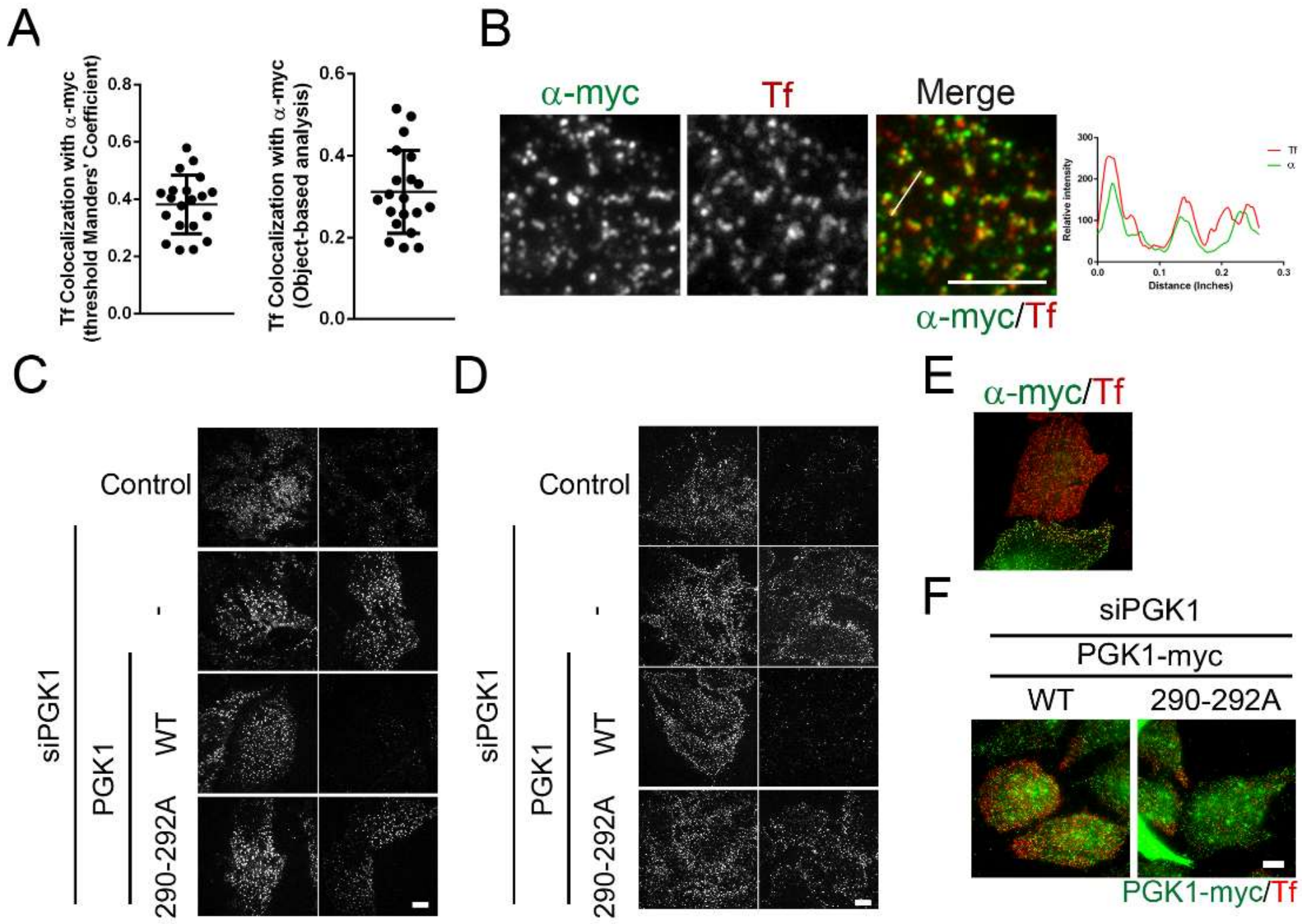

Appendix Figure S8. TIRF imaging demonstrated that α subunit recognizes Tf at the plasma membrane. Data are shown as the means ± SDs.

(A) Quantitative results of Tf colocalization with α-myc, expressed in thresholded Manders' coefficient (left) and object-based analysis (right) (n = 20 cells of one representative experiment from two independent biological replicates).

(B) TIRF imaging representing the colocalization of Tf and α-myc; scale bar, 5 μm. Intensity profiles show the relative fluorescence intensities along the white lines in the merge panels.

(C) Representative image with a larger field of view corresponding to Fig. 8B.

(D) Representative image with a larger field of view corresponding to Fig. 8D.

(E) Representative image with a larger field of view corresponding to Appendix Fig. S8B.

(F) Representative image with a larger field of view corresponding to Fig. 8F.
