# Supplementary material for: Phosphoglycerate kinase 1 cooperates with the AP2 complex to promote endocytosis
Source: EMBO Rep. 2026 Oct 1;27(21):6447–63. doi: 10.1038/s44319-026-00948-5 (PMC13743783; doi:10.1038/s44319-026-00948-5)
Supplement: Supplementary file 2 [file 44319_2026_948_MOESM2_ESM.pdf]

EMBO reports

Phosphoglycerate kinase 1 cooperates with the AP2 complex to promote endocytosis

Jia-Wei Hsu, Shao-Ling Chu, Yu-Tzu Chang, Che-Yu Hung, and Chia-Jung Yu

Corresponding author(s): Jia-Wei Hsu (jwhsu@ntu.edu.tw)

Review Timeline:

Submission Date:	7th Nov 25
Editorial Decision:	17th Dec 25
Revision Received:	17th Mar 26
Editorial Decision:	18th May 26
Revision Received:	18th Jun 26
Editorial Decision:	13th Aug 26
Revision Received:	21st Aug 26
Accepted:	10th Sep 26

Editor: Kurt Weir / Martina Rembold

Transaction Report:

(Note: With the exception of the correction of typographical or spelling errors that could be a source of ambiguity, letters and reports are not edited. Depending on transfer agreements, referee reports obtained elsewhere may or may not be included in this compilation. Referee reports are anonymous unless the Referee chooses to sign their reports.)

1st Editorial Decision **17th Dec 2025**

Dear Prof. Hsu

Thank you for the submission of your research manuscript to our journal. We have now received the full set of referee reports that is copied below.

As you have seen, the referees acknowledge that the findings are interesting and that the conclusions are overall supported by the data presented but they also raise a number of concerns and have suggestions how to further strengthen the data, particularly greater characterization of the PGK1-AP2 interaction including its specificity over other AP complexes or adaptins, and the interaction of PGK1 with the different AP2 subunits.

Given these constructive comments, we would like to invite you to revise your manuscript with the understanding that the referee concerns (as detailed above and in their reports) must be fully addressed and their suggestions taken on board. Please address all referee concerns in a complete point-by-point response. Acceptance of the manuscript will depend on a positive outcome of a second round of review. It is EMBO Reports policy to allow a single round of revision only and acceptance or rejection of the manuscript will therefore depend on the completeness of your responses included in the next, final version of the manuscript.

We realize that it is difficult to revise to a specific deadline. In the interest of protecting the conceptual advance provided by the work, we recommend a revision within 3 months (March 17th). Please discuss the revision progress ahead of this time with the editor if you require more time to complete the revisions.

I am also happy to discuss the revision further via e-mail or a video call, if you wish.

You can either publish the study as a short report or as a full article. For short reports, the revised manuscript should not exceed 27,000 characters (including spaces but excluding materials & methods and references) and 5 main plus 5 expanded view figures. The results and discussion sections must further be combined, which will help to shorten the manuscript text by eliminating some redundancy that is inevitable when discussing the same experiments twice. For a normal article there are no length limitations, but it should have more than 5 main figures and the results and discussion sections must be separate. In both cases, the entire materials and methods must be included in the main manuscript file.

================
IMPORTANT NOTE:

We perform an initial quality control of all revised manuscripts before re-review. Your manuscript will FAIL this control and the handling will be delayed IN CASE the following APPLIES:

1) A data availability section providing access to data deposited in public databases is missing. If you have not deposited any data, please add a sentence to the data availability section that explains that.

2) Your manuscript contains statistics and error bars based on n=2. Please use scatter blots in these cases. No statistics should be calculated if n=2.

When submitting your revised manuscript, please carefully review the instructions that follow below. Failure to include requested items will delay the evaluation of your revision.

================

When submitting your revised manuscript, we will require:

1) a .docx formatted version of the manuscript text (including legends for main figures, EV figures and tables). Please make sure that the changes are highlighted to be clearly visible.

2) individual production quality figure files as .eps, .tif, .jpg (one file per figure).
Please download our Figure Preparation Guidelines (figure preparation pdf) from our Author Guidelines pages https://www.embopress.org/page/journal/14693178/authorguide for more info on how to prepare your figures.

3) a .docx formatted letter INCLUDING the reviewers' reports and your detailed point-by-point responses to their comments. As part of the EMBO Press transparent editorial process, the point-by-point response is part of the Review Process File (RPF), which will be published alongside your paper.

4) a complete author checklist, which you can download from our author guidelines

(<https://www.embopress.org/page/journal/14693178/authorguide>). Please insert information in the checklist that is also reflected in the manuscript. The completed author checklist will also be part of the RPF.

5) Please note that all corresponding authors are required to supply an ORCID ID for their name upon submission of a revised manuscript (<https://orcid.org/>). Please find instructions on how to link your ORCID ID to your account in our manuscript tracking system in our Author guidelines
(<https://www.embopress.org/page/journal/14693178/authorguide#authorshipguidelines>)

6) We replaced Supplementary Information with Expanded View (EV) Figures and Tables that are collapsible/expandable online. A maximum of 5 EV Figures can be typeset. EV Figures should be cited as 'Figure EV1, Figure EV2" etc... in the text and their respective legends should be included in the main text after the legends of regular figures.

- For the figures that you do NOT wish to display as Expanded View figures, they should be bundled together with their legends in a single PDF file called *Appendix*, which should start with a short Table of Content. Appendix figures should be referred to in the main text as: "Appendix Figure S1, Appendix Figure S2" etc. See detailed instructions regarding expanded view here:
<https://www.embopress.org/page/journal/14693178/authorguide#expandedview>

- Additional Tables/Datasets should be labeled and referred to as Table EV1, Dataset EV1, etc. Legends have to be provided in a separate tab in case of .xls files. Alternatively, the legend can be supplied as a separate text file (README) and zipped together with the Table/Dataset file.

7) Before submitting your revision, primary datasets (and computer code, where appropriate) produced in this study need to be deposited in an appropriate public database (see <
https://www.embopress.org/page/journal/14693178/authorguide#dataavailability>).

Please remember to provide a reviewer password if the datasets are not yet public.

The accession numbers and database should be listed in a formal "Data Availability " section (placed after Materials & Method) that follows the model below (see also < https://www.embopress.org/page/journal/14693178/authorguide#dataavailability>). Please note that the Data Availability Section is restricted to new primary data that are part of this study.

Data availability

The datasets (and computer code) produced in this study are available in the following databases:

- RNA-Seq data: Gene Expression Omnibus GSE46843 (https://www.ncbi.nlm.nih.gov/geo/query/acc.cgi?acc=GSE46843)
- [data type]: [name of the resource] [accession number/identifier/doi] ([URL or identifiers.org/DATABASE:ACCESSION])

*** Note - All links should resolve to a page where the data can be accessed. ***

8) At EMBO Press we ask authors to provide source data for the main manuscript figures. You will receive a separate email with instructions for providing source data with your revised manuscript, including how to upload and organize the files.

Additional information on source data and instruction on how to label the files are available
<https://www.embopress.org/page/journal/14693178/authorguide#sourcedata>

9) The journal requires a statement specifying whether or not authors have competing interests (defined as all potential or actual interests that could be perceived to influence the presentation or interpretation of an article). In case of competing interests, this must be specified in your disclosure statement. Further information: https://www.embopress.org/competing-interests

10) Figure legends and data quantification:
The following points must be specified in each figure legend:

- the name of the statistical test used to generate error bars and P values,
- the EXACT p-values,
- the number (n) of independent experiments (please specify technical or biological replicates) underlying each data point,
- the nature of the bars and error bars (s.d., s.e.m.)

- If the data are obtained from n {less than or equal to} 5, show the individual data points in addition to the SD or SEM.
- If the data are obtained from n {less than or equal to} 2, use scatter blots showing the individual data points.

Discussion of statistical methodology can be reported in the materials and methods section, but figure legends should contain a basic description of n, P and the test applied.

See also the guidelines for figure legend preparation:
https://www.embopress.org/page/journal/14693178/authorguide#figureformat

- Please also include scale bars in all microscopy images and define their size in the legend, not the image.

11) Our journal encourages inclusion of *data citations in the reference list* to directly cite datasets that were re-used and obtained from public databases. Data citations in the article text are distinct from normal bibliographical citations and should directly link to the database records from which the data can be accessed. In the main text, data citations are formatted as follows: "Data ref: Smith et al, 2001" or "Data ref: NCBI Sequence Read Archive PRJNA342805, 2017". In the Reference list, data citations must be labeled with "[DATASET]". A data reference must provide the database name, accession number/identifiers and a resolvable link to the landing page from which the data can be accessed at the end of the reference. Further instructions are available at <https://www.embopress.org/page/journal/14693178/authorguide#referencesformat>.

12) All Materials and Methods need to be described in the main text using our 'Structured Methods' format. According to this format, the Methods section includes a Reagents and Tools Table (listing key reagents, experimental models, software and relevant equipment and including their sources and relevant identifiers) followed by a Methods and Protocols section describing the methods, ideally using a step-by-step protocol format. The aim is to facilitate adoption of the methodologies across labs. Please download and fill our Reagents and Tools Table template (.docx), which you can find in our author guidelines: https://www.embopress.org/page/journal/14693178/authorguide#structuredmethods.
When submitting your revised manuscript, please do not include the Reagents and Tools Table in the Methods section of the manuscript but upload it as a separate file choosing the file type "Reagent Table".
An example of a Method paper with Structured Methods can be found here:
https://www.embopress.org/doi/10.15252/msb.20178071.

13) As part of the EMBO publication's Transparent Editorial Process, EMBO Reports publishes online a Review Process File to accompany accepted manuscripts. This File will be published in conjunction with your paper and will include the referee reports, your point-by-point response and all pertinent correspondence relating to the manuscript.

You are able to opt out of this by letting the editorial office know (emboreports@embo.org). If you do opt out, the Review Process File link will point to the following statement: "No Review Process File is available with this article, as the authors have chosen not to make the review process public in this case."

We would also welcome the submission of cover suggestions, or motifs to be used by our Graphics Illustrator in designing a cover.

I look forward to seeing a revised form of your manuscript when it is ready and please let me know if you have questions or comments regarding the revision.

Yours sincerely,

Kurt Weir
Editor
EMBO Reports

Referee #1:

To the authors

Review of the manuscript: Phosphoglycerate kinase 1 cooperates with AP-2 complex to promote endocytosis Authors: Jia-Wei Hsu, Shao-Ling Chu, Yu-Tzu Chang, Che-Yu Hung, and Chia-Jung Yu Manuscript Number: EMBOR-2025-63143-T

The authors have shown previously that the Phosphoglycerate kinase 1, PGK1, binds the di-leucine based sorting motif sequence of EGFR and that this interaction is required for EGFR lysosomal degradation. In this manuscript they describe a follow up study, in which they want to identify the molecular mechanism by which PGK1 mediates this EGFR pathway.

1.) The authors do not use the established nomenclature for AP2 adaptin subunits. The genes are named AP2A, AP2B, AP2M and AP2S. The adaptin protein nomenclature is α, β2, μ2 and σ2. The authors mix the gene and protein nomenclature in their manuscript and this has to be corrected.

2.) The authors write that they tested endosomal membranes for proteins binding to PGK1 by a co-IP. However, they describe

only cell lysis in the materials and methods section and no subcellular fractionation procedure. Moreover, a major hit in their mass spectrometric analysis of the pulldown fraction is AP2, which binds to the plasma membrane and a second fraction is found in the cytosol. AP2 is only found in mutants, in which the AP2 cytoplasm-plasma membrane recycling is impaired. This description of experiments doesn't meet the scientific standards.

3.) The mass spec experiments did not reveal other proteins involved in the AP2 endocytic pathway as PGK1 binding proteins, nor to the AP2 related AP-complexes AP1 and AP3. However, only AP2 α and β2 were identified and not the additional μ2 and σ2 adaptins. This demonstrates that the mass spec experiment was not able to identify all relevant proteins. Therefore, in all cell lysate protein isolation experiments negative controls have to be included to verify the specificity of the AP2-PGK1 interaction. Suitable antibodies are commercially available for numerous AP2 CCV coat proteins. This is also the case for adaptins of the AP1 and AP3 complexes.

4.) The authors measured endocytosis of TfR and EGFR. Previously, the authors showed that EGFR endocytosis and subsequent lysosomal degradation requires PGK1. However, in Pascolutti et al. 2019 Cell Reports 27, 3049-3061 it has been shown, that AP2 is not involved in the clathrin-mediated endocytosis of EGFR which leads to its lysosomal degradation. Instead, the involvement of AP2 CCV directs EGFR in an endosome-plasma membrane recycling pathway. Therefore, the authors are able to discriminate between a possible function of PGK1-AP2 binding versus PGK1-EGFR binding.
5.) The authors identified PGK1 binding to the α, but also to the μ2 and σ2 adaptins. This raises concerns about the specificity and thus functional relevance of these interactions. This is not further verified and only the α-adaptin binding has been analysed, which led to the identification of the α-adaptin ‚hinge' domain as the PGK1 binding domain. These data and those of the subsequent experiments (my point 6) raise concerns about the quality of the data. Their significance has to be demonstrated in follow up experiments.

6.) The authors are aware that α-adaptin binds CCV coat proteins via a ‚DKF' amino acid motif. PGK1 also contains such a motif and the authors show that this motif mediates the binding to the α-adaptin ‚hinge' domain. However, the authors seem not to be aware that α-adaptin does not bind ‚DKF'-motif proteins via its ‚hinge' domain, but via its ‚ear' domain. Crystal structures of this interaction have been published. This discrepancy between their data and the literature is not even being discussed. Therefore, a sequence motif within the ‚hinge' domain has to be identified, which binds the ‚DKF' PGK1 sequence.

7.) The authors do not discuss any structural data, but show a superficial cartoon. I looked myself, whether the ‚DKF' sequence in PGK1 is at a position, which enables binding to other proteins. It is indeed in a surface loop. Now the binding ‚hinge' motif has to be identified. This positioning of PGK1 to the ‚hinge' domain should indicate, which PGK1 domains are able to bind to μ2 and σ2 domains.

These points have to be addressed before the manuscript is suitable for publication in EMBO Reports. However, this manuscript can be much improved by additional data and discussions of the AP2 literature.

Referee #2:

The authors demonstrate that the interaction between PGK1 and AP-2 is essential for the efficient endocytosis of specific cargo proteins, such as TfnR and EGFR. They further map the interaction interfaces between PGK1 and AP-2 that are required for their roles in the endocytic pathway. Together, these findings reveal a previously unrecognized regulatory layer in clathrin-mediated endocytosis. However, several concerns remain and should be addressed.

1. The data presented in this study support a role for the PGK1-AP-2 interaction in clathrin-mediated endocytosis; however, the authors do not provide evidence for downstream cargo-sorting events following internalization, despite PGK1's ability to recognize TfnR and EGFR sorting signals. The authors should therefore temper their statements regarding cargo sorting throughout the manuscript.

2. The quality of immunofluorescent data in the first half of the manuscript were not sufficient to support their statement (Fig. 4A; 7C; EV4A; EV5D, E; EV7A, B). The cytosol washout step, together with the use of super-resolution confocal microscopy and TIRF imaging, substantially improved the quality of the immunofluorescence data. The authors may consider presenting only these higher-quality datasets and integrating them into the earlier sections of the manuscript.

3. The authors should provide the raw MS analysis data, including the associated statistical analyses, to substantiate their rationale for focusing on AP-2α.

4. For the CHX-treatment protein degradation assay, the analysis should be normalized to the 0-h CHX time point for each condition to accurately assess degradation rates.

5. It has been reported that the interaction between AP-2 and PIP_2 induces the open conformation of AP-2, which is also required for its interaction with clathrin (PMID: 25061211). It is therefore surprising that AP-2α-dH still reduces the interaction between AP-2 and clathrin, despite retaining the appendage domain (Fig. 7F). Moreover, the data in Fig. 4F indicate that PIP_2

binding enhances the affinity between AP-2 and PGK1, suggesting that the AP-2-PIP_2 interaction may also be necessary for PGK1-AP-2 association. These observations are novel and potentially significant for the field, and the authors may need to discuss these points in greater depth.

Minor comments:

1. The WB blot data were missing to demonstrate the protein knockdown and reconstitution efficiency.

2. Quantification data were missing for some WB blots and IF imaging to support the statements (Fig. 2F, G; EV2C; EV4D; EV5C).

Referee #3:

In this study, Chu et al aimed to elucidate a novel function of PGK1 in regulating clathrin-mediated endocytosis (CME) beyond its well-established roles in glycolysis. Biochemical assays and fluorescent microscopy imaging have been utilized to show that PGK1 interacts with and stabilizes AP2 complex, which is correlated with its cargo binding capacity but independent of its kinase activity. Overall, the novel function of PGK1 in CME regulation is intriguing to cell biologists; however, several issues remain in the mechanistic elucidation that require further validation.

Major points:
1. The authors claim that PGK1 mainly binds to the α subunit of the AP-2 complex, which functions as a bridge linking PGK1 with other subunits of the AP2 complex. To comprehensively validate this conclusion, the below concerns about the biochemical assays of PGK1-AP2 interactions should be addressed:
a) All subunits of the endogenous AP-2 complex in Co-IP experiments should be examined by WB. For example, AP2-β and AP2-σ should be examined in Fig. 2B and Fig. EV2C.
b) AP2-σ should be examined in Fig. 3A,B.
c) Based on the results shown in Fig. 2F,G, it looks that PGK1 binds stronger to AP2 complex. However, it is difficult to directly conclude that AP2-α act as a bridge linking PGK1 and AP2 complex. It would be more convincing if the authors can show that: i) siRNA knockdown of AP2-β or AP2-µ does not affect PGK1 interaction with AP2-α; ii) replace AP2-α with AP2-β in the GST-pulldown assay shown in Fig. 2F,G does not enhance PGK1 binding to AP2-µ and AP2-σ.
d) If PGK1 can bind to AP2 complex, why AP2-µ and AP2-σ was not detected in the MassSpec result of PGK1-interacting proteins that are listed in Table EV1?
e) FxDxF or DxF motifs are classical AP2 α-AD interacting motifs, how to explain that PGK1 barely binds to α-AD domain? Although the role of DKF in mediating the interaction between PGK1 and AP2 is well validated, the authors could employ AlphaFold prediction to gain deeper insights into their interactions.

2. The effects shown in figure 3C-F are too weak to support the role of PGK1 in regulating the stability of AP-2 complex. Stronger experimental data are needed, please repeat that, and detect the ubiquitination levels of AP2-α under PGK1 knockdown.
3. The Confocal Microscopy results in quantifying the colocalizations of PGK1, AP2-α and cargos are not convincing. Based on the representative images shown in Figs. 4, EV4 and EV5, no obvious colocalizations were observed between PGK1 and EGF or Tf; however, the authors' quantification analysis showed high overlap coefficients. The discrepancy raises concerns about the accuracy of the method. In addition, it is not clear how the authors chose where and what direction to draw the lines as shown in Fig. 4A and Fig. EV5D,E. Thus, whether these fluorescent curves are representative is in question.

Minor point:
1. The authors should explain in Methods why they chose Manders' Overlap Coefficient rather than Pearson's Correlation Coefficient in quantifying the colocalization signals. Moreover, details on how the authors conduct the Manders' Overlap Coefficient analysis should be included. How do the authors make sure that the background subtraction and threshold setting won't affect the result interpretation.

RESPONSES (EMBOR-2025-63143-T)

We thank the reviewers for the overall positive assessment of our work. Each has also provided comments that suggest how we can further improve our study and its presentation. Taking them to heart, we have performed many additional experiments, which are shown in revised figures or in new figures. They are described below as part of the point-by-point response to all comments of the last review. Moreover, to help detect the many changes in the revised manuscript, we have highlighted the text in blue to indicate where changes are made.

Referee 1

Review of the manuscript: Phosphoglycerate kinase 1 cooperates with AP-2 complex to promote endocytosis Authors: Jia-Wei Hsu, Shao-Ling Chu, Yu-Tzu Chang, Che-Yu Hung, and Chia-Jung Yu Manuscript Number: EMBOR-2025-63143-T

The authors have shown previously that the Phosphoglycerate kinase 1, PGK1, binds the di-leucine based sorting motif sequence of EGFR and that this interaction is required for EGFR lysosomal degradation. In this manuscript they describe a follow up study, in which they want to identify the molecular mechanism by which PGK1 mediates this EGFR pathway.

1.) The authors do not use the established nomenclature for AP2 adaptin subunits. The genes are named AP2A, AP2B, AP2M and AP2S. The adaptin protein nomenclature is α, β2, µ2 and σ2. The authors mix the gene and protein nomenclature in their manuscript and this has to be corrected.

We thank the reviewer for this important clarification regarding the nomenclature issue. We have thoroughly revised the manuscript, including all text, figures, and figure legends, to strictly adhere to the established nomenclature.

2.) The authors write that they tested endosomal membranes for proteins binding to PGK1 by a co-IP. However, they describe only cell lysis in the materials and methods section and no subcellular fractionation procedure. Moreover, a major hit in their mass spectrometric analysis of the pulldown fraction is AP2, which binds to the plasma membrane and a second fraction is found in the cytosol. AP2 is only found in mutants, in which the AP2 cytoplasm-plasma membrane recycling is impaired. This description of experiments doesn't meet the scientific standards.

We thank the reviewer for pointing out the ambiguity in our original Materials and Methods regarding our protein isolation procedures. Our initial mass spectrometry screening was performed using total cell lysates to provide a comprehensive view of the PGK1 interactome involved in the endocytic pathway. To clarify this, we have updated the Materials and Methods section to include the specific lysis conditions and centrifugation steps used (15,000x*g* for 10 min at 4°C) to generate the clarified lysates used for Co-IP. We also agree with the reviewer that AP2 complex is the core adaptor for clathrin-mediated endocytosis and is primarily localized to the plasma membrane and the cytosol. The identification of AP2 complex as a major interactor in our MS analysis is consistent with PGK1 playing a role in the initiation or stabilization of endocytosis at the plasma membrane, rather than exclusively at later endosomal stages.

3.) The mass spec experiments did not reveal other proteins involved in the AP2 endocytic pathway as PGK1 binding proteins, nor to the AP2 related AP-complexes AP1 and AP3. However, only AP2 α and β2 were identified and not the additional μ2 and σ2 adaptins. This demonstrates that the mass spec experiment was not able to identify all relevant proteins. Therefore, in all cell lysate protein isolation experiments negative controls have to be included to verify the specificity of the AP2-PGK1 interaction. Suitable antibodies are commercially available for numerous AP2 CCV coat proteins. This is also the case for adaptins of the AP1 and AP3 complexes.

We thank the reviewer for this comment. In our label free single run proteomic experiment, we detected multiple components of the AP2 complex (α, β2, σ2, and μ2) as well as the AP1 complex γ subunit. Because replicate based quantitative statistics were not applicable for this dataset, we evaluated potential PGK1 interacting proteins using the Normalized PSM (%), a semi quantitative indicator of relative abundance. We then prioritized the top 100 proteins ranked by Normalized PSM (%) and focused on vesicular trafficking-related candidates for downstream validation. Within this high confidence group, α subunit of AP2 complex emerged as a prominent target, guiding our subsequent experiments. The PGK1-interacting proteins identified by label free proteomic analysis in this study have been provided in the revised Table EV1. Our finding is consistent with a recent report showing that a PGK1 TurboID dataset identified α subunit as one of the major hits in their screening results (Camara *et al*, 2025). To address concerns regarding specificity, we have now included additional controls in our biochemical assays. Specifically, we performed immunoprecipitation experiments using antibodies against AP1 and AP3 adaptins and observed weak interactions with PGK1 compared to the association detected with the all four subunits, α, μ2, σ2 and β2, of AP2 complex under identical experimental conditions (Figure 2A). Nevertheless, unlike the AP2 complex, the AP1 and AP3 components remained stable in siPGK1-treated cells (Figure 3A and 3B). These findings suggest that the PGK1-AP2 interaction is preferential and functionally relevant. Nevertheless, the detectable but modest association with AP1 or AP3 raises the possibility that PGK1 may also participate in additional transport pathways mediated by these adaptors, which needs further investigation. Collectively, these results further clarify the PGK1-AP2 interaction.

Ref

Camara GA, Yokoo S, Granato DC, Simabuco FM, Ribeiro-Filho HV, Melo RM, Pauletti BA, Nascimento Filho EG, Domingues RR, Paes Leme AF (2025) Mapping the Interactome of OSCC Prognostic-Associated Proteins NDRG1 and PGK1 Through Proximity Labeling Using TurboID. J Proteome Res 24: 2741-2756

4.) The authors measured endocytosis of TfR and EGFR. Previously, the authors showed that EGFR endocytosis and subsequent lysosomal degradation requires PGK1. However, in Pascolutti et al. 2019 Cell Reports 27, 3049-3061 it has been shown, that AP2 is not involved in the clathrin-mediated endocytosis of EGFR which leads to its lysosomal degradation. Instead, the involvement of AP2 CCV directs EGFR in an endosome-plasma membrane recycling pathway. Therefore, the authors are able to discriminate between a possible function of PGK1-AP2 binding versus PGK1-EGFR binding.

We thank the reviewer for raising the significant point. We have significantly revised the Discussion to address the literature (Pascolutti et al., 2019) and clarify the distinct roles of PGK1 during EGFR endocytic transport

pathway. In short, our previous work showed that PGK1-EGFR interaction on the dileucine sorting motif on the juxtamembrane region of EGFR leading to lysosomal degradation of EGFR. In addition, our current data demonstrates that PGK1-AP2 interaction is important for the EGFR internalization through the direct recognition of tyrosine-based and dileucine sorting motif on the cytoplasmic tail of EGFR. The different sorting motif might show distinct pathway for the sorting process. In this case, PGK1 appears to play distinct roles in internalization and lysosomal transport by separate molecular mechanisms, for example, binding to different sorting motifs or switching to different interacting partners. A detailed exploration of these intricate regulatory pathways is necessary, but such a comprehensive analysis exceeds the limitations of our current study and will be pursued in subsequent investigations. We have revised our manuscript to clarify this concern in the Discussion section.

5.) The authors identified PGK1 binding to the α, but also to the μ2 and σ2 adaptins. This raises concerns about the specificity and thus functional relevance of these interactions. This is not further verified and only the α-adaptin binding has been analysed, which led to the identification of the α-adaptin ,hinge' domain as the PGK1 binding domain. These data and those of the subsequent experiments (my point 6) raise concerns about the quality of the data. Their significance has to be demonstrated in follow up experiments.

We thank the reviewer for raising this important concern. In our study, PGK1 was detected in association with multiple AP2 subunits, including α, μ2, and σ2 subunits. We agree that demonstrating specificity and functional relevance is essential. Importantly, our subsequent validation experiments focused on the α subunit because it exhibited the most robust and reproducible interaction signal across independent assays. Domain-mapping analysis further identified the hinge region of α subunit is required for PGK1 binding, providing mechanistic insight into the interaction. In addition, we note that subunits of AP2 complex are structurally and spatially interconnected. Therefore, detection of μ2 and σ2 in association with PGK1 is consistent with PGK1 engaging the assembled AP2 complex rather than interacting independently and equivalently with each subunit. To clarify this point and address specificity, we have now performed additional experiments, including co-immunoprecipitation and interaction assays, which support a preferential and functionally relevant interaction between PGK1 and AP2 complex. The interaction with other AP2 subunits will be likely mediated by the binding of PGK1 to the alpha subunit but not beta subunit, which acts as the primary bridge, stabilizing the overall complex (Figure 2E and 2F and Appendix Figure S2C and S2D). To further understand the AP2-PGK1 interaction, we performed structural prediction of the AP2-PGK1 complex using AlphaFold to explore potential interaction interfaces at the molecular level (Response Figure 1, shown below). The predicted models suggested that PGK1 associated with the peripheral region of the AP2 complex, positioning near the core region, consistent with a role as an accessory factor that engages the assembled complex. However, we realized important limitations of this approach, as the overall confidence scores in AlphaFold predictions for the complex were not reliable (pTM=0.6, iPTM=0.62). We considered that the modeling was conducted in the absence of a membrane environment, lipid components (e.g., PI(4,5)P_2), and the conformational dynamics associated with AP2 activation. Because AP2 undergoes substantial structural rearrangement upon membrane recruitment and cargo engagement (*Partlow et al, 2022*), predictions generated under soluble conditions may not accurately reflect the physiologically relevant conformation or stability of the complex. Therefore, the model should be considered a hypothetical framework for potential contact interfaces that will require further experimental structural validation under membrane-mimetic conditions.

Ref

Partlow EA, Cannon KS, Hollopeter G, Baker RW (2022) Structural basis of an endocytic checkpoint that primes the AP2 clathrin adaptor for cargo internalization. *Nat Struct Mol Biol* 29: 339-347

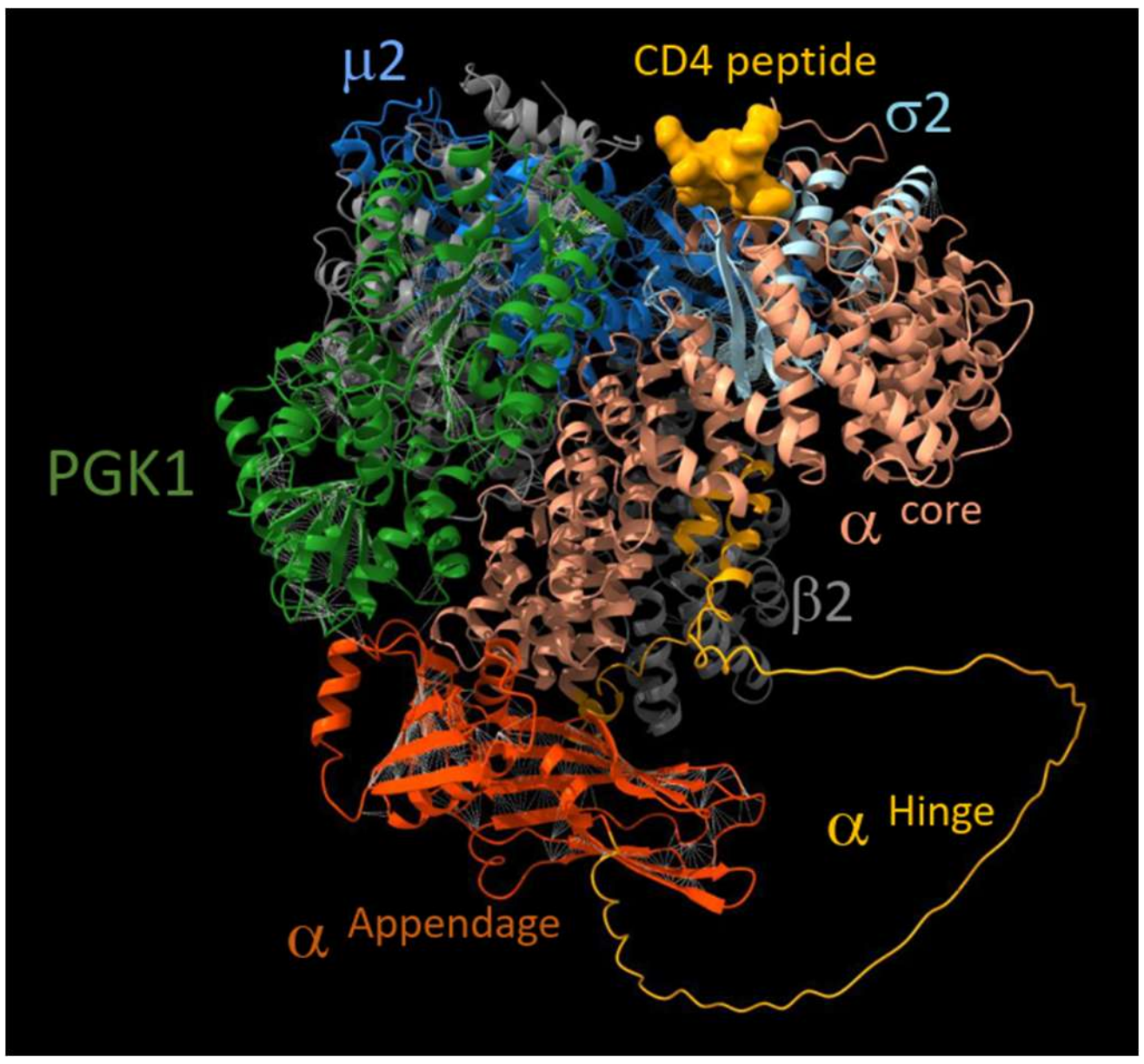

Response Figure 1. Structural prediction of PGK1-AP2 complex

6.) The authors are aware that α-adaptin binds CCV coat proteins via a ‚DKF' amino acid motif. PGK1 also contains such a motif and the authors show that this motif mediates the binding to the α-adaptin ‚hinge' domain. However, the authors seem not to be aware that α-adaptin does not bind ‚DKF'-motif proteins via its ‚hinge' domain, but via its ‚ear' domain. Crystal structures of this interaction have been published. This discrepancy between their data and the literature is not even being discussed. Therefore, a sequence motif within the ‚hinge' domain has to be identified, which binds the ‚DKF' PGK1 sequence.

We thank the reviewer for highlighting this important point. We are aware that canonical DKF motifs typically bind to the ear domain of α subunit, and that structural studies have defined this interaction interface. Our data indeed confirm that the DKF motif in PGK1 is required for interaction with α subunit. However, our domain-mapping experiments consistently identified the hinge-containing fragment of α subunit is necessary for robust PGK1 association (Figure 5A and Appendix Figure S5A). We consider this discrepancy may reflect differences between minimal peptide-ear interactions characterized in crystallographic studies and the interaction behavior of full-length PGK1 within the context of the intact AP2 complex. In cells, the hinge and ear regions are structurally and functionally coupled, and the hinge contributes to spatial positioning and conformational flexibility of the ear domain (*Collins et al, 2002*). It is therefore possible that PGK1 engages a composite interface requiring structural elements spanning the hinge-ear region, rather than binding exclusively to the canonical ear pocket characterized for DKF motifs. In addition, we also performed structural prediction of the PGK1-α complex (hinge-appendage domain) using AlphaFold. The model suggests that PGK1 associates with the peripheral hinge-appendage region of the α subunit, consistent with our biochemical mapping results. In this configuration, the DKF motif of PGK1 positioned near the hinge-appendage region, potentially enabling interaction with α subunit (Response Figure 2, shown below). In addition, the flexible hinge region may

provide additional stability for the ear domain to better accommodate PGK1. This further supports reduced binding of PGK1 and hinge-deletion of α subunit (Figure 5A-5B). The appendage domain only, without the hinge region, may lose the flexibility or orientation required for optimal PGK1 docking. However, we realized important limitations of this approach, as the overall confidence scores in AlphaFold predictions for the complex were relatively low (pTM=0.572, iPTM=0.16). The model should be considered a hypothetic model for potential interactions that will require further experimental structural analysis. We have clarified in the revised manuscript that the DKF motif is considered to contribute to the PGK1-AP2 interaction. In addition, we have expanded the Discussion to speculate the structural consequences of hinge region deletion of the α subunit and how this may influence accessibility or positioning of the ear domain.

Ref

Collins BM, McCoy AJ, Kent HM, Evans PR, Owen DJ (2002) Molecular architecture and functional model of the endocytic AP2 complex. Cell 109: 523-535

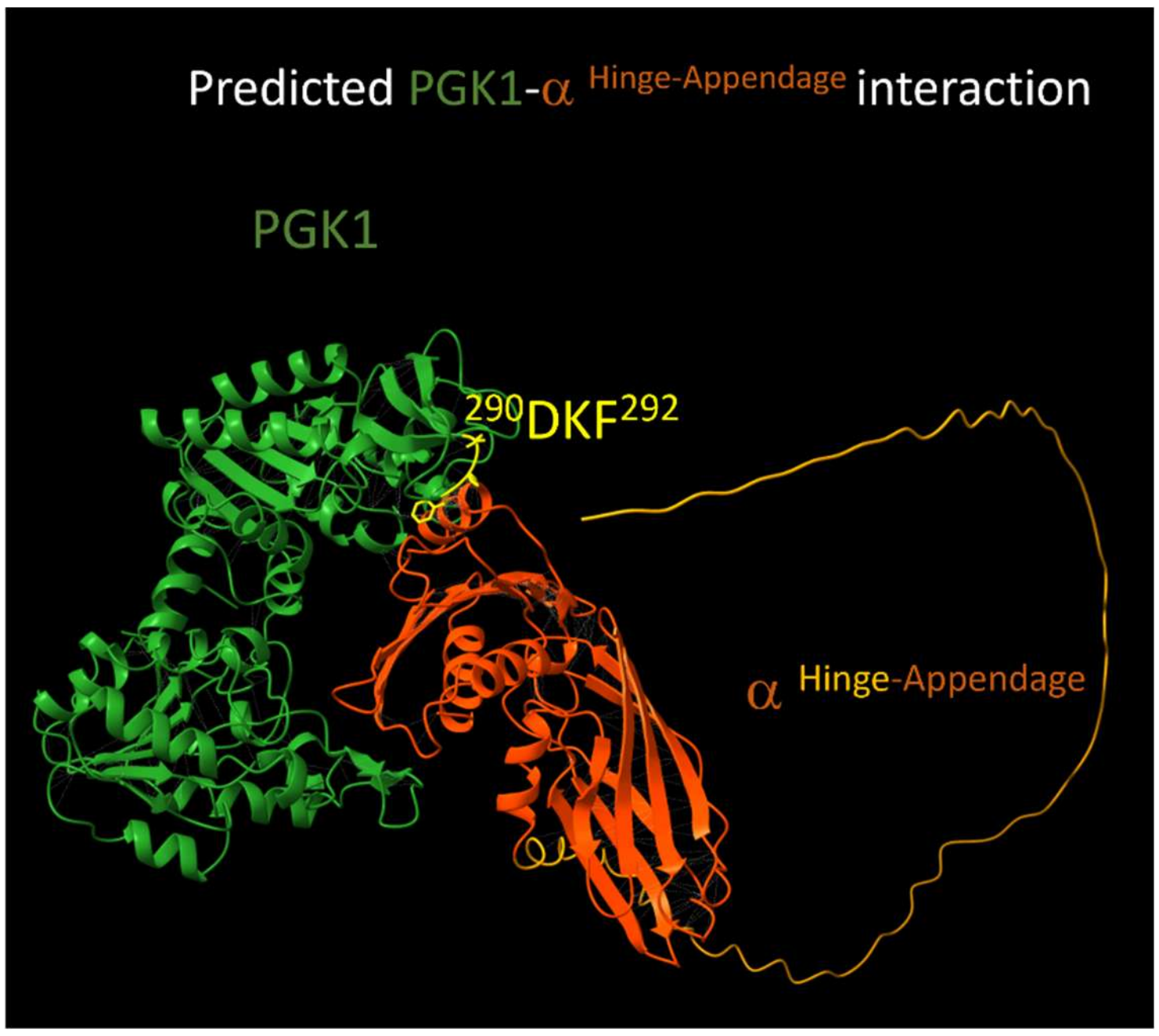

Response Figure 2. Structural prediction of PGK1-α complex

7.) The authors do not discuss any structural data, but show a superficial cartoon. I looked myself, whether the ‚DKF' sequence in PGK1 is at a position, which enables binding to other proteins. It is indeed in a surface loop. Now the binding ‚hinge' motif has to be identified. This positioning of PGK1 to the ‚hinge' domain should indicate, which PGK1 domains are able to bind to μ2 and σ2 domains.

We thank the reviewer for this insightful comment. We agree that our initial schematic representation was too simplified and did not sufficiently discuss structural information. As noted, the DKF motif in PGK1 is positioned within a surface-exposed loop, which is consistent with its potential accessibility for protein-protein interactions. We have now incorporated structural analysis into the revised manuscript to better fit this observation. Regarding the PGK1-α interaction, our mapping experiments indicate that the hinge-containing fragment is required for robust association. Although PGK1 was interacted with μ2 and σ2 subunits *in vitro*, subsequent validation experiments consistently identified α subunit as the dominant interaction partner,

suggesting that PGK1 engages the assembled AP2 complex through α subunit as the bridge scaffold (Figure 2E and 2F). However, from our structural modeling of the AP2-PGK1 complex using AlphaFold, the predictions were generated in the absence of a membrane environment and the conformational transitions associated with AP2 activation (Response Figure 1, shown above). Because AP2 undergoes pronounced structural rearrangements upon membrane recruitment and cargo binding (Kovtun *et al*, 2020), models derived under soluble conditions are unlikely to fully capture the physiologically relevant architecture of the complex. Thus, the predicted structure should be considered a hypothetical model and will require experimental validation, ideally under membrane-mimetic or reconstituted conditions, to establish its structural and functional relevance for PGK1 domains that are able to bind to specific subunit of AP2 complex under physiological conditions. Given this, we have revised the Discussion section to include structural context.

Ref

Kovtun O, Dickson VK, Kelly BT, Owen DJ, Briggs JAG (2020) Architecture of the AP2/clathrin coat on the membranes of clathrin-coated vesicles. Sci Adv 6: eaba8381

These points have to be addressed before the manuscript is suitable for publication in EMBO Reports. However, this manuscript can be much improved by additional data and discussions of the AP2 literature.

We thank the reviewer for this thoughtful evaluation and for recognizing the potential of our study. Specifically, we have incorporated additional experimental data to further validate the specificity and functional relevance of the PGK1-AP2 interaction. Furthermore, we have substantially expanded the Discussion to provide a more comprehensive integration of the AP2 literature, including structural studies of α subunit, canonical interaction with DKF motifs, and the conformational regulation of AP2 complex during membrane recruitment and cargo engagement. We believe these additions significantly strengthen the mechanistic foundation of our study and more clearly position our findings within the established modeling of AP2 biology. We really appreciate the reviewer’s suggestions, which have greatly improved the clarity and impact of the manuscript.

Referee 2

The authors demonstrate that the interaction between PGK1 and AP-2 is essential for the efficient endocytosis of specific cargo proteins, such as TfnR and EGFR. They further map the interaction interfaces between PGK1 and AP-2 that are required for their roles in the endocytic pathway. Together, these findings reveal a previously unrecognized regulatory layer in clathrin-mediated endocytosis. However, several concerns remain and should be addressed.

1. The data presented in this study support a role for the PGK1-AP-2 interaction in clathrin-mediated endocytosis; however, the authors do not provide evidence for downstream cargo-sorting events following internalization, despite PGK1's ability to recognize TfnR and EGFR sorting signals. The authors should therefore temper their statements regarding cargo sorting throughout the manuscript.

We thank the reviewer for this important comment. We agree that our data primarily demonstrated a role for the PGK1-AP2 interaction in clathrin-mediated endocytosis at the level of cargo recognition and internalization. In response to this concern, we have revised the manuscript to more precisely describe the

functional scope of our findings. Specifically, we have tempered statements referring broadly to cargo sorting and now clarify that our data support a role for PGK1 in cargo recognition and efficient internalization during clathrin-mediated endocytosis. We appreciate this suggestion, as it improves the accuracy and clarity of our conclusions while maintaining the mechanistic significance of the PGK1-AP2 interaction in early endocytic events.

2. The quality of immunofluorescent data in the first half of the manuscript were not sufficient to support their statement (Fig. 4A; 7C; EV4A; EV5D, E; EV7A, B). The cytosol washout step, together with the use of super-resolution confocal microscopy and TIRF imaging, substantially improved the quality of the immunofluorescence data. The authors may consider presenting only these higher-quality datasets and integrating them into the earlier sections of the manuscript.

We thank the reviewer for this helpful suggestion. In the revised manuscript, we have prioritized presentation of these higher-quality datasets and integrated them into the main figures where appropriate. Specifically, we have replaced the earlier immunofluorescence panels with the improved imaging data to support the key mechanistic conclusions. These may include Figure 4A, 7C, 7D, Appendix Figure S5E (original EV5D), Appendix Figure S5F (original EV5E), Appendix Figure S7A and S7B (original Figure EV7A and EV7B). We believe these revisions significantly enhance the overall clarity of the manuscript. We appreciate the reviewer's recommendation, which has greatly improved the presentation of our findings.

3. The authors should provide the raw MS analysis data, including the associated statistical analyses, to substantiate their rationale for focusing on AP-2α.

We appreciate the reviewer's comment regarding the raw MS data and statistical analysis. In this study, the label free proteomic analysis was conducted as a single run exploratory experiment; therefore, conventional statistical analyses (such as replicate based quantitative comparisons) were not applicable. Instead, we assessed potential PGK1-interacting proteins using the Normalized PSM (%), a commonly used semi quantitative measure of relative protein abundance in label free proteomics. To prioritize candidates for downstream validation, we selected the top 100 proteins ranked by Normalized PSM (%) (independent of EGF treatment). From this high confidence set, we focused on molecules involved in vesicular trafficking, among which α subunit of AP2 complex emerged as a notable candidate. Specifically, we further performed immunoprecipitation experiments and observed the association with the all four subunits, α, μ2, σ2 and β2, of AP2 complex under identical experimental conditions (Figure 2A). Consistently, our finding is further supported by a recent report showing that a PGK1 TurboID dataset identified AP2A2 as one of the major hits (Camara *et al*, 2025). The PGK1-interacting proteins identified by label free proteomic analysis in this study have been provided in the revised Table EV1.

Ref

Camara GA, Yokoo S, Granato DC, Simabuco FM, Ribeiro-Filho HV, Melo RM, Pauletti BA, Nascimento Filho EG, Domingues RR, Paes Leme AF (2025) Mapping the Interactome of OSCC Prognostic-Associated Proteins NDRG1 and PGK1 Through Proximity Labeling Using TurboID. J Proteome Res 24: 2741-2756

4. For the CHX-treatment protein degradation assay, the analysis should be normalized to the 0-h CHX time point for each condition to accurately assess degradation rates.

We thank the reviewer for this important suggestion. To carefully and accurately assess the degradation pattern, we have revisited the CHX chase assay by normalizing protein levels to their respective 0-h time points for each condition and calculating the protein half-life ($t_{1/2}$) accordingly. The quantification is now presented in the revised figures (Figure 3C, Appendix Figure S5D, S6B, and S6C). This normalization does not alter the overall conclusion but further provides a more rigorous and accurate comparison of degradation kinetics across conditions.

5. It has been reported that the interaction between AP-2 and PIP_2 induces the open conformation of AP-2, which is also required for its interaction with clathrin (PMID: 25061211). It is therefore surprising that AP-2α-dH still reduces the interaction between AP-2 and clathrin, despite retaining the appendage domain (Fig. 7F). Moreover, the data in Fig. 4F indicate that PIP_2 binding enhances the affinity between AP-2 and PGK1, suggesting that the AP-2-PIP_2 interaction may also be necessary for PGK1-AP-2 association. These observations are novel and potentially significant for the field, and the authors may need to discuss these points in greater depth.

We thank the reviewer for raising these important mechanistic points. We speculate that deletion of the hinge region may alter the spatial orientation or conformational flexibility of the appendage domain, thereby impairing efficient clathrin recruitment even if the canonical clathrin-binding motifs remain present. The hinge is known to contribute to structural coupling between the core and appendage domains, and its removal may compromise the ability of AP2 complex to fully adopt or stabilize the open conformation. It is possible that the flexible hinge region may provide additional stability for the appendage domain to better accommodate PGK1. This further supports reduced binding of PGK1 and hinge-deletion of α subunit (Figure 5A-5B). The appendage domain only, without the hinge region, may lose the flexibility or orientation required for optimal PGK1 docking. We have expanded the Discussion to address this structural consideration more explicitly. Regarding the role of $PI(4,5)P_2$ in PGK1 association, our liposome-containing assay indeed suggested that $PI(4,5)P_2$ enhances AP2-PGK1 interaction. We agree that this finding may indicate that membrane-associated, activated AP-2 provides a favorable platform for PGK1 recruitment. It is possible that PGK1 preferentially interacts with the open conformation of AP2 stabilized by $PI(4,5)P_2$ and cargo engagement. We have incorporated a more detailed discussion of how membrane lipids, conformational activation, and adaptor assembly may cooperatively regulate PGK1 recruitment. We appreciate the reviewer's insightful comments, as these points highlight potentially significant mechanistic implications of our findings and enhance the conceptual framework of AP-2 regulation during endocytosis.

Minor comments:

1. The WB blot data were missing to demonstrate the protein knockdown and reconstitution efficiency.

We thank the reviewer for pointing out this omission. In the revised manuscript, we have included representative western blot data showing the efficiency of protein depletion and the expression levels of

reconstituted constructs under the experimental conditions used (Appendix Figure S1F, S5B, and S6A).

2. Quantification data were missing for some WB blots and IF imaging to support the statements (Fig. 2F, G; EV2C; EV4D; EV5C).

We thank the reviewer for the critical comments. In the revised manuscript, we have included quantification for the indicated figures (Figure 2E (original 2F), 2F (original 2G), Appendix Figure S2E (original Figure EV2C), Appendix Figure S4D (original Figure EV4D), and Appendix Figure S5D (original Figure EV5C)). These additions strengthen the reproducibility of the data to support our conclusions.

Referee 3

In this study, Chu et al aimed to elucidate a novel function of PGK1 in regulating clathrin-mediated endocytosis (CME) beyond its well-established roles in glycolysis. Biochemical assays and fluorescent microscopy imaging have been utilized to show that PGK1 interacts with and stabilizes AP2 complex, which is correlated with its cargo binding capacity but independent of its kinase activity. Overall, the novel function of PGK1 in CME regulation is intriguing to cell biologists; however, several issues remain in the mechanistic elucidation that require further validation.

Major points:

1. The authors claim that PGK1 mainly binds to the α subunit of the AP-2 complex, which functions as a bridge linking PGK1 with other subunits of the AP2 complex. To comprehensively validate this conclusion, the below concerns about the biochemical assays of PGK1-AP2 interactions should be addressed:

a) All subunits of the endogenous AP-2 complex in Co-IP experiments should be examined by WB. For example, AP2-β and AP2-σ should be examined in Fig. 2B and Fig. EV2C.

We thank the reviewer for this valuable suggestion. Accordingly, we have performed additional co-immunoprecipitation experiments and examined the presence of β2 and σ2 subunits by western blot in the same conditions (Figure 2A (original Figure 2A and 2B) and Appendix Figure S2E (original Figure EV2C)). The results showed that, consistent with the behavior of α, both β2 and σ2 co-immunoprecipitate with PGK1, confirming that the entire AP2 complex is associated with PGK1. We believe these additional experiments fully address the reviewer’s concern and further support our conclusions regarding PGK1-AP2 complex interactions.

b) AP2-σ should be examined in Fig. 3A,B.

We thank the reviewer for the suggestion. To comprehensively address the concern regarding AP2 subunits, we have now included σ2 to monitor the stability of the entire AP2 complex (Figure 3A and 3B). These additional data show that all major AP2 subunits are less stable in siPGK1-treated cells, confirming that PGK1 is required to maintain the stability of the AP2 complex.

c) Based on the results shown in Fig. 2F,G, it looks that PGK1 binds stronger to AP2 complex. However, it is difficult to directly conclude that AP2-α act as a bridge linking PGK1 and AP2 complex. It would be more

convincing if the authors can show that: i) siRNA knockdown of AP2-β or AP2-μ does not affect PGK1 interaction with AP2-α; ii) replace AP2-α with AP2-β in the GST-pulldown assay shown in Fig. 2F,G does not enhance PGK1 binding to AP2-μ and AP2-σ.

We thank the reviewer for the insightful comment. We agree that directly demonstrating the bridging role of α subunit of AP2 complex would strengthen our conclusions. Based on the reviewer's suggestion, we have addressed this concern through two key points:

i) siRNA knockdown of AP2-μ: We performed depletion of μ2 (siAP2M-treated cells) and subsequently examined the interaction between PGK1 and the α subunit. Consistent with the heterotetrameric organization of the AP2 complex, loss of μ2 led to a marked reduction in α protein levels, likely due to destabilization of the entire AP2 complex. The decreased abundance of α subunit under this condition prevented a reliable assessment of its association with PGK1 (Appendix Figure S2F). This result therefore did not allow us to definitively evaluate whether AP2-μ depletion directly affects the PGK1 and α subunit interaction.

ii) GST pulldown with AP2-β: We also performed a GST pulldown assay replacing recombinant α subunit with recombinant β2 subunit under the same conditions (Appendix Figure S2C and S2D). In the presence of the β2 subunit, PGK1 did not exhibit enhanced association with the μ2 and σ2 subunits, in contrast to the effect observed with the α subunit., further supporting the specific bridging role of α subunit in linking PGK1 to the AP2 complex.

These additional experiments strengthen our conclusion that α subunit serves as the primary bridge mediating the association between PGK1 and the AP2 complex. The new data have been incorporated into the revised manuscript.

d) If PGK1 can bind to AP2 complex, why AP2-μ and AP2-σ was not detected in the MassSpec result of PGK1-interacting proteins that are listed in Table EV1?

We thank the reviewer for this concern. We would like to clarify that μ and σ subunits were indeed detected in the mass spectrometry analysis. Although their peptide counts and sequence coverage were lower compared to the α subunits, their identification supported the association of PGK1 with the intact AP2 complex rather than with individual subunits. The PGK1-interacting proteins identified by our proteomic analysis in this study have been provided in the revised Table EV1. Consistently, our finding is further supported by a recent report showing that a PGK1 TurboID dataset identified α subunit as one of the major hits (Camara *et al*, 2025). In addition, we also confirmed the association of PGK1 and entire AP2 complex, α, μ2, σ2 and β2, in cells using immunoprecipitation (Figure 2A). We have revised the manuscript to more clearly present this data and to avoid any ambiguity regarding the detection of AP2 subunits in the mass spectrometry dataset.

<u>Ref</u>

Camara GA, Yokoo S, Granato DC, Simabuco FM, Ribeiro-Filho HV, Melo RM, Pauletti BA, Nascimento Filho EG, Domingues RR, Paes Leme AF (2025) Mapping the Interactome of OSCC Prognostic-Associated Proteins NDRG1 and PGK1 Through Proximity Labeling Using TurboID. J Proteome Res 24: 2741-2756

e) FxDxF or DxF motifs are classical AP2 α-AD interacting motifs, how to explain that PGK1 barely binds to α-AD domain? Although the role of DKF in mediating the interaction between PGK1 and AP2 is well validated,

the authors could employ AlphaFold prediction to gain deeper insights into their interactions.

We thank the reviewer for this important mechanistic concern. FxDxF and DxF motifs are indeed well-established ligands for the α appendage domain (α-AD). Although PGK1 contains a DKF motif and mutation of this sequence disrupts the PGK1-AP2 interaction, our binding assays indicated that PGK1 does not robustly associate with the isolated α-appendage domain. In the context of the intact AP2 complex, the hinge and appendage domains are structurally coupled, and productive engagement may depend on conformational states associated with AP2 activation at membranes. Thus, PGK1 may recognize a composite or conformationally regulated interface that is not fully recapitulated by the isolated α-AD fragment. To gain further mechanistic insight, we performed AlphaFold structural modeling of the AP-2-PGK1 complex (Response Figure 3, shown below). The DKF motif on PGK1 was shown to be close to α-AD domain. The hinge region is known to contribute to structural coupling between the core and appendage domains, and its removal may compromise the ability of AP2 complex to fully adopt or stabilize the open conformation. It is possible that the flexible hinge region may provide additional stability for α-AD domain to better accommodate PGK1. This further supports reduced binding of PGK1 and hinge-deletion of α subunit (Figure 5A-5B). The appendage domain only, without the hinge region, may lose the flexibility or orientation required for optimal PGK1 docking. However, we recognized several important limitations of this approach, as the overall confidence scores in AlphaFold predictions for the complex were relatively low (pTM=0.572, iPTM=0.16). The structural prediction was performed without incorporating a membrane environment, lipid components such as PI(4,5)P_2, or the conformational transitions that accompany AP2 activation. Given that AP2 undergoes significant structural rearrangement upon membrane recruitment and cargo binding, models generated under soluble conditions may not fully represent the physiologically relevant architecture or stability of the complex. Accordingly, the predicted structure should be considered as a provisional model highlighting potential contact interfaces and will require experimental validation to establish its structural and functional relevance. We have expanded the Discussion to address this structural consideration more specifically.

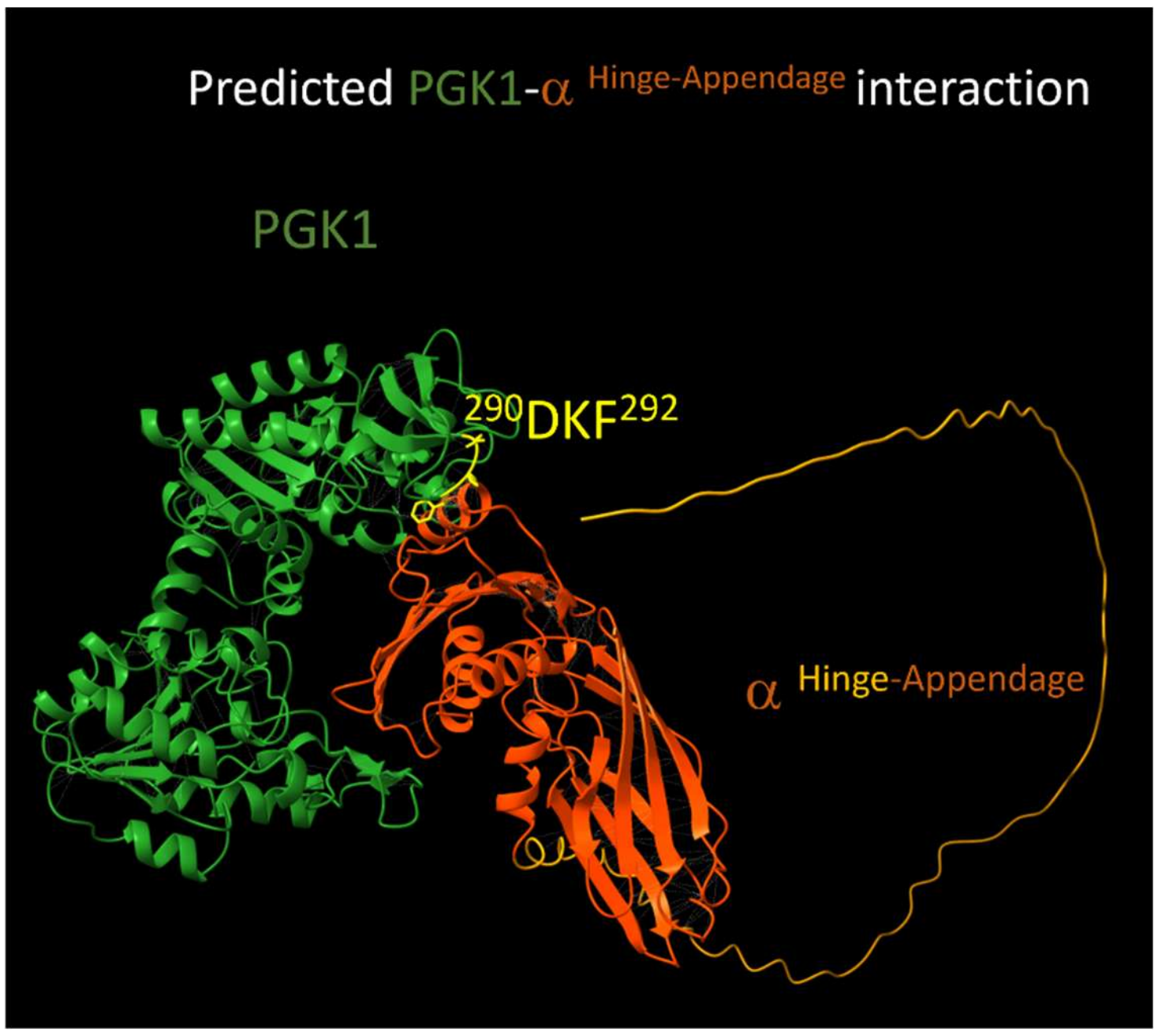

Response Figure 3. Structural prediction of PGK1-α complex

2. The effects shown in figure 3C-F are too weak to support the role of PGK1 in regulating the stability of AP-2 complex. Stronger experimental data are needed, please repeat that, and detect the ubiquitination levels of AP2-α under PGK1 knockdown.

We thank the reviewer for this valuable suggestion. In response, we have repeated these experiments with increased biological replicates and have improved signal and quantification (Figure 3C-3F). The revised data are now presented in the manuscript to enhance the conclusion that PGK1 contributes to AP2 complex stability. Furthermore, as suggested, we have examined the ubiquitination status of α subunit in PGK1 knockdown cells. Our results showed that depletion of PGK1 leads to increased ubiquitination of α subunit (Appendix Figure S3B), supporting a role for PGK1 in protecting AP2 complex from destabilization for proteasomal degradation. These new data have been incorporated into the revised manuscript and further substantiate the functional significance of the PGK1-AP2 interaction.

3. The Confocal Microscopy results in quantifying the colocalizations of PGK1, AP2-α and cargos are not convincing. Based on the representative images shown in Figs. 4, EV4 and EV5, no obvious colocalizations were observed between PGK1 and EGF or Tf; however, the authors' quantification analysis showed high overlap coefficients. The discrepancy raises concerns about the accuracy of the method. In addition, it is not clear how the authors chose where and what direction to draw the lines as shown in Fig. 4A and Fig. EV5D,E. Thus, whether these fluorescent curves are representative is in question.

We thank the reviewer for carefully examining the images and for raising these important concerns. We acknowledge that, in some representative confocal images, the degree of colocalization between PGK1 and EGF or transferrin is not visually striking. This is partly due to the high cytosolic background of PGK1 and the diffraction-limited resolution of conventional confocal microscopy, which can obscure discrete membrane-associated puncta. To address this concern, we have taken several steps in the revised manuscript. First, we have replaced selected confocal images with higher-resolution datasets obtained using super-resolution confocal microscopy imaging, which significantly improve visualization of membranous events. Second, we have performed the quantitative colocalization analysis by specifying the method used, e.g., thresholded Manders' split coefficient in the Methods section. We wish to point out that quantification was performed using automated image analysis pipelines to minimize observer bias, and the statistical analysis has been carefully evaluated accordingly. Regarding the line-scan analyses, we agree that the criteria for line placement were not sufficiently described. In the revised manuscript, we have clarified that line scans were drawn across regions containing clearly puncta in both channels including regions of interest containing cargo-positive structures. The direction and position of line scans were not arbitrarily chosen but were applied consistently across multiple cells. The signals across the line scans were used to enhance the colocalization signals quantitatively among different organelle markers, adaptors, or cargoes, which more accurately reflect spatial coincidence at the molecular level. We have now provided representative images in the revised manuscript. Together, these revisions have been improved the quality and reproducibility of the imaging analysis and better reconciled the quantitative observations. We appreciate the reviewer's critical comment, as it substantially improves the presentation of the imaging analyses.

Minor point:

1. The authors should explain in Methods why they chose Manders' Overlap Coefficient rather than Pearson's Correlation Coefficient in quantifying the colocalization signals. Moreover, details on how the authors conduct the Manders' Overlap Coefficient analysis should be included. How do the authors make sure that the background subtraction and threshold setting won't affect the result interpretation.

We thank the reviewer for raising this important point regarding the colocalization analysis. The colocalization was quantified using the thresholded Manders' split coefficient (tM1 and tM2), which measures the fraction of signal in one channel that overlaps with signal in the other channel. To minimize the influence of background, intensity thresholds were applied prior to coefficient calculation. Background subtraction was performed using ImageJ program, and identical analysis parameters were applied across all images within the same experimental dataset. The tM1 and tM2 therefore reflect the overlap of signals above background levels, reducing potential bias. PCC is sensitive to proportional intensity variation and can be influenced by differences in signal distribution and background levels, which may underestimate colocalization in cases where signals are spatially coincident but vary in intensity. In contrast, tM1 and tM2 quantifies the proportion of one signal that overlaps with the second signal, making them more convincible for analyzing punctate endocytic structures. To ensure the reproducibility, we have now expanded the Methods section to provide detailed information regarding image preprocessing and quantification. Additionally, to further enhance the consistency regarding the imaging colocalization analysis, we further confirmed that colocalization test using PCC also demonstrated very consistent results compared to that using tM1 and tM2 (Figure 1B and Appendix Figure S1E), indicating our conclusions remain consistent to avoid the bias caused by systemic background subtraction and threshold setting. These details have now been incorporated into the revised Methods and Figure Legends.

1st Revision - Editorial Decision **18th May 2026**

Dear Prof. Hsu

Thank you for the submission of your revised manuscript to EMBO reports. I apologize for the delay in notifying you of the manuscript's progress. We have now received the full set of referee reports that is copied below along with discussion between the referees on the revised manuscript.

As you can see, the referees find that the study is significantly improved during revision but request changes to the Discussion or figure presentation and one additional experiment.

From the editorial side, there are also a few things that we need:

- As a standard procedure, we edit the title and abstract of manuscripts to make them more accessible to a general readership. Please find the suggested versions below my signature.

- Please ensure that the manuscript sections are in the following order: Title page - Abstract & Keywords - Introduction - Results - Discussion - Methods - Data Availability - Acknowledgments - Disclosure Statement & Competing Interests - References - Figure Legends - (Main Tables with legends if applicable) - (Expanded View Figure Legends if applicable).

- CRediT has replaced the traditional author contributions section because it offers a systematic machine readable author contributions format that allows for more effective research assessment. Please remove the Author Contributions section from the manuscript and use the free text boxes beneath each contributing author's name in our online systems to add specific details on the author's contribution. More information is available in our guide to authors.

- Please rename Table EV1 to Dataset EV1 in all places - source file name, legend in the file, title in the system, and callouts in the mansucript. The file type in the system should be Data set.

- Please include a statement on blinding in measurements even if no blinding was performed.

- Please remove the instructions from the Reagents and Tools Table.

-Please upload the Appendix file in pdf format with the author list removed.

- Please note that EMBO press papers are accompanied online by:
A) a short (2 sentences) summary of the findings and their significance,
B) 2-5 short bullet points highlighting the key results, and
C) a synopsis image in .jpg or .png format that is exactly 550 pixels wide and 300 pixels high. Please note that the text needs to be legible at the final size. Please upload this information along with your revised manuscript (the text for A and B should be provided in one separate Word file uploaded as Cover Art/Synopsis Image).

- Since the Appendix file has its figures combined in a single file, please upload the source data folders for the Appendix figures in one zip folder. Please rename the panels Appendix Fig. S1, etc.

- Our Figure check identified possible reuse of cells between Appendix Fig. S1H and Appendix Fig. S6H. Can you explain how this may have come about?

- Can you also clarify the white boxes in the merged cells for figures 4A and 7C,D,E,F?

- Our production/data editors have asked you to clarify several points in the figure legends (see below). Please incorporate these changes in the manuscript and return the revised file with tracked changes with your final manuscript submission.
- Please note that the scale bar is missing for figures 8B, D, F
- Please note that the white boxes are not defined in the legend of figures 7C-F. This needs to be rectified.

I look forward to receiving the revised version of your manuscript.

Yours sincerely,

Kurt Weir
Editor
EMBO Reports

Phosphoglycerate kinase 1 cooperates with the AP2 complex to promote endocytosis

Adaptor proteins play a critical role in intracellular protein transport by binding to cargoes and facilitating their sorting into vesicles. The adaptor protein 2 (AP2) complex specifically recognizes sorting motifs on cargo proteins such as epidermal growth factor receptor (EGFR) and transferrin receptor (TfR) for proper cargo recognition. However, the coordination of adaptor proteins in cargo recognition remains unclear. Here, we uncover the non-canonical role of glycolytic enzyme phosphoglycerate kinase 1 (PGK1) in protein endocytosis, acting independently of its catalytic activity. PGK1 serves as a co-adaptor to directly engage cargoes and reinforce AP2 complex stability, orchestrating efficient cargo recognition to promote endocytosis. Furthermore, PGK1 also integrates into the AP2-clathrin complex, enhancing cargo recognition and internalization. Collectively, our findings establish a previously unidentified coat protein complex containing PGK1, highlighting its dual role as a cargo adaptor and stabilizer of the AP2 complex.

Referee #1:

The revised manuscript addresses the AP-cpmplex specificity of PGK1. However, the revised version does not resolve the problem that a PGK1 sequence motif expected to interact with the alpha-adaptin ear domain binfs instead to the hinge domain. There are no new data and this finding is not discussed at all. The discussion needs to be improved in general. For example on page 6, the result that all 4 subunits were detected in an IP is being interpreted that PGK1 binds to all 4 subunits. However, monomeric AP2 adaptins are not found in cell extracts and the AP2 complex is in fact very stable and requires the use of detergents to separate the subunits. High salt concentrations or urea don't separate the subunits. The alpha-adaptin hinge region is proline-rich and highly flexible allowing the ear-domain to „catch" its binding partners. The PGK1 - hinge-domain interaction should be discussed with this property in mind.

Referee #2:

The authors addressed the comments sufficiently.

Referee #3:

The authors have made substantial modifications on the manuscript, which shows valid improvement on the presentation of the story.

Regarding my major concern about the interactions between PGK1 and AP2 complex, new data have been added to show the interactions between PGK1 and all the four subunits of AP2, and to demonstrate that AP2 α subunit plays a central role in mediating these interactions.

However, issues remain in the data quality and the interpretation of results shown in Figure 3C-F (CHX treatment combined with MG132 treatment) and Figure S3B (HA_Ub pulldown assay). First, the impression of the effects shown in Figures 3C and 3E are still too weak and not consistent with the quantifications shown in Figures 3D and 3F. In Figure 3C, the Tubulin levels in the representative gel are quite different across samples. In Figure 3E, MG132 rescue effect was not obvious. Second, in appendix figure S3B, HA-Ub pulldown assay was not convincing to demonstrate that the lower levels of the AP2 complex in PGK1 knockdown cells are due to proteasomal degradation: polyubiquitinated AP2 α is supposed to be digested by proteasomes; however, the presented WB result only indicate the non-ubiquitinated AP2 α, but not polyubiquitinated AP2 α. A proper experimental design would be to pulldown AP2 α and then use ubiquitin antibody to detect the ubiquitination of AP2.

Minor point:
The microscopy images have been remarkedly improved, and the corresponding statistical methods have been well explained. The results now clearly show that PGK1 colocalized with AP2 and protein cargoes. Nonetheless, solid squares were mistakenly added into most of the merge image, which should be changed to open squares (i.e: in figure 4A and figure 7C-F).

Selected Comments from cross-comment:

Referee 1: I do agree with the concerns raised by reviewer 3. I did not make such detailed comments myself, because I thought it is sufficient to just list those two examples to underline my major criticisms.

Referee 2: I agree with the comments raised by reviewer 3.

Referee 3: I highly appreciate the novelty of the story, but agree with the criticisms regarding the interpretation of the data. Major revisions are required for the manuscript textually.

Responses to the editorial board (EMBOR-2025-63143V3)

-As a standard procedure, we edit the title and abstract of manuscripts to make them more accessible to a general readership. Please find the suggested versions below my signature.

We thank the editor for the careful revision of the title and abstract to improve readability for a broader audience. We have confirmed the revised version.

- Please ensure that the manuscript sections are in the following order: Title page - Abstract & Keywords - Introduction - Results -Discussion - Methods - Data Availability - Acknowledgments - Disclosure Statement & Competing Interests - References - Figure Legends -(Main Tables with legends if applicable) - (Expanded View Figure Legends if applicable).

We have confirmed the order.

- CRediT has replaced the traditional author contributions section because it offers a systematic machine readable author contributions format that allows for more effective research assessment. Please remove the Author Contributions section from the manuscript and use the free text boxes beneath each contributing author's name in our online systems to add specific details on the author's contribution. More information is available in our guide to authors.

We have removed the Author Contributions section and specified the contribution in the online systems.

- Please rename Table EV1 to Dataset EV1 in all places - source file name, legend in the file, title in the system, and callouts in the mansucript. The file type in the system should be Data set.

We have renamed and corrected in the manuscript.

- Please include a statement on blinding in measurements even if no blinding was performed.

We have added the statement on blinding in the manuscript.

- Please remove the instructions from the Reagents and Tools Table.

We have removed the instructions as requested.

-Please upload the Appendix file in pdf format with the author list removed.

We have changed the format and removed the author list as requested.

-Please note that EMBO press papers are accompanied online by:

A) a short (2 sentences) summary of the findings and their significance,

B) 2-5 short bullet points highlighting the key results, and

C) a synopsis image in .jpg or .png format that is exactly 550 pixels wide and 300 pixels high. Please note that the text needs to be legibleat the final size. Please upload this information along with your revised manuscript (the text for A and B should be provided in one separateWord file uploaded as Cover Art/Synopsis Image).

We have uploaded the summary, bullet points, and synopsis as requested.

- Since the Appendix file has its figures combined in a single file, please upload the source data folders for the Appendix figures in one zipfolder. Please rename the panels Appendix Fig. S1, etc.

We have uploaded single source data folder for the Appendix figures as requested.

- Our Figure check identified possible reuse of cells between Appendix Fig. S1H and Appendix Fig. S6H. Can you explain how this may havecome about?

We apologize for this mistake. During our figure preparation, the image in Appendix Fig. S1H was accidentally replaced with the image used in Appendix Fig. S6H, resulting in the duplication. This mistake was not noticed prior to submission. However, the original source data uploaded with the manuscript were actually derived from two independent datasets, confirming that the underlying experiments were distinct. We have now corrected the figure and replaced the incorrect panel with the appropriate images.

- Can you also clarify the white boxes in the merged cells for figures 4A and 7C,D,E,F?

We thank the editor for pointing out the lack of clarity. To avoid confusion, we have now revised the corresponding figure legends and removed the white boxes presented in each figure.

- Our production/data editors have asked you to clarify several points in the figure legends (see below). Please incorporate these changes in the manuscript and return the revised file with tracked changes with your final manuscript submission.

We have revised the file with tracked changes in the manuscript.

- Please note that the scale bar is missing for figures 8B, D, F

We have added or enhanced the scale bar for figures 8B, D, F.

- Please note that the white boxes are not defined in the legend of figures 7C-F. This needs to be rectified.

We have removed the white boxes in the figure to avoid confusion.

Responses to the reviewer (EMBOR-2025-63143V3)

In response to our revision, the reviewers have provided further comments. They are listed below along with our point-by-point responses, which include additional experiments that we have performed. Some responses also involve revising the manuscript text. These changes are revised with tracked change in our manuscript.

Referee #1:The revised manuscript addresses the AP-cpmplex specificity of PGK1. However, the revised version does not resolve the problem that aPGK1 sequence motif expected to interact with the alpha-adaptin ear domain binfs instead to the hinge domain. There are no new data and this finding is not discussed at all. The discussion needs to be improved in general. For example on page 6, the result that all 4 subunits were detected in an IP is being interpreted that PGK1 binds to all 4 subunits. However, monomeric AP2 adaptins are not found in cell extracts and the AP2 complex is in fact very stable and requires the use of detergents to separate the subunits. High salt concentrations or urea don't separate the subunits. The alpha-adaptin hinge region is proline-rich and highly flexible allowing the ear-domain to „catch" its binding partners. The PGK1 - hinge-domain interaction should be discussed with this property in mind.

We thank the reviewer for this insightful comment. Because the AP2 complex exists as a highly stable heterotetramer in cells, the detection of α, β2, μ2, and σ2 subunits from PGK1 immunoprecipitation does not necessarily indicate that PGK1 independently binds to each subunit. Instead, these observations are consistent with PGK1 association with the assembled AP2 complex. We have revised the Results section to clarify this interpretation and avoid implying direct interaction with all four adaptins. We also appreciate the reviewer's comments regarding the PGK1 and α interaction. We agree that this issue merits further discussion. Previous structural studies have established that DxF motifs typically engage the α-adaptin ear domain, whereas the hinge region serves as a highly flexible linker that spatially positions the ear domain and facilitates interactions with endocytic accessory proteins. In light of these findings, we do not interpret our data as evidence that the PGK1 DKF motif directly binds a discrete site within the hinge region. Instead, we propose that deletion of the hinge-containing region may disrupt the conformational flexibility, accessibility, or spatial presentation of the adjacent ear domain, thereby impairing productive interaction with PGK1. Thus, the requirement for the hinge-containing fragment identified in our mapping experiments may reflect a structural contribution of the hinge-ear module rather than a direct docking interface within the hinge itself.

To address this point, we have substantially expanded the Discussion section to incorporate current knowledge of AP2 architecture, the structural role of the α-adaptin hinge region, and the limitations of our current mapping data. We now conclude that the precise molecular interface between PGK1 and AP2 remains unresolved and will require future structural and mutational analysis.

Referee #2:The authors addressed the comments sufficiently.

Referee #3:The authors have made substantial modifications on the manuscript, which shows valid improvement on the presentation of the story. Regarding my major concern about the interactions between PGK1 and AP2 complex, new data have been added to show the interactions between PGK1 and all the four

subunits of AP2, and to demonstrate that AP2 α subunit plays a central role in mediating these interactions. However, issues remain in the data quality and the interpretation of results shown in Figure 3C-F (CHX treatment combined with MG132treatment) and Figure S3B (HA_Ub pulldown assay). First, the impression of the effects shown in Figures 3C and 3E are still too weak and not consistent with the quantifications shown in Figures 3D and 3F. In Figure 3C, the Tubulin levels in the representative gel are quite different across samples. In Figure 3E, MG132 rescue effect was not obvious.

We thank the reviewer for this careful evaluation of the data presentation. To address this concern, we have re-examined and replaced the original data and revised the presentation of Figures 3C and 3E.

For Figure 3C, we noted that the tubulin loading control displayed variability among the representative lanes, which may affect the visual interpretation of α protein levels. We have therefore replaced the representative blot with an independent experiment that more accurately reflects the loading normalization and the quantitative results.

For Figure 3E, we noted that the rescue effect of MG132 was not readily apparent in the originally presented image. We have therefore replaced the representative blot with a more representative experiment. The revised data consistently demonstrate a significant restoration of α protein levels following MG132 treatment.

We have revised the figures accordingly to ensure that the representative images more obviously reflect the quantification and improve the clarity of data interpretation.

Second, in appendix figure S3B, HA-Ub pulldown assay was not convincing to demonstrate that the lower levels of the AP2 complex in PGK1 knockdown cells are due to proteasomal degradation: polyubiquitinated AP2 α is supposed to be digested by proteasomes; however, the presented WB result only indicate the non-ubiquitinatedAP2 α, but not polyubiquitinated AP2 α. A proper experimental design would be to pulldown AP2 α and then use ubiquitin antibody to detect the ubiquitination of AP2.

We thank the reviewer for this important comment. To address this concern, we have performed the suggested experiment by immunoprecipitating α followed by detecting the ubiquitin levels in each group. Consistent with our hypothesis, α ubiquitination was increased in siPGK1-treated cells compared with control cells, supporting the notion that loss of PGK1 promotes ubiquitination of α. These new data have been included in the revised manuscript (Appendix Figure S3B).

Minor point: The microscopy images have been remarkedly improved, and the corresponding statistical methods have been well explained. The results now clearly show that PGK1 colocalized with AP2 and protein cargoes. Nonetheless, solid squares were mistakenly added into most of the merge image, which should be changed to open squares (i.e: in figure 4A and figure 7C-F).

We thank the reviewer for the positive assessment of the revised microscopy data and statistical analyses. We also appreciate the reviewer for identifying the figure annotation issue. To avoid confusion, we have corrected these annotations in the revised figures and updated the corresponding figure legends accordingly.

Dear Prof. Hsu

Thank you for the submission of your revised manuscript to our offices. We have now received the enclosed reports from the referees that were asked to assess it. EMBOR-2025-63143V3 still has minor suggestions that I would like you to incorporate before we can proceed with the official acceptance of your manuscript.

Additionally, during editorial checks, I failed to find the scale bars for figures 8B, D, F.

We are looking forward to receiving the final version of your manuscript as soon as possible.

With kind regards,

Kurt Weir
Editor
EMBO Reports

Referee #1:

Review of the manuscript: Phosphoglycerate kinase 1 cooperates with AP-2 complex to promote endocytosis Authors: Jia-Wei Hsu, Shao-Ling Chu, Yu-Tzu Chang, Che-Yu Hung, and Chia-Jung Yu Manuscript Number: EMBOR-2025-63143V3

Dear authors,

The discussion of your PGK1 ‚DKF' data is much improved in the current version of the manuscript. You may have pointed out that this motif is not a canonical adaptin ‚ear'-domain ‚DKF' motif, which was the reason why you mutated these 3 residues in PGK1. You could have introduced a nomenclature for the PGK1 ‚DKF' motif, which should be able to prevent a confusion.

I want to point out two problems I have with your presentation of your microscopic data and your semi-quantitative western-blot experiments. Some of these were already mentioned by another reviewer of the second version of your manuscript.

1. You show in your microscopic analysis only single cells. You should show an overview of the slide which shows several cells instead and one of these cells you should enlarge. This is the common way to present such data. You may update your supplements accordingly.

2. You have used tubulin as a control protein in your semi-quantitative western-blot experiments. Others use actin or other ‚house-keeping' proteins with high expression levels. However, their relatively much higher expression levels compared to the protein of interest lead to an overloading of the membrane with the control protein. These proteins form a pile and the antibodies are only able to bind to the proteins on top of the pile. Therefore, the protein-to-signal ratio is not linear and the protein signal intensity is also effected by the shape of the protein band (distribution of two much protein on the membrane). It is better to use a protein as a reference protein in such experiments, which has an expression level comparable to the protein of interest. Another reviewer mentioned the inconsistency of these data.
I suggest that you present all western-blot experiments, which you used for your quantifications in the supplements. This would allow the readers to verify the quality of the experiments themselves and you would be able to convince readers, who may question the conclusions you draw from your data.

I think that version 3 of your manuscript is much improved over your previous versions. The additional work you invested should pay off.

Referee #3:

The authors have addressed our previous concerns.

Responses to the reviewer (EMBOR-2025-63143V4)

In response to our revision, the reviewers have provided further suggestions. They are listed below along with our point-by-point responses.

Referee #1:

Review of the manuscript: Phosphoglycerate kinase 1 cooperates with AP-2 complex to promote endocytosis Authors: Jia-Wei Hsu, Shao-Ling Chu, Yu-Tzu Chang, Che-Yu Hung, and Chia-Jung Yu Manuscript Number: EMBOR-2025-63143V3

Dear authors,

The discussion of your PGK1 ‚DKF' data is much improved in the current version of the manuscript. You may have pointed out that this motif is not a canonical adaptin ‚ear'-domain ‚DKF' motif, which was the reason why you mutated these 3 residues in PGK1. You could have introduced a nomenclature for the PGK1 ‚DKF' motif, which should be able to prevent a confusion.

We thank the reviewer for the suggestion. We have now revised the statement to point out the DKF motif of PGK1 is not canonical DxF motif.

I want to point out two problems I have with your presentation of your microscopic data and your semi-quantitative western-blot experiments. Some of these were already mentioned by another reviewer of the second version of your manuscript.

1. You show in your microscopic analysis only single cells. You should show an overview of the slide which shows several cells instead and one of these cells you should enlarge. This is the common way to present such data. You may update your supplements accordingly.

We thank the reviewer for the suggestion. We have revised the appendix microscopic images to include an overview of the slide accordingly.

2. You have used tubulin as a control protein in your semi-quantitative western-blot experiments. Others use actin or other ‚house-keeping' proteins with high expression levels. However, their relatively much higher expression levels compared to the protein of interest lead to an overloading of the membrane with the control protein. These proteins form a pile and the antibodies are only able to bind to the proteins on top of the pile. Therefore, the protein-to-signal ratio is not linear and the protein signal intensity is also effected by the shape of the protein band (distribution of two much protein on the membrane). It is better to use a protein as a reference protein in such experiments, which has an expression level comparable to the protein of interest.

Another reviewer mentioned the inconsistency of these data.

I suggest that you present all western-blot experiments, which you used for your quantifications in the supplements. This would allow the readers to verify the quality of the experiments themselves and you would

be able to convince readers, who may question the conclusions you draw from your data.

We thank the reviewer for the suggestion. We have now included western blot images for all experiments used for the quantitative analyses in the appendix.

I think that version 3 of your manuscript is much improved over your previous versions. The additional work you invested should pay off.

Referee #3:

The authors have addressed our previous concerns.

Prof. Jia-Wei Hsu
National Taiwan University
Institute of Biochemical Sciences
No. 1, Sec. 4, Roosevelt Road
IBS N304
Taipei 10617
Taiwan

Dear Prof. Hsu,

I am very pleased to accept your manuscript for publication in the next available issue of EMBO reports. Thank you for your contribution to our journal.

Your manuscript will be processed for publication by EMBO Press. It will be copy edited and you will receive page proofs prior to publication. Please note that you will be contacted by Springer Nature Author Services to complete licensing and payment information.

You may qualify for financial assistance for your publication charges - either via a Springer Nature fully open access agreement or an EMBO initiative. Check your eligibility: https://link.springer.com/journal/44319/how-to-publish-with-us

Should you be planning a Press Release on your article, please notify the production team via this form in order to coordinate publication and release dates: https://forms.cloud.microsoft/e/eH4A8JVP6g

If you have any questions, please do not hesitate to contact the Editorial Office. Thank you for your contribution to EMBO Reports.

Yours sincerely,

Martina Rembold, PhD
Senior Editor
EMBO reports

>>> Please note that it is EMBO Reports policy for the transcript of the editorial process (containing referee reports and your response letter) to be published as an online supplement to each paper. If you do NOT want this, you will need to inform the Editorial Office via email immediately. More information is available here: https://link.springer.com/partners/embo-press/editorial-policies#Peer%20review

EMBO Press Author Checklist

Corresponding Author Name: Jia-Wei Hsu	
Journal Submitted to: EMBO Reports	
Manuscript Number: EMBOR-2025-63143V4	

USEFUL LINKS FOR COMPLETING THIS FORM
The EMBO Journal - Author Guidelines
EMBO Reports - Author Guidelines
Molecular Systems Biology - Author Guidelines
EMBO Molecular Medicine - Author Guidelines

Reporting Checklist for Life Science Articles (updated January
This checklist is adapted from Materials Design Analysis Reporting (MDAR) Checklist for Authors. MDAR establishes a minimum set of requirements in transparent reporting in the life sciences (see Statement of Task: 10.31222/osf.io/9sm4x). Please follow the journal's guidelines in preparing your
Please note that a copy of this checklist will be published alongside your article.

Abridged guidelines for figures
1. Data
The data shown in figures should satisfy the following conditions:
- ➞ the data were obtained and processed according to the field's best practice and are presented to reflect the results of the experiments in an accurate and unbiased manner.
- ➞ ideally, figure panels should include only measurements that are directly comparable to each other and obtained with the same assay.
- ➞ plots include clearly labeled error bars for independent experiments and sample sizes. Unless justified, error bars should not be shown for technical
- ➞ if n<5, the individual data points from each experiment should be plotted. Any statistical test employed should be justified.
- ➞ Source Data should be included to report the data underlying figures according to the guidelines set out in the authorship guidelines on Data

2. Captions
Each figure caption should contain the following information, for each panel where they are relevant:
- ➞ a specification of the experimental system investigated (eg cell line, species name).
- ➞ the assay(s) and method(s) used to carry out the reported observations and measurements.
- ➞ an explicit mention of the biological and chemical entity(ies) that are being measured.
- ➞ an explicit mention of the biological and chemical entity(ies) that are altered/varied/perturbed in a controlled manner.
- ➞ the exact sample size (n) for each experimental group/condition, given as a number, not a range;
- ➞ a description of the sample collection allowing the reader to understand whether the samples represent technical or biological replicates (including how many animals, litters, cultures, etc.).
- ➞ a statement of how many times the experiment shown was independently replicated in the laboratory.
- ➞ definitions of statistical methods and measures:
  - common tests, such as t-test (please specify whether paired vs. unpaired), simple χ2 tests, Wilcoxon and Mann-Whitney tests, can be unambiguously identified by name only, but more complex techniques should be described in the methods section;
  - are tests one-sided or two-sided?
  - are there adjustments for multiple comparisons?
  - exact statistical test results, e.g., P values = x but not P values < x;
  - definition of 'center values' as median or average;
  - definition of error bars as s.d. or s.e.m.

Please complete ALL of the questions below.
Select "Not Applicable" only when the requested information is not relevant for your study.

Materials

Newly Created Materials	Information included in the manuscript?	In which section is the information available? (Reagents and Tools Table, Materials and Methods, Figures, Data Availability Section)
New materials and reagents need to be available; do any restrictions apply?	Not Applicable	

Antibodies	Information included in the manuscript?	In which section is the information available? (Reagents and Tools Table, Materials and Methods, Figures, Data Availability Section)
For **antibodies** provide the following information:<br>- Commercial antibodies: RRID (if possible) or supplier name, catalogue number and or/clone number<br>- Non-commercial: RRID or citation	Yes	Reagents and Tools Table, Materials and Methods

DNA and RNA sequences	Information included in the manuscript?	In which section is the information available? (Reagents and Tools Table, Materials and Methods, Figures, Data Availability Section)
**Short novel DNA or RNA including primers, probes**: provide the sequences.	Yes	Reagents and Tools Table, Materials and Methods

Cell materials	Information included in the manuscript?	In which section is the information available? (Reagents and Tools Table, Materials and Methods, Figures, Data Availability Section)
**Cell lines:** Provide species information, strain. Provide accession number in repository **OR** supplier name, catalog number, clone number, and/**OR** RRID.	Yes	Materials and Methods
**Primary cultures:** Provide species, strain, sex of origin, genetic modification status.	Not Applicable	
Report if the cell lines were recently **authenticated** (e.g., by STR profiling) and tested for mycoplasma contamination.	Not Applicable	

Experimental animals	Information included in the manuscript?	In which section is the information available? (Reagents and Tools Table, Materials and Methods, Figures, Data Availability Section)
**Laboratory animals or Model organisms:** Provide species, strain, sex, age, genetic modification status. Provide accession number in repository **OR** supplier name, catalog number, clone number, **OR** RRID.	Not Applicable	
**Animal observed in or captured from the field:** Provide species, sex, and age where possible.	Not Applicable	
Please detail **housing and husbandry conditions**.	Not Applicable	

Plants and microbes	Information included in the manuscript?	In which section is the information available? (Reagents and Tools Table, Materials and Methods, Figures, Data Availability Section)
**Plants:** provide species and strain, ecotype and cultivar where relevant, unique accession number if available, and source (including location for collected wild specimens).	Not Applicable	
**Microbes:** provide species and strain, unique accession number if available, and source.	Not Applicable	

Human research participants	Information included in the manuscript?	In which section is the information available? (Reagents and Tools Table, Materials and Methods, Figures, Data Availability Section)
If collected and within the bounds of privacy constraints report on age, sex and gender or ethnicity for all study participants.	Not Applicable	

Core facilities	Information included in the manuscript?	In which section is the information available? (Reagents and Tools Table, Materials and Methods, Figures, Data Availability Section)
If your work benefited from core facilities, was their service mentioned in the acknowledgments section?	Yes	Acknowledgement

Design

Study protocol	Information included in the manuscript?	In which section is the information available? (Reagents and Tools Table, Materials and Methods, Figures, Data Availability Section)
If study protocol has been **pre-registered, provide DOI in the manuscript**. For clinical trials, provide the trial registration number **OR** cite DOI.	Not Applicable	
Report the **clinical trial registration number** (at ClinicalTrials.gov or equivalent), where applicable.	Not Applicable	

Laboratory protocol	Information included in the manuscript?	In which section is the information available? (Reagents and Tools Table, Materials and Methods, Figures, Data Availability Section)
Provide DOI OR other citation details if **external detailed step-by-step protocols** are available.	Not Applicable	

Experimental study design and statistics	Information included in the manuscript?	In which section is the information available? (Reagents and Tools Table, Materials and Methods, Figures, Data Availability Section)
Include a statement about **sample size** estimate even if no statistical methods were used.	Yes	Figure Legends
Were any steps taken to minimize the effects of subjective bias when allocating animals/samples to treatment (e.g. **randomization procedure**)? If yes, have they been described?	Not Applicable	
Include a statement about **blinding** even if no blinding was done.	Not Applicable	
Describe **inclusion/exclusion criteria** if samples or animals were excluded from the analysis. Were the criteria pre-established?<br><br>If sample or data points were omitted from analysis, report if this was due to attrition or intentional exclusion and provide justification.	Not Applicable	
For every figure, are **statistical tests** justified as appropriate? Do the data meet the assumptions of the tests (e.g., normal distribution)? Describe any methods used to assess it. Is there an estimate of variation within each group of data? Is the variance similar between the groups that are being statistically compared?	Not Applicable	

Sample definition and in-laboratory replication	Information included in the manuscript?	In which section is the information available? (Reagents and Tools Table, Materials and Methods, Figures, Data Availability Section)
In the figure legends: state number of times the experiment was **replicated** in laboratory.	Yes	Figure Legends
In the figure legends: define whether data describe **technical or biological replicates**.	Yes	Figure Legends

Ethics

Ethics	Information included in the manuscript?	In which section is the information available? (Reagents and Tools Table, Materials and Methods, Figures, Data Availability Section)
Studies involving **human participants**: State details of **authority granting ethics approval** (IRB or equivalent committee(s), provide reference number for approval.	Not Applicable	
Studies involving **human participants**: Include a statement confirming that **informed consent** was obtained from all subjects and that the experiments conformed to the principles set out in the WMA Declaration of Helsinki and the Department of Health and Human Services Belmont Report.	Not Applicable	
Studies involving **human participants:** For publication of **patient photos**, include a statement confirming that consent to publish was obtained.	Not Applicable	
Studies involving experimental **animals**: State details of **authority granting ethics approval** (IRB or equivalent committee(s), provide reference number for approval. Include a statement of compliance with ethical regulations.	Not Applicable	
Studies involving **specimen and field samples:** State if relevant **permits** obtained, provide details of authority approving study; if none were required, explain why.	Not Applicable	

Dual Use Research of Concern (DURC)	Information included in the manuscript?	In which section is the information available? (Reagents and Tools Table, Materials and Methods, Figures, Data Availability Section)
Could your study fall under dual use research restrictions? Please check biosecurity documents and list of **select agents and toxins** (CDC): https://www.selectagents.gov/sat/list.htm	Not Applicable	
If you used a select agent, is the security level of the lab appropriate and reported in the manuscript?	Not Applicable	
If a study is subject to dual use research of concern regulations, is the name of the **authority granting approval and reference number** for the regulatory approval provided in the manuscript?	Not Applicable	

Reporting

The MDAR framework recommends adoption of discipline-specific guidelines, established and endorsed through community initiatives. Journals have their own policy about requiring specific guidelines and recommendations to complement MDAR.

Adherence to community standards	Information included in the manuscript?	In which section is the information available? (Reagents and Tools Table, Materials and Methods, Figures, Data Availability Section)
State if relevant guidelines or checklists (e.g., **ICMJE, MIBBI, ARRIVE, PRISMA**) have been followed or provided.	Not Applicable	
For **tumor marker prognostic studies,** we recommend that you follow the **REMARK** reporting guidelines (see link list at top right). See author guidelines, under 'Reporting Guidelines'. Please confirm you have followed these guidelines.	Not Applicable	
For **phase II and III randomized controlled trials**, please refer to the **CONSORT** flow diagram (see link list at top right) and submit the CONSORT checklist (see link list at top right) with your submission. See author guidelines, under 'Reporting Guidelines'. Please confirm you have submitted this list.	Not Applicable	

Data Availability

Data availability	Information included in the manuscript?	In which section is the information available? (Reagents and Tools Table, Materials and Methods, Figures, Data Availability Section)
Have **primary datasets** been deposited according to the journal's guidelines (see 'Data Deposition' section) and the respective accession numbers provided in the Data Availability Section?	Not Applicable	
Were **human clinical and genomic datasets** deposited in a public access-controlled repository in accordance to ethical obligations to the patients and to the applicable consent agreement?	Not Applicable	
Are **computational models** that are central and integral to a study available without restrictions in a machine-readable form? Were the relevant accession numbers or links provided?	Not Applicable	
If publicly available data were reused, provide the respective **data citations in the reference list**.	Not Applicable	
